# Taxonomic revision of the *Pheidole megacephala* species-group (Hymenoptera, Formicidae) from the Malagasy Region

**DOI:** 10.7717/peerj.13263

**Published:** 2022-04-26

**Authors:** Sebastian Salata, Brian L. Fisher

**Affiliations:** 1 California Academy of Sciences, San Francisco, CA, USA; 2 Myrmecological Laboratory, Department of Biodiversity and Evolutionary Taxonomy, University of Wroclaw, Wroclaw, Lower Silesia, Poland

**Keywords:** Biodiversity, Entomology, Taxonomy, Biogeography, Phylogeny, Invasive species, Madagascar

## Abstract

**Background:**

The Malagasy Region, one of the top megadiversity regions, hosts one of the highest numbers of endemic and threatened organisms on earth. One of the most spectacular examples of ant radiation on the island has occurred in the hyperdiverse genus *Pheidole*. To this date, there are 135 described Madagascan *Pheidole* divided into 16 species-groups, and 97% of Malagasy species are endemic to the island. This study is a taxonomic revision of the *Pheidole megacephala* group, one of only two species-groups comprising a combination of native, endemic taxa and widely distributed introduced species.

**Methods:**

The diversity of the Malagasy members of the *megacephala* group was assessed via application of qualitative morphological and DNA sequence data. Qualitative, external morphological characteristics (*e.g.,* head shape, gaster sculpture, body colouration) were evaluated in order to create a priori grouping hypotheses, and confirm and improve species delimitation. Mitochondrial DNA sequences from cytochrome oxidase I (COI) gene fragments were analyzed to test the putative species previously delimited by morphological analyses.

**Results:**

We recognize three species belonging to the *megacephala* group: *P. megacephala* (Fabricius, 1793), *P. megatron* Fischer & Fisher, 2013 and *P. spinosa* Forel, 1891 **stat. nov.** *Pheidole spinosa* is redescribed and elevated to the species level. The following names are recognized as junior synonyms of *P. spinosa*: *P. megacephala scabrior* Forel, 1891 **syn. nov.**, *P. picata* Forel, 1891 **syn. nov.**, *P. picata gietleni* Forel, 1905 **syn. nov.**, *P. picata bernhardae* Emery, 1915 **syn. nov.**, and *P. decepticon* Fischer & Fisher, 2013 **syn. nov.** The results are supplemented with an identification key to species for major workers of the *megacephala* group, high-resolution images for major and minor workers, and comments on the distribution and biology of all Malagasy members of the group. Our study revealed that *Pheidole megacephala*, a species listed among the 100 worst invasive species worldwide, occurs in both natural and disturbed sites in the Malagasy region. The two remaining members of the *megacephala* group, most likely endemic to this region, are also present in anthropogenic habitats and often co-occur with *P. megacephala*. It appears that the Malagasy members of the group are generalists and dominant in anthropogenic habitats. Additionally, we documented the presence of supermajors in colonies of *P. spinosa*—a phenomenon previously not known for this group.

## Introduction

*Pheidole* Westwood, 1839 is the most speciose ant genus globally. It comprises 1167 described extant species known from all biogeographic regions except the Antarctic (Bolton, 2021). The diversity of Malagasy *Pheidole* was overlooked for decades until recent taxonomic work (Fischer & Fisher, 2013; Salata & Fisher, 2020a; Salata & Fisher, 2020b; Salata & Fisher, 2020c; Salata & Fisher, 2021). Most recent discoveries confirm a globally exceptional level of endemism among Malagasy *Pheidole*. To date, there are 135 *Pheidole* species from this region, of which 97% are considered endemic. This tally, however, is still not complete, and we estimate that approximately 20 taxa await descriptions in forthcoming revisions.

Based on the most recent data (Fischer et al., 2020; Economo et al., 2019) *Pheidole* diversified in the tropics of the New World approximately 29 Ma. Around 13 Ma, a single lineage colonized the Old World and experienced another burst of diversification. Madagascar was colonized around 10 Ma and almost all its species are part of a single endemic radiation. However, the *megacephala* group colonized the Malagasy region later, around 5 Ma. Some insights suggest that the smaller islands are occupied by species of mixed African and Asian origin (Economo et al., 2019).

Taxonomic knowledge of Malagasy *Pheidole* has greatly improved in recent times, and only two species-groups have pending revisions: *lucida* and *megacephala*. The *lucida* species-group consists of only native species that most likely are social parasites of other *Pheidole* species; its distribution is limited to Madagascar (Fischer et al., 2020). The *megacephala* species-group is a combination of two native and possibly endemic taxa, and one widely distributed invasive species. *Pheidole megacephala* is listed among the 100 worst invasive species (GISD, 2021) and is also the most widespread species of the entire genus (Economo et al., 2019). However, this species was described from Mauritius, and was collected from both natural and disturbed sites in the Malagasy region. In Madagascar, its impact on the native fauna in natural habitats has not been studied and it is unknown whether *P. megacephala* expresses the same destructive impact in Madagascar as in other regions. *Pheidole spinosa* and *P. megatron*, both most likely native and endemic to this region, are also dominant in anthropogenic habitats and often co-occur with *P. megacephala*. However, *P. spinosa* commonly occurs also in natural habitats of Madagascar.

Overall, the *megacephala* species-group consists of a number of described species and infraspecific taxa and is one of the most taxonomically challenging within *Pheidole.* Except for *P. megacephala*, which is one of the most widespread invasive species, all species belonging to this group have distribution restricted to Afrotropics (∼12 species and subspecies) and Malagasy (three species) (Bolton, 2021). The relationship between taxa from these two regions is unknown but based on their morphology the native species form two distinct complexes of species in each region. Below, we present a taxonomic revision of the *megacephala* species-group from the Malagasy region and discuss general trends in distribution and morphology observed among its members.

## Materials & Methods

Ant samples used in this study comply with the regulations for export and exchange of research samples outlined in the Convention on Biological Diversity and the Convention on International Trade in Endangered Species of Wild Fauna and Flora. For fieldwork conducted in Madagascar, Comoros, Juan de Nova Island, Mauritius, Mayotte, Reunion and Seychelles, permits to research, collect, and export ants were obtained from the Ministry of Environment and Forest as part of an ongoing collaboration between the California Academy of Sciences and the Ministry of Environment and Forest, Madagascar National Parks, and Parc Botanique et Zoologique de Tsimbazaza (Madagascar), Centre National de Documentation et de Recherche Scientifique (Comoros), Terres Australes et Antarctiques Françaises and Direction de la Conservation du Patrimoine Naturel (Juan de Nova Island), Forestry Service and National Parks and Conservation Service (Mauritius), Naturalistes de Mayotte (Mayotte), Insectarium de la Reunion (Reunion), and Seychelles Bureau of Standards and National Park Authority (Seychelles). Authorization for export was provided by the Director of Natural Resources.

The present study was conducted on specimens collected in Madagascar and nearby islands in the Southwest Indian Ocean, and deposited in the California Academy of Sciences, San Francisco, California, U.S.A. All specimen data are listed in Table S1 and freely accessible on AntWeb (http://www.antweb.org). Each specimen used in this study can be traced by a unique specimen identifier affixed to the pin (*e.g.*, CASENT0071790).

Repositories. Collections are referred to by the following acronyms:

**CAS**–California Academy of Sciences, San Francisco, California, USA;

**MHNG**–Muséum d’Historie Naturelle, Geneva, Switzerland.

Material was analyzed as previously described in the revision of the Malagasy *fervens* species-group (Salata & Fisher, 2021). Specifically, all observations and measurements were taken using a pin-holding stage, permitting rotations around the X, Y, and Z axes at magnifications from 32 × to 100 × with a Leica MZ12.5 microscope and an orthogonal crosshair micrometer, at an accuracy of 0.01 mm to approximately 0.005 mm. All measurements are presented in mm units as minimum and maximum values, with the arithmetic mean in parentheses. Photographs were taken using a JVC KY-75 or Leica DFC450 digital camera with a Leica Z16 APO microscope and Leica Application Suite software (v3.8). Unless stated otherwise, photographs were taken by the senior author, and material was collected by B. L. Fisher and his collaborators and stored at CASC. Images of specimens and data of all pinned specimens examined in the present contribution are available online on AntWeb (http://www.AntWeb.org) and accessible using the unique CASENT identifying specimen code. Measurements and indices are in line with Salata & Fisher (2020a), Salata & Fisher (2020b), Salata & Fisher (2020c) and Salata & Fisher (2021) and are mostly the same as in Longino (2009) and Longino (2019) and several other revisions (Eguchi, 2008; Fischer, Hita Garcia & Peters, 2012; Fischer & Fisher, 2013; Wang, Yamada & Eguchi, 2018). The general morphological terminology follows Wilson (2003), Longino (2009) and Longino (2019). The surface sculpturing glossary follows Harris (1979).

Pilosity inclination degree follows that used in Wilson (1955). Appressed (0–5°) hairs run parallel or nearly parallel to the body surface. Decumbent hairs stand 10–40°, subdecumbent hair stand ∼45° from the surface, suberect hairs bend about 10–20° from vertical, and erect hairs stand vertical or nearly vertical.

### DNA based-species delimitation analysis

We analyzed 658 base pairs (bp) of the mitochondrial cytochrome oxidase I (COI) gene from 204 specimens of the Malagasy members of the *megacephala* group (Table S2). Selected COI gene sequences were obtained from material that comes from nest samples consisting of major and minor workers. DNA was extracted using the specimen’s legs, allowing preservation of vouchers. DNA extraction and COI sequencing were performed at the University of Guelph (Ontario, Canada), following the protocol described in Fisher & Smith (2008). Sequences were aligned using Geneious Prime (Masters, Fan & Ross, 2011). The final alignment is available as Table S3. To exclude redundancies in the matrix, we removed 102 duplicated haplotypes using the Alter (Glez-Peña et al., 2010) online platform (http://sing.ei.uvigo.es/ALTER/). We also excluded samples with too short or fragmentary sequences (14) and these with internal stop codons (3). The final simplified data matrix had 95 sequences consisting of 85 unique *Pheidole megacephala* group haplotypes and 10 outgroups.

ModelFinder Plus (Kalyaanamoorthy et al., 2017) implemented in IQ-TREE was used to identify the best nucleotide substitution model and partition scheme for the data under the Bayesian information criterion (BIC). The best fit model selected was TIM2+F+I+G4. ML analyses (the best tree and nodal support values) were performed using IQ-TREE with 10,000 ultrafast bootstrap replicates (UFBoot), which is computationally efficient and relatively unbiased (Hoang et al., 2018). Tree visualisation and rooting were done in FigTree v.1.4.4 (Rambaut, 2021).

### Species delimitation

Our recognition of species follows the biological species concept and species boundaries are based on comparative morphology and known geographic distributions of investigated taxa. Where sympatric populations exhibit consistently different phenotypes, they are considered different species.

Species delimitation analyses based on mitochondrial DNA sequences from cytochrome oxidase I (COI) gene fragments were carried out to test the putative species previously delimited by morphological analyses (Csősz, Loss & Fisher, 2021). Analyses were conducted with the Species Delimitation Plugin (SDP) (Masters, Fan & Ross, 2011), implemented in Geneious, using the following metrics: (i) monophyly; (ii) average intraspecific uncorrected pairwise distance (Intra Dist); (iii) average uncorrected pairwise distance between a putative species and its sister species (Inter Dist); (iv) PID Liberal (Ross, Murugan & Sibon Li, 2008); and (v) Rosenberg’s PAB statistics (Rosenberg, 2007).

PID Liberal measures the probability of a correct identification of an unknown specimen as a member of the putative species or its sister species, while Rosenberg’s PAB is the probability of reciprocal monophyly by random chance. Thus, we expect valid species to be monophyletic, with overall intraspecific distance smaller than interspecific, high PID Liberal values, and small values of Rosenberg’s PAB.

### Measurements and indices

**Figure 1:**
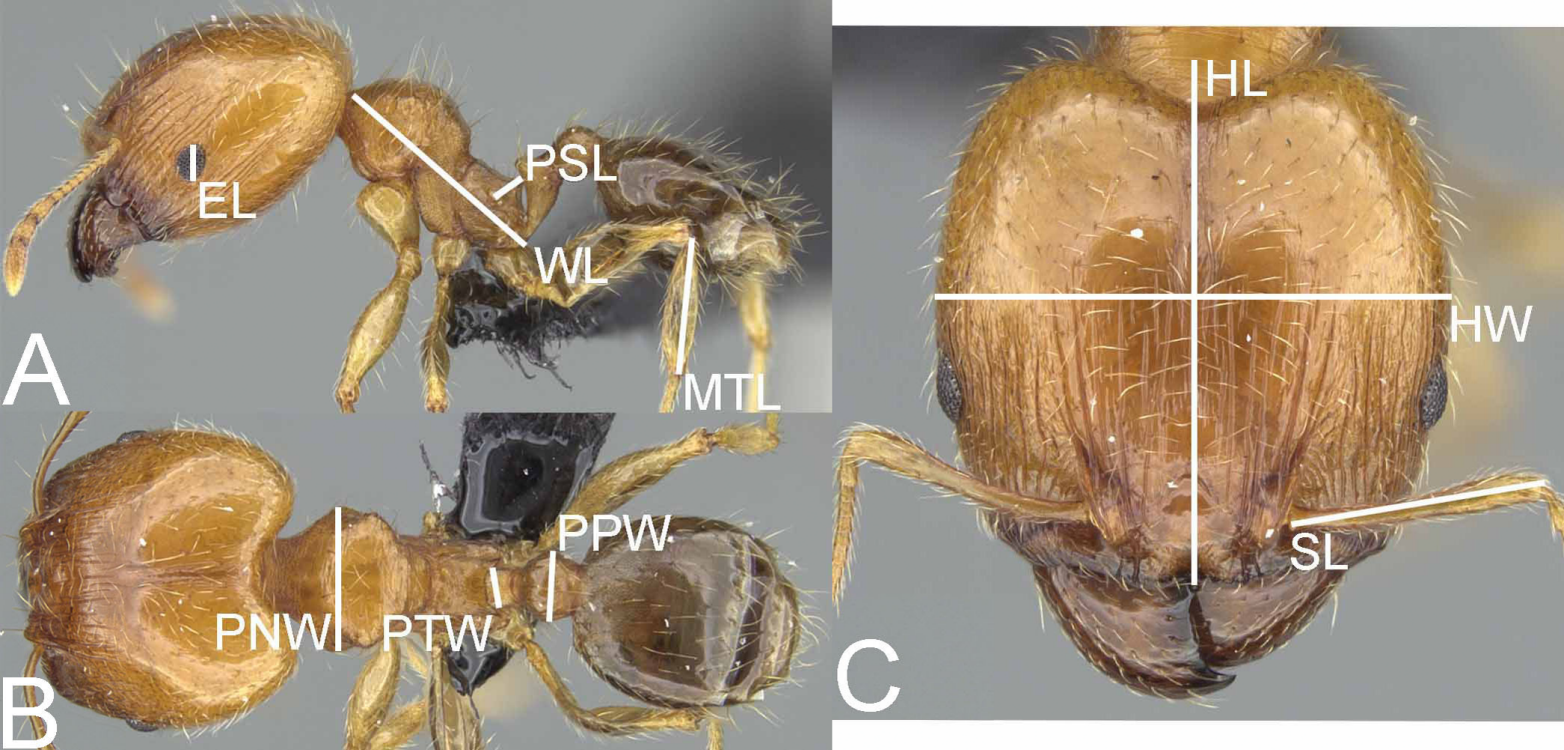
*Pheidole megatron*, illustrations of measurements (A–C). Profile (A). Dorsal view (B). Full face view (C). Photo credit: Sebastian Salata.

Measurements (Fig. 1)

Performed measurements are identical as these described in the previously published revisions of the Malagasy *Pheidole* species-group (Salata & Fisher, 2020a; Salata & Fisher, 2020b; Salata & Fisher, 2020c; Salata & Fisher, 2021).

**EL**—eye length; measured along the maximum vertical diameter of the eye;

**HL**—head length; maximum distance from the midpoint of the anterior clypeal margin to the midpoint of the posterior margin of the head, measured in full-face view; in majors from midpoint of tangent between anteriormost position of clypeus to midpoint of tangent between posteriormost projection of the vertex;

**HW**—head width; measured in full-face view, at widest point of the head, directly above the eyes;

**MTL**—metatibia length; straight line length of the metatibia measured from the constriction immediately before its proximal insertion to its distalmost point, excluding the bristles or spines;

**PNW**—pronotum width; maximum width of promesonotum measured in dorsal view;

**PPW**—postpetiole width; maximum width of postpetiole in dorsal view;

**PSL**—propodeal spine length; measured from the center of the propodeal spiracle to the tip of the propodeal spine in lateral view;

**PTW**—petiole width; maximum width of petiole in dorsal view;

**SL**—scape length; maximum straight-line length of scape excluding the basal condylar bulb;

**WL**—mesosoma length (Weber’s length); diagonal length of mesosoma in lateral view from the anterior point of the pronotal slope and excluding the neck, to the posteroventral margin of the propodeum.

Indices

**CI**—cephalic index: HW/HL * 100;

**MTI**—tibia index: MTL/HW * 100;

**SI**—scape index: SL/HW * 100;

**PNI**—pronotum index: PNW/HW * 100;

**PPI**—postpetiole width index: PPW/PTW * 100;

**PSLI**—propodeal spine index: PSL/HW * 100.

Abbreviations

**m**.—male; **q**.—gyne; **s.**—major worker; **w**.—minor worker.

**Figure 2:**
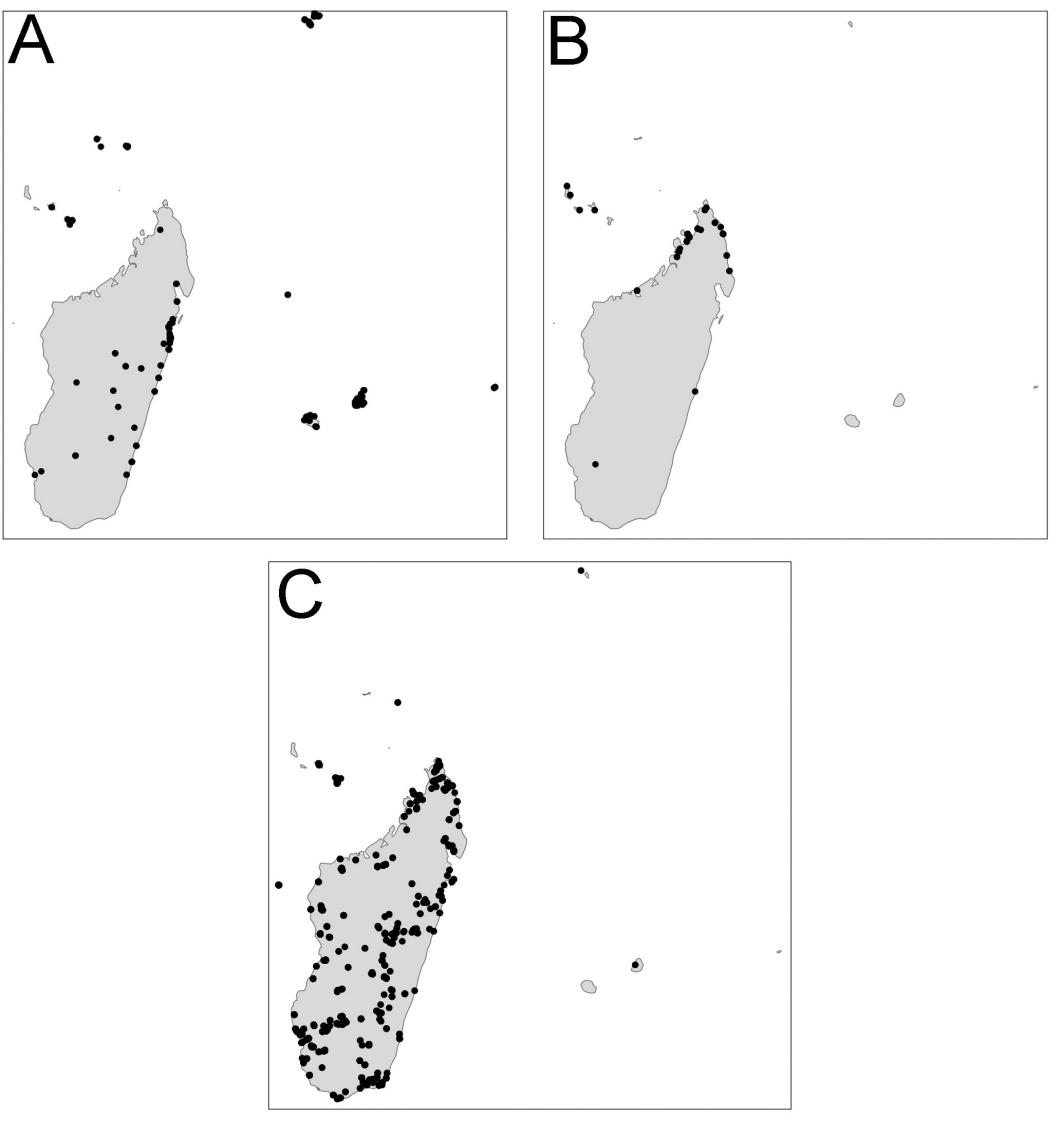
Distribution. *Pheidole megacephala* (A), *P. megatron* (B), *P. spinosa* (C).

Distribution maps (Fig. 2) were generated using tmap v2.2 package on R v3.5. R Core Team (Tennekes, 2018).

## Results

### Revision of the *Pheidole megacephala* group from the Malagasy Region

**Figure 3:**
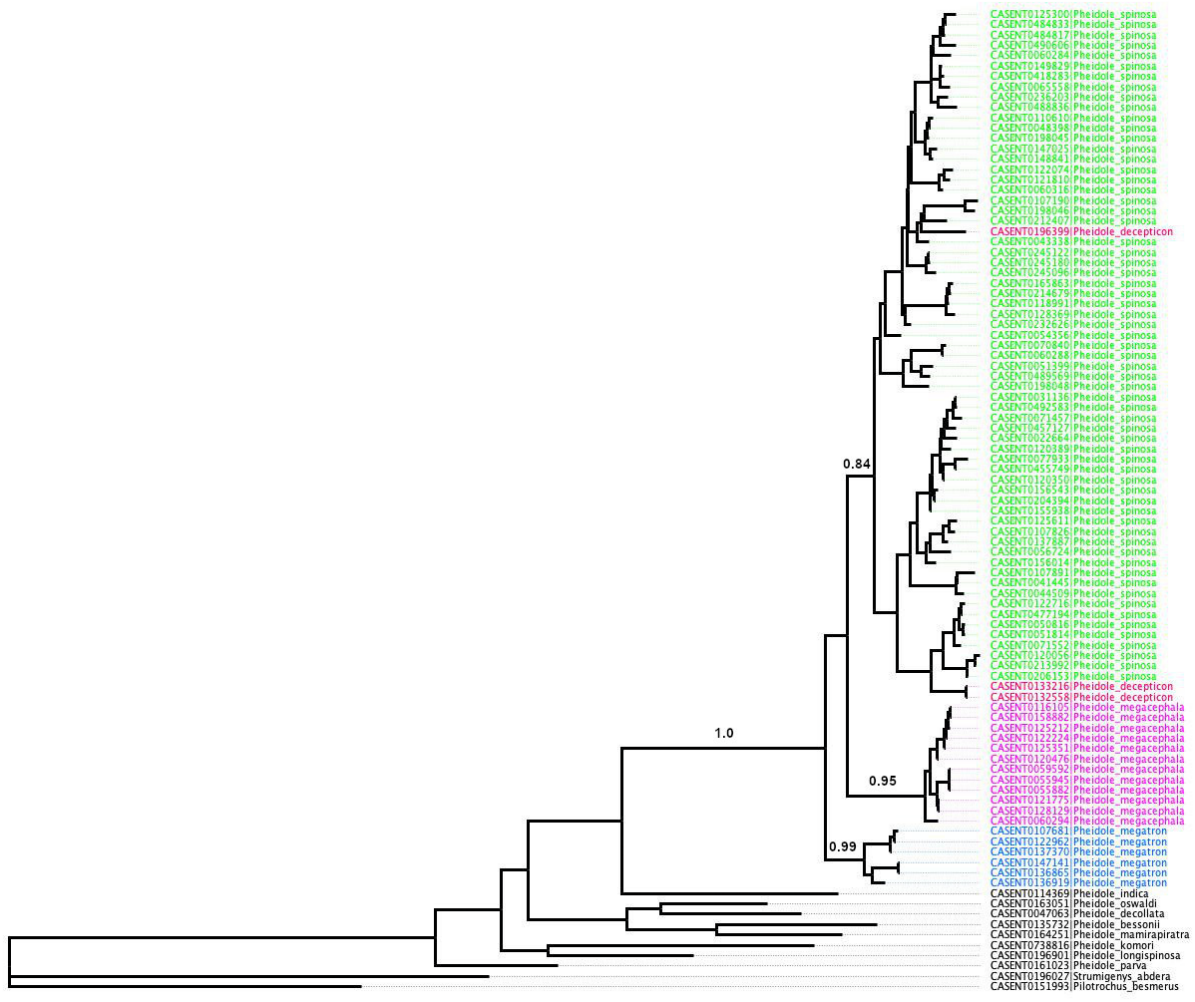
ML phylogeny of *Pheidole* COI sequences. Support values represent maximum likelihood bootstrap.

All putative species also showed satisfactory results for all DNA species delimitation criteria used (Fig. 3; Table 1). All clusters proved to be monophyletic, with intraspecific distance varying from 2.0% (*P. megacephala*) to 10.7% (*P. spinosa*) falling outside the range of interspecific divergence (from 17.1% to 17.5%), average PID Liberal values ranging from 0.95 (*P. spinosa* and *P. megatron*) to 0.98 (*P. megacephala*) and Rosenberg’s PAB values lower than 0.0001. Despite the relatively high intraspecific distance noted in *P. spinosa* we could not find any morphological or geographical distinction between the two branches observed in the phylogenetic tree of *P. spinosa* (Fig. 3). We hypothesize ongoing divergence of its populations, as the *megacephala* group colonized the Malagasy quite recently (around 5 Ma) (Economo et al., 2019).

### Synopsis of members of the *Pheidole megacephala* species-group from the Malagasy Region

**Table:** 

*Pheidole megacephala* (Fabricius, 1793)
*Pheidole megatron*Fischer & Fisher, 2013
*Pheidole spinosa*Forel, 1891**stat. nov.**
=*Pheidole megacephala scabrior*Forel, 1891**syn. nov.**
=*Pheidole picata*Forel, 1891**syn. nov.**
=*Pheidole picata gietleni*Forel, 1905**syn. nov.**
=*Pheidole picata bernhardae*Emery, 1915**syn. nov.**
=* Pheidole decepticon*Fischer & Fisher, 2013**syn. nov.**

**Table 1:** Results from species delimitation plugin analyses for *Pheidole* putative species defined by morphological analyses.

Species	Monophyletic	Intra Dist (%)	Inter Dist Closest (%)	PID Liberal	Rosenberg’s P_**AB**_
*P. megacephala*	yes	2.0	17.1	0.98 (0.94–1.0)	5.00E−16
*P. spinosa*	yes	10.7	17.5	0.95 (0.92–0.98)	5.00E−16
*P. megatron*	yes	3.8	17.1	0.95 (0.85–1.0)	5.40E−11

**Figure 4:**
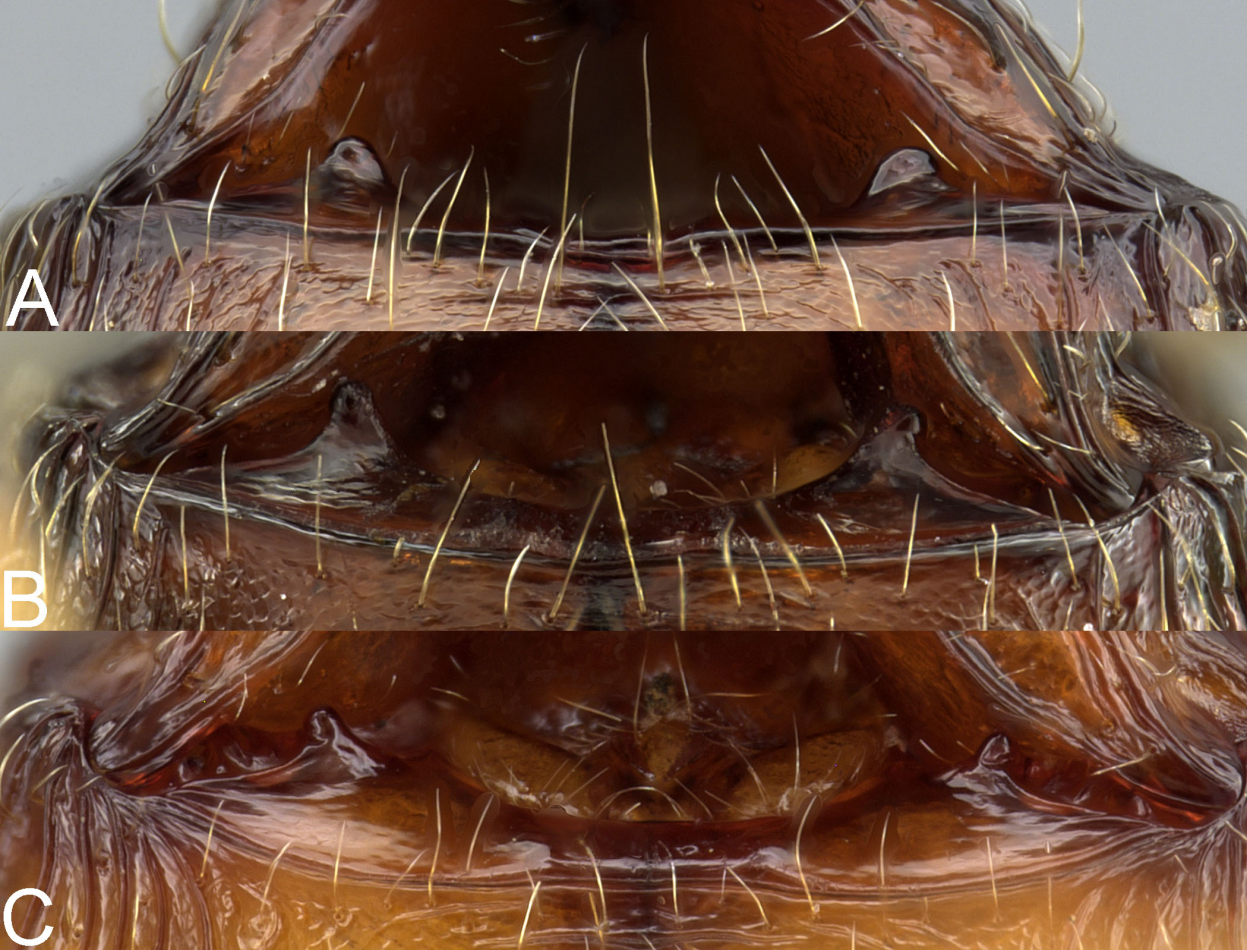
Major worker, hypostomal teeth. *Pheidole megacephala* (A), *P. megatron* (B), *P. spinosa* (C). Photo credit: Sebastian Salata.

**Diagnosis. Major and minor workers.** Postpetiole in profile with conspicuous ventral convexity (sometimes subtriangular in major workers). **Major workers.** Head subquadrate, suboval to cordate, sometimes elongated; antennal scrobe weak to inconspicuous; occipital lobes shiny and smooth or partially with indistinct microsculpture; frons with sparse and thick costulae and smooth to finely sculptured interspaces; inner hypostomal teeth indistinct or absent (Fig. 4); gaster smooth to partially shagreened. **Minor workers**. Head and promesonotum entirely or predominantly smooth; scape relatively short, surpassing the posterior head margin by two-fifths of its length; promesonotum in lateral view convex; propodeal spines minute to small.

The species of the *megacephala* species-group can be easily separated from other Malagasy species by a presence of conspicuous ventral convexity on the postpetiole. This character is unique for this group in this region and is present in both, major and minor workers.

**Note.** Due to the scope of this work, proposed diagnosis is restricted to Malagasy members of the *megacephala* species-group and should be used with caution when Afrotropical taxa are considered. A diagnosis of the African species the *megacephala* species-group is provided by Fischer, Hita Garcia & Peters (2012).

### Key to the Malagasy members of the *P. megacephala* group

Our survey revealed very high intraspecific variation in all morphological and morphometric characters within minor workers of the Malagasy members of the *megacephala* group. Fischer & Fisher (2013) separated minors based on the shape, length, and density of pilosity and some morphometric characters. However, they also suggested that the species determination should be based on the major caste. Our investigation supports this thesis. We could not find any stable characters that allow separation of species based on minor workers. Thus, we decided to include only major workers in the key. Due to presence of distinct characters in supermajors of *P. spinosa* we included them as a separate couplet in the key. Like Fischer & Fisher (2013), we also recommend performing species determinations based on a series of several major workers. However, we recognize that minor workers are the subcaste most frequently seen in the field. Thus, we recommend molecular identification through mitochondrial cytochrome oxidase I (COI) to confirm their identification.

**Table:** 

**Figure 5:**
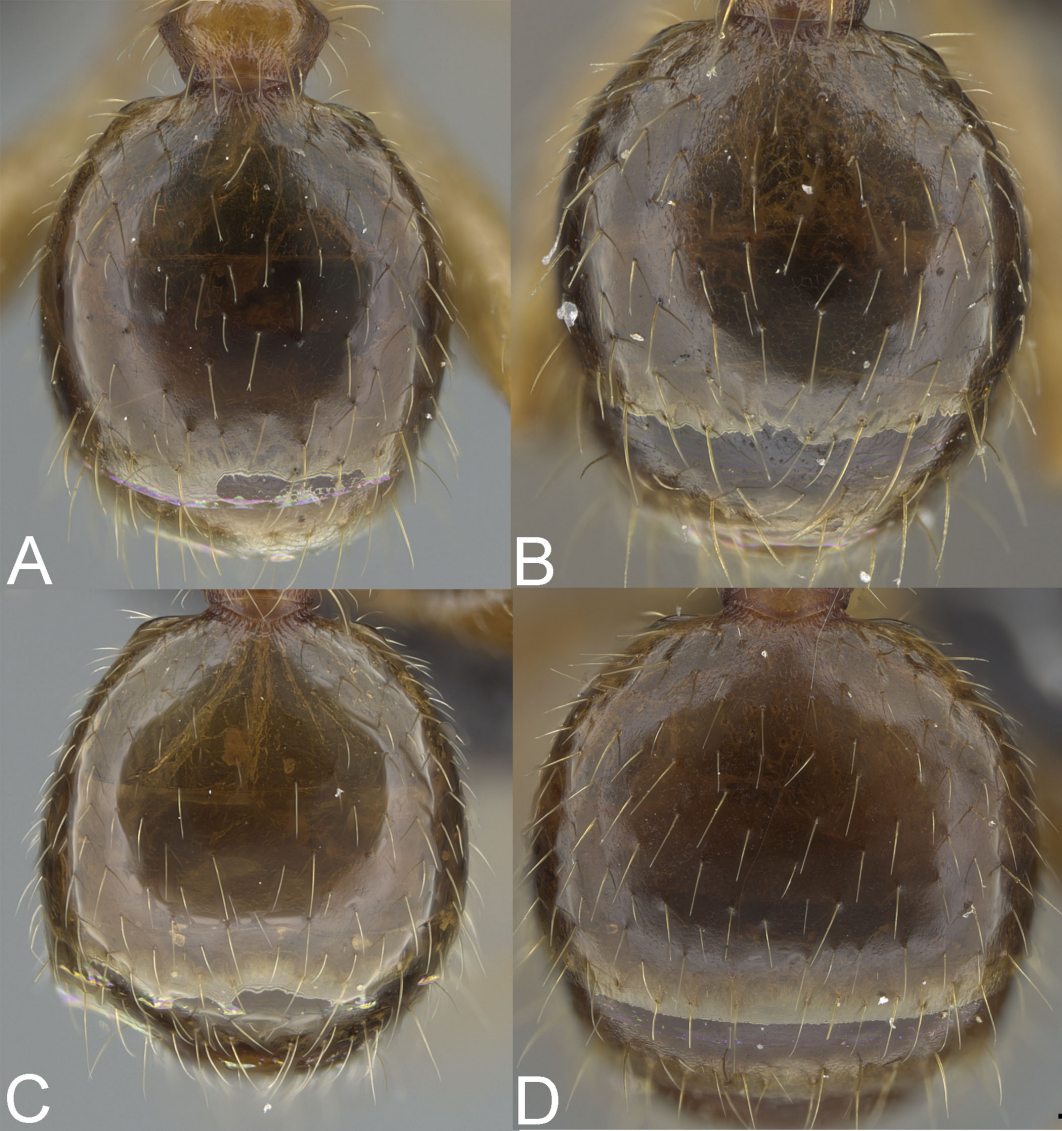
Major worker, gaster. *Pheidole megacephala* (A), *P. megatron* (B), *P. spinosa*, major worker (C), *P. spinosa*, supermajor worker (D). Photo credit: Sebastian Salata.

**Figure 6:**
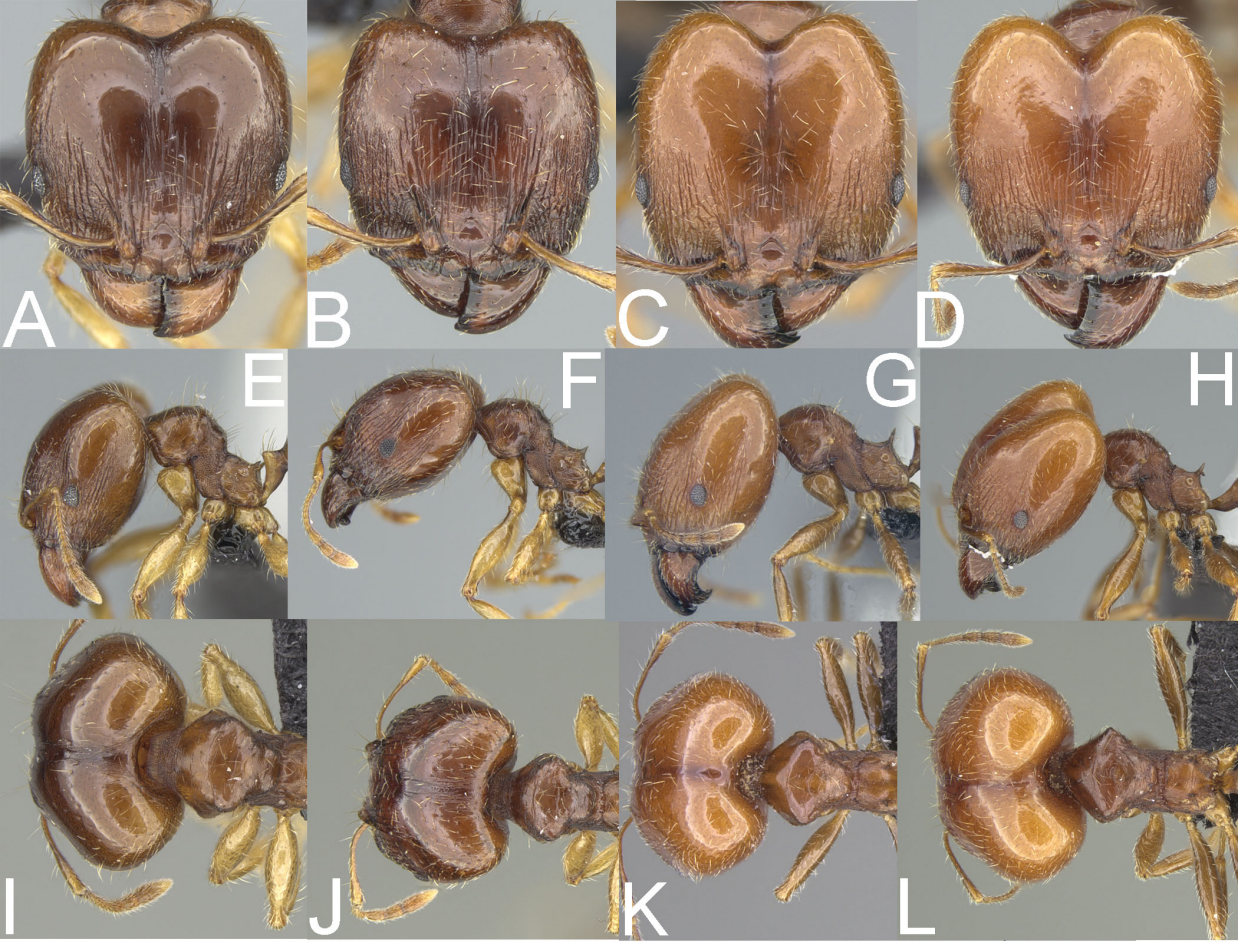
Major worker. *Pheidole megacephala*, head (A), profile (E), dorsal view (I). *P. megatron*, full face view (B), profile (F), dorsal view (J). *P. spinosa*, full face view (C), profile (G), dorsal view (K). Supermajor worker. *P. spinosa*, full face view (D), profile (H), dorsal view (L). Photo credit: Sebastian Salata.

1. Gaster smooth; head sub-rectangular and not or indistinctly widening posteriorly (Figs. 5C, 6C). ……….. ***Pheidole spinosa*, majors**
-. Gaster at least partially shagreened; head sub-rectangular to cordate, indistinctly to distinctly widening posteriorly (Figs. 5A–5B, 5D and 6A–6B, 6D) ……….. 2.
2. Occipital lobe smooth, propodeal spines thin and acute, humeral tubercle well developed (Figs. 6D, 6H, 6L). ……….. ***Pheidole spinosa*, supermajors**
-. Occipital lobe at least partially (sometimes indistinctly) sculptured, propodeal spines minute and with wide base, humeral tubercle not or weakly developed (Figs. 6A–6B, 6E–6F, 6I–6J). ………….. 3.
3. Occipital lobes entirely to predominantly shagreened, at least first gastral tergite shagreened, head sub-rectangular with slightly convex lateral sides (Figs. 5B, 6B, 6F, 6J). ………….. ***Pheidole megatron***
-. Occipital lobes predominantly smooth, first gastral tergite partially shagreened, head cordate and widening posteriorly (Figs. 5A, 6A, 6E, 6I). ………….. ***Pheidole megacephala***

**Table:** 

**Figure 7:**
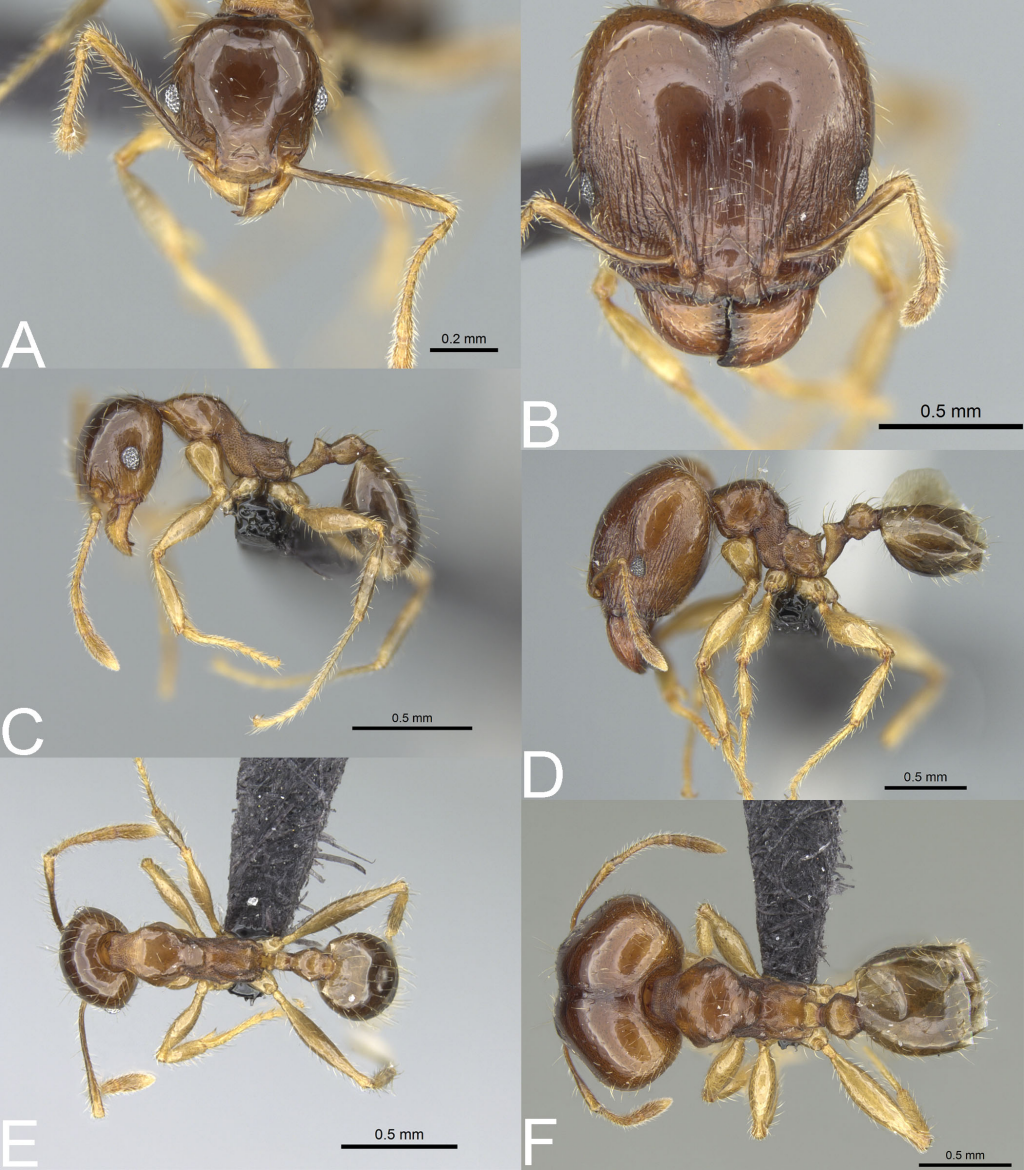
*Pheidole megacephala*. Full-face view (A), profile (C), and dorsal view (E) of minor worker and full-face view (B), profile (D), and dorsal view (F) of major worker. Photo credit: Sebastian Salata.

***Pheidole megacephala* (Fabricius, 1793)**
Figs. 5A, 6A, 6E, 7A–7F
*Formica megacephala*Fabricius, 1793: 361 (s.)
=*Pheidole agilis* (Smith, 1857): Eguchi, 2008: 56
=*Pheidole edax* (Forskål, 1775): Dalla Torre, 1893: 90
=*Pheidole janus* Smith, 1858: Dalla Torre, 1893: 92
=*Pheidole laevigata* (Smith, 1855): Roger, 1863: 30
=*Pheidole laevigata* Mayr, 1862: Roger, 1863: 30
=*Pheidole perniciosa* (Gerstäcker, 1859): Emery, 1915: 235
=*Pheidole pusilla* (Heer, 1852): Roger, 1863: 30
=*Pheidole suspiciosa* (Smith, 1859): (Donisthorpe, 1932): 455
=*Pheidole testacea* (Smith, 1858): Brown, 1981: 530
=*Pheidole trinodis* (Losana, 1834): Roger, 1863: 30

**Type material.** Neotype (designated by Fischer & Fisher, 2013): s., Mauritius, Camizard Mt., Bambous, 20.3328 S/57.723 E, 375 m, rainforest, ex rotten log, 27.v.2005, coll. B.L. Fisher et al., BLF12051, CASENT0104990 (CASC).

**Material investigated.** See Table S1.

**Geographic range.** Cosmopolitan species, recorded in the Malagasy Region from urban and anthropogenic sites on Comoros, Madagascar, Mauritius, Mayotte, Reunion, and Seychelles.

**Diagnosis. Major worker.** Head in full-face view cordate and widening posteriorly; in lateral view sub-oval; margins of the head with dense, subdecumbent to suberect pilosity; antennal scrobe indistinct; occipital lobe partially shagreened; inner hypostomal tooth absent or indistinct; outer hypostomal tooth lobe-like; median tooth absent; propodeal spine short to moderately long, with wide base and acute top; humeral tubercle laterally weakly produced or absent; gaster partially shagreened; body bright brown to dark brown; most often head and mesosoma brighter than gaster; legs brownish-yellow. **Minor worker.** Occipital margin of head slightly straight to slightly concave; head in full-face view oval; posterior and anterior of eyes convex; scape, when laid back, exceeding the posterior head margin by one-fifth of its length; sculpture shiny; smooth, sometimes indistinctly shagreened on frons; promesonotum smooth; propodeum punctate, sometimes with a few additional rugae; gaster smooth and shiny; body yellow to brown, head and gaster sometimes darker.

**Biology.** The species was collected between 1–1575 m in elevation. Sampling sites were predominantly located in anthropogenic and urban areas such as roadsides, coastal scrublands, and urban gardens. There are also a number of records from natural habitats, *e.g.*, rainforest (Madagascar, Mauritius, Mayotte and Reunion), tropical dry forest (Reunion and Mayotte), littoral rainforest (Madagascar), Ficus forest (Seychelles), mangrove (Mayotte) or palm forest (Seychelles). Nests were located in rotten logs and sticks on and above the ground, in rotting tree stumps, under stones and tree bark, and in soil.

**Comments.** *Pheidole megacephala* is globally distributed and recognized as one of the most aggressive and destructive invasive species. The species was described from Mauritius. However, its origin is still unclear and disputed (Sarnat et al., 2015). Due to this uncertainty, we treat it here as presumably native to the Malagasy region. In the Malagasy region, *P. megacephala* has been collected in both natural and anthropogenic habitats. On Madagascar, studies are needed to evaluate the behavior and invasive status in both native and human modified landscapes.

Three members of the *megacephala* group are known from Malagasy islands: *P. megacephala*, *P. megatron,* and *P. spinosa*. *Pheidole megacephala* is the only member of this group known from Mauritius (where specimens were collected from rainforest, closed vegetation, and coastal scrub), and Reunion (where specimens were collected from tropical dry forest, rainforest, and urban areas).

*Pheidole megacephala* co-occurs with *P. megatron* parapatrically on Comoros (Anjouan) and sympatrically in two urban areas of Madagascar (Ambilobe in Antsiranana and Mahanoro in Toamasina). Majors of *P. megacephala* can be separated from *P. megatron* based on predominantly smooth and only partially shagreened occipital lobes, indistinctly and sometimes not entirely shagreened first gastral tergite, and cordate and widening posteriorly head. In contrast, majors of *P. megaron* have entirely to predominantly indistinctly to distinctly shagreened occipital lobes, distinctly shagreened gaster, and sub-rectangular head with slightly convex lateral sides.

*Pheidole megacephala* is sympatric with *P. spinosa* on Comoros (Anjouan), Mayotte (coastal scrubs of Tanaraki and Dapani), and in urban areas of Madagascar (Antsirabe and Ankazobe in Antananarivo, Ambilobe in Antsiranana, Ranohira and Ambositra in Fianarantsoa, Moramanga in Toamasina, and Ampanihy in Toliara). Both species also co-occur in the rainforests of Forêt Classée Vatovavy in Fianarantsoa (175 m alt.), Region Atsinanana (48 m alt.), Réserve Naturelle Betampona (500–550 m alt.), and Tampolo (10 m alt.) in Toamasina. The two species are parapatric on Seychelles (Silhouette Island). *Pheidole megacephala* can be easily separated from regular majors of *P. spinosa* based on partially shagreened gaster and occipital lobes, and cordate shape of the head. Separation from supermajors of *P. spinosa* can be accomplished based on the presence of partially (sometimes indistinctly) sculptured occipital lobe, the minute propodeal spines with the wide base, and the absent to weakly developed humeral tubercle. In contrast, supermajors of *P. spinosa* have smooth occipital lobe, thin and acute propodeal spines, and well-developed humeral tubercles.

**Table:** 

**Figure 8:**
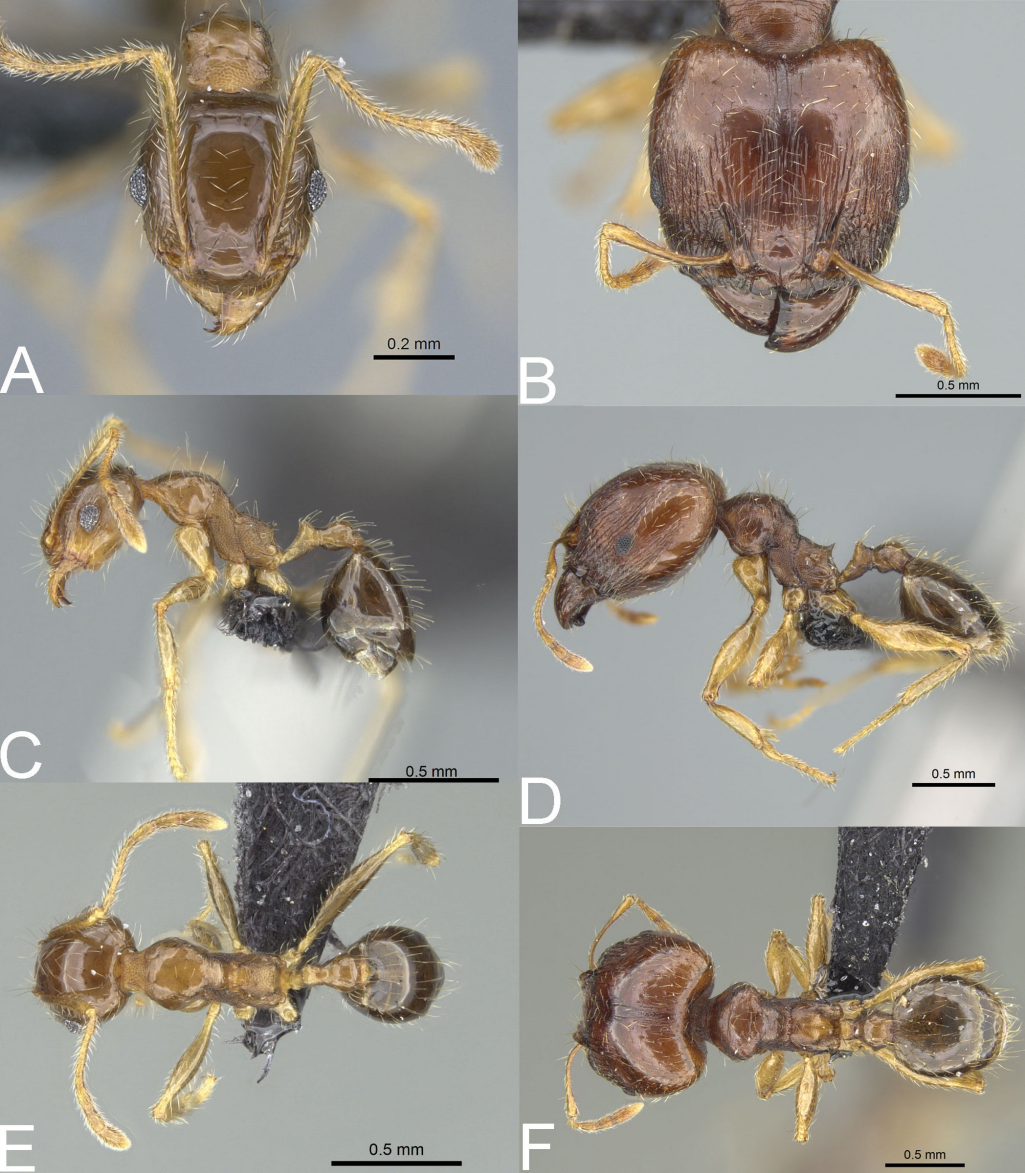
*Pheidole megatron*. Full-face view (A), profile (C), and dorsal view (E) of minor worker and full-face view (B), profile (D), and dorsal view (F) of major worker. Photo credit: Sebastian Salata.

**Figure 9:**
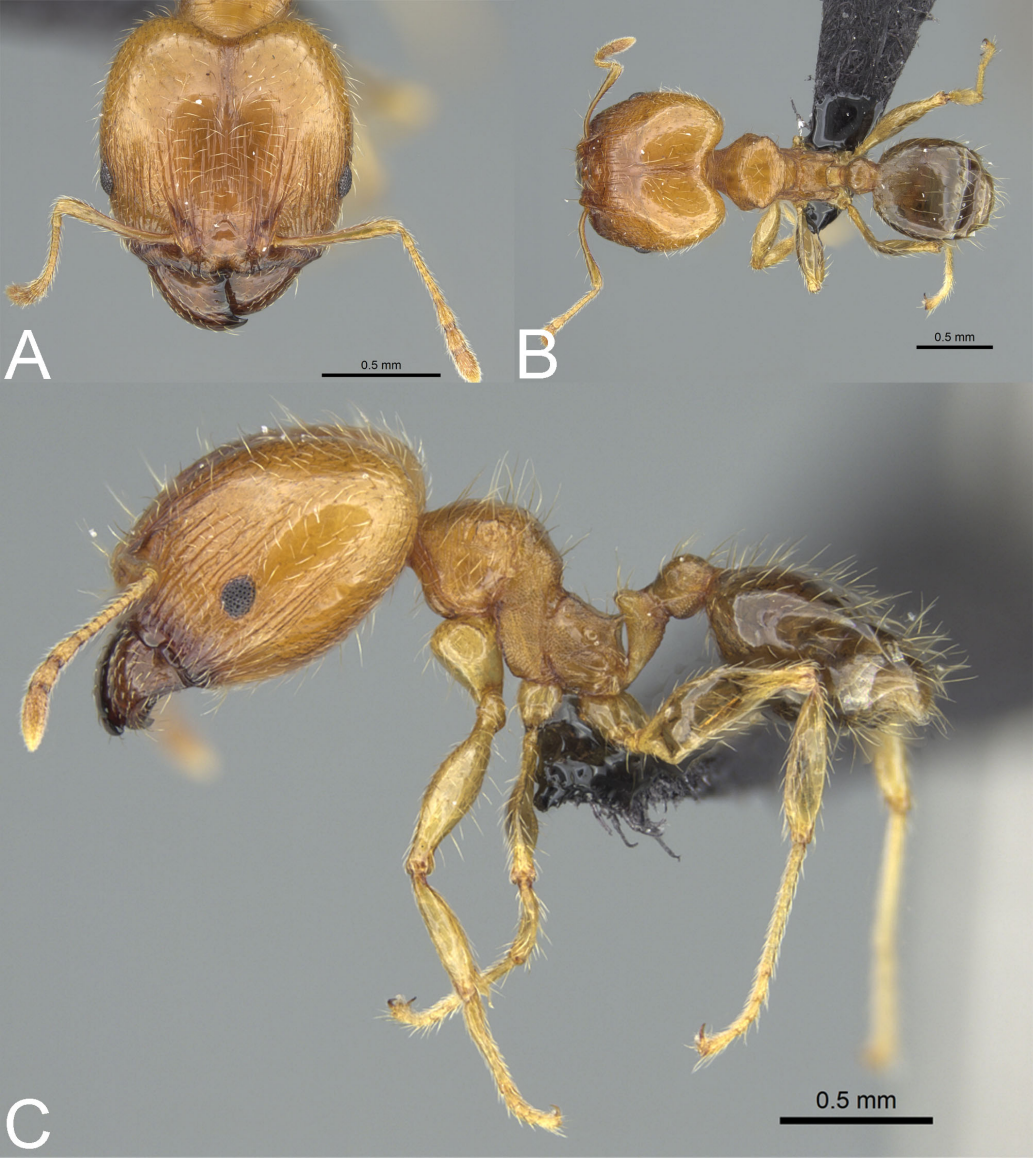
*Pheidole megatron*. Major worker with unusually bright body colouration. Full-face view (A), dorsal view (B), profile (C). Photo credit: Sebastian Salata.

***Pheidole megatron*Fischer & Fisher, 2013**
Figs. 5B, 6B, 6F, 6J, 8A–8F, 9A–9C
*Pheidole megatron*Fischer & Fisher, 2013: 338

**Type material.** Holotype: s., Comoros, Mohéli, Lac Boundouni, 12.3792 S/43.8517 E, 25 m, dry forest, under stone, 20.i.2009, coll. B.L. Fisher et al., BLF20771, CASENT0147194 (CASC) [personally investigated]. Paratypes: 2w., same data as holotype, CASENT0147193, CASENT0059654 (CASC) [personally investigated]; 2w., 1s., COMOROS, Mohéli, Lac Boundouni, 12.3792 S/43.8517 E, 25 m, dry forest, ex rotten log, 20.i.2009, coll. B.L. Fisher et al., BLF20797, CASENT0147140, CASENT0147141 (CASC) [personally investigated]; 3w., 1s., Mohéli, Lac Boundouni, 12.3792 S/43.8517 E, 25 m, dry forest, ex rotten log, 20.i.2009, coll. B.L. Fisher et al., BLF20762 & BLF20758, CASENT01281179, CASENT0147183, CASENT0147241 (CASC) [personally investigated].

**Material investigated.** See Table S1.

**Geographic range.** *Pheidole megatron* is a Malagasy endemic species, so far recorded from Comoros and the northeastern portion of Madagascar.

**Diagnosis. Major worker.** Head in full-face view sub-rectangular with slightly convex lateral sides; in lateral view sub-oval; margins of the head with dense, subdecumbent to suberect pilosity; antennal scrobe indistinct; occipital lobe shagreened; inner hypostomal tooth absent or indistinct; outer hypostomal tooth lobe-like; median tooth absent; propodeal spine short to moderately long, with wide base and acute top; humeral tubercle laterally weakly produced; gaster entirely shagreened; body brown to dark brown; most often head and mesosoma brighter than gaster; legs brownish-yellow. **Minor worker.** Occipital margin of head slightly straight to slightly concave; head in full-face view oval; posterior and anterior of eyes convex; scape, when laid back, exceeding the posterior head margin by one-fifth of its length; sculpture shiny; smooth, sometimes indistinctly shagreened on frons; promesonotum smooth; propodeum punctate, sometimes with a few additional rugae; gaster smooth and shiny; body yellow to brown, head and gaster sometimes darker.

**Biology.** The species was collected between 5–247 m in elevation. The sampling sites were located in anthropogenic and urban areas such as coconut plantations, cultivated land, disturbed forests, coastal scrublands, and urban gardens. There are also a number of records from natural habitats, *e.g.*, littoral rainforest, mangrove, and savannah woodland (Madagascar), and dry forest (Comoros). Nests were located in rotten logs and sticks above the ground, in rotting tree stumps, under stones and tree bark, and in soil.

**Comments.** *Pheidole megatron* is native and probably endemic to the Malagasy Region. However, the species is morphologically very similar to and easily confused with *P. megacephala*. Thus, we cannot exclude the possibility that some historical records of *P. megacephala* were in fact wrongly determined samples of *P. megatron*, making the latter species more widespread. Additionally, our study revealed that the body coloration of major workers of *P. megatron* varies more widely than indicated in the original description (Fischer & Fisher, 2013), and ranges from dark brown to yellow. However, majors with brighter coloration bear the same (but less visible) morphological characters as typical forms (Figs. 9A–9C).

*Pheidole megatron* is the only member of the *megacephala* group known from Grande Comore and Mohéli of Comoros and was collected there in both natural and disturbed habitats (coconut plantation, coastal scrub, and dry forest). On Anjouan, another island of Comoros, *P. megatron* co-occurs with *P. megacephala* and *P. spinosa.*

The species was also collected in some natural habitats in the northwestern portion of Madagascar. The majority of samples were collected in the Antsiranana prefecture: rainforest in Tafiambotry, Forêt d’Analabe (sympatric with *P. spinosa*) and Forêt Ambato (sympatric with *P. spinosa*), littoral rainforest in Antsaraingy, and disturbed rainforest in Ankobahoba. Additionally, there are records of *P. megatron* from Mahajanga prefecture from mangrove in Maropapango, and disturbed forests in Manerinerina and Ampamakiambato (in both localities *P. megatron* was sympatric with *P. spinosa*). On Madagascar, *P. megatron* was also collected from anthropogenic habitats located in Antsiranana, Mahajanga, and Toamasina prefectures.

In Antsiranana specimens of the species were collected on coconut plantation in Taizambato, sugar cane plantation in Andranomatàna, and urban areas of Sambava, Antalaha, and Antongombato. Additionally, *P. megatron* was sympatric with *P. spinosa* in urban areas of Vohemar, Ambanja, and Antsiranana, and sympatric with *P. megacephala* in Ambilobe. In Mahajanga prefecture, *P. megatron* is known from urban areas of Majunga, whereas in Toamasina prefecture, *P. megatron* is sympatric with *P. megacephala* in urban areas of Mahanoro.

In the Malagasy region *P. megatron* can be easily separated from majors and supermajors of *P. spinosa* by entirely to predominantly shagreened occipital lobes, at least first gastral tergite distinctly shagreened, short propodeal spines with wide base, and weakly developed humeral tubercles. Separation from *P. megacephala* should be conducted based on a series of several majors as the differences between these taxa are subtle. Majors of *P. megatron* differ from majors of *P. megacephala* in entirely to predominantly shagreened occipital lobes, at least first gastral tergite distinctly shagreened, and sub-rectangular head with slightly convex lateral sides. In contrast, majors of *P. megacephala* have occipital lobes predominantly smooth and only partially shagreened, first gastral tergite is less distinctly shagreened and sometimes partially smooth, and their head is cordate and widening posteriorly.

**Table:** 

**Figure 10:**
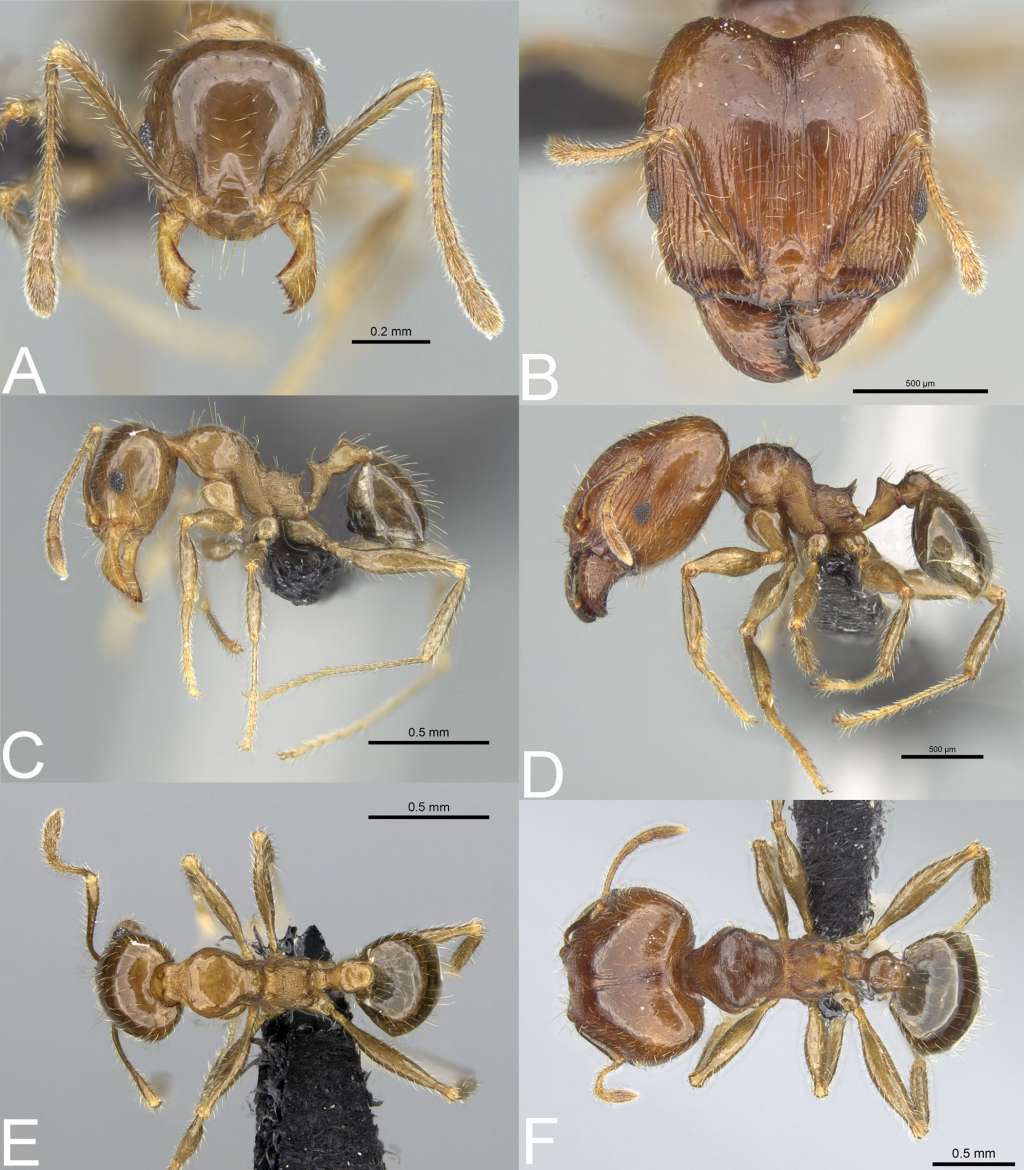
*Pheidole spinosa*. Full-face view (A), profile (C), and dorsal view (E) of minor worker and full-face view (B), profile (D), and dorsal view (F) of major worker. Photo credit: Sebastian Salata.

**Figure 11:**
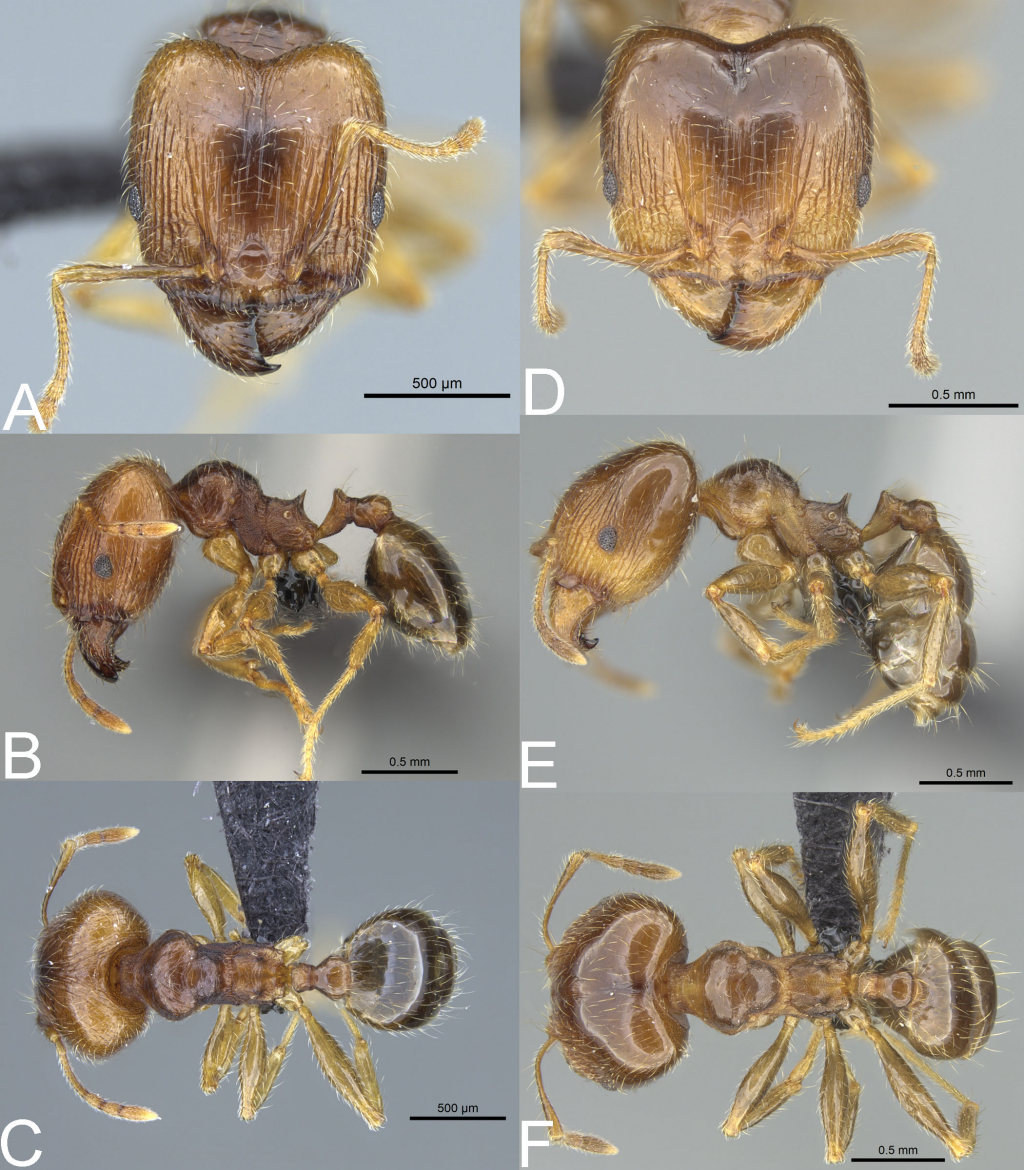
*Pheidole spinosa*. Major with well-developed sculpture, full-face view (A), profile (B), and dorsal view (C). Major with darker body colouration and shallow posterior emargination, full-face view (D). Profile (E), and dorsal view (F). Photo credit: Sebastian Salata.

**Figure 12:**
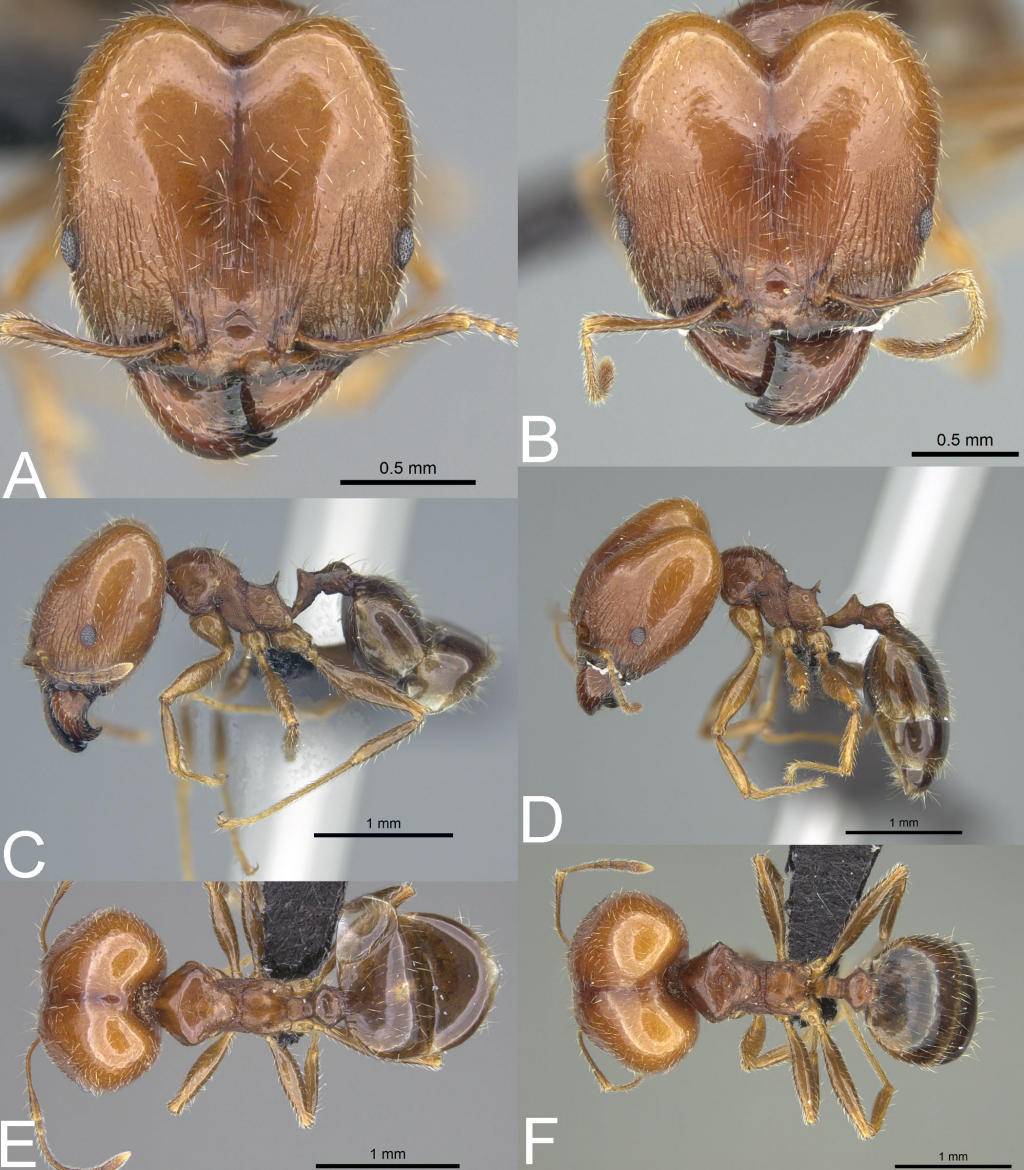
*Pheidole spinosa* nest sample (BLF04963). Major worker, full-face view (A) profile (C) and dorsal view (E) Supermajor worker, full-face view (B) profile (D) and dorsal view (F) Photo credit: Sebastian Salata.

***Pheidole spinosa*Forel, 1891 stat. nov.**
Figs. 5C–5D, 6C–6D, 6G–6H, 6K–6L, 10A–10F, 11A–11F, 12A–12F
*Pheidole megacephala* var. *spinosa*Forel, 1891: 178 (s.w.)
Subspecies of *Pheidole punctulata* Mayr, 1866: Forel, 1905: 164; Forel, 1907: 81.
Subspecies of *Pheidole megacephala* (Fabricius, 1793): Forel, 1897: 186; Wheeler, 1922: 1019; Bolton, 1995: 330.
=*Pheidole megacephala scabrior*Forel, 1891**syn. nov.**

Type material: Lectotype [designated here]: 1s., MADAGASCAR, Nosibé, coll. Voeltzkow, CASENT0101560 (MHNG) [personally investigated]. Paralectotypes: 2w., MADAGASCAR, Nosibé, coll. Voeltzkow, CASENT0101580 (MHNG) [personally investigated].

=*Pheidole picata* Forel, 1891 **syn. nov.**

Type material: Lectotype [designated here]: 1s., MADAGASCAR, Antananarivo, coll. Camboué, CASENT0101971 (MHNG) [personally investigated]. Paralectotypes: 1s., MADAGASCAR, Antananarivo, coll. Camboué, CASENT0101767 (MHNG) [personally investigated]; 3w., MADAGASCAR, Antananarivo, coll. Camboué, CASENT0101755 (MHNG) [personally investigated].

=*Pheidole picata gietleni* Forel, 1905 **syn. nov.**

Type material: Lectotype [designated here]: 1s. (top specimen on the pin), MADAGASCAR, Fianarantsoa, coll. Gietlen, CASENT0103296 (MHNG) [personally investigated]. Paralectotypes: 1s. (bottom specimen on the pin), MADAGASCAR, Fianarantsoa, coll. Gietlen, CASENT0103297 (MHNG) [personally investigated]. 2w., MADAGASCAR, Fianarantsoa, coll. Gietlen, CASENT0103307, CASENT0103308 (MHNG) [personally investigated].

=*Pheidole picata bernhardae* Emery, 1915 **syn. nov.**

Type material: Lectotype [designated here]: 1s., MADAGASCAR, Fianarantsoa, coll. Gietlen, CASENT0101332 (MHNG) [personally investigated]. Paralectotypes: 2w., MADAGASCAR, Fianarantsoa, coll. Gietlen, CASENT0101333 (MHNG) [personally investigated].

=*Pheidole decepticon* Fischer & Fisher, 2013 **syn. nov.**

Type material: Holotype: s., Mayotte, Mont Chongui, 12.95945 S/45.1341 E, 380 m, rainforest, ex rotten log, 28.xi.2007, coll. B.L. Fisher et al., BLF18916, CASENT0132558 (CASC) [personally investigated]. Paratypes: 1w., Mont Chongui, 12.95945 S/45.1341 E, 380 m, rainforest, on low vegetation, 28.xi.2007, coll. B.L. Fisher et al., BLF18897, CASENT0132583 (CASC) [personally investigated]; 1w., Mont Chongui, 12.996 S/45.1343 E, 550 m, rainforest, sifted leaf litter, 28.xi.2007, coll. B.L. Fisher et al., BLF18860, CASENT0125677 (CASC) [personally investigated].

**Type material.** Lectotype [designated here], 1s. (top specimen), MADAGASCAR, Antananarivo, coll. Camboué, CASENT0101330 (MHNG) [personally investigated]. Paralectotypes: 2s., MADAGASCAR, Antananarivo, coll. Camboué, CASENT0876545, CASENT0101331 (MHNG) [personally investigated]; 2s., MADAGASCAR, Fianarantsoa, CASENT0101554 (MHNG) [personally investigated]; 2w., MADAGASCAR, Antananarivo, coll. Camboué, CASENT0101570 (MHNG) [personally investigated].

**Material investigated.** See Tables S1 and S4.

**Geographic range.** *Pheidole spinosa* is a Malagasy endemic species, so far recorded from Comoros, Juan de Nova Island, Madagascar, Mayotte, and Seychelles.

**Diagnosis. Major worker.** Head in full-face view sub-rectangular, not or slightly widening posteriorly; in lateral view sub-oval; margins of the head with dense, subdecumbent to suberect pilosity; antennal scrobe indistinct; sides posterolateral from eyes entirely smooth or partially microrugulate; occipital lobe smooth, in single specimens partially microrugulate; inner hypostomal tooth absent or indistinct; outer hypostomal tooth lobe-like; median tooth absent; propodeal spine thin, moderately long, with wide base and acute top; humeral tubercle laterally weakly produced, in supermajors developed and triangular; gaster shiny and smooth, only supermajors with gaster indistinctly shagreened; body yellow to brown; most often head brighter than mesosoma and gaster; legs and antenna yellow to brownish-yellow. **Minor worker.** Occipital margin of head slightly straight to slightly concave; head in full-face view oval; posterior and anterior of eyes convex; scape, when laid back, exceeding the posterior head margin by one-fifth of its length; sculpture shiny; smooth, sometimes indistinctly shagreened on frons; promesonotum smooth; propodeum punctate, sometimes with a few additional rugae; gaster smooth and shiny; body yellow to brown, head and gaster sometimes darker.

**Redescription. Major worker.** Measurements (*n* = 20): HL: 1.16–1.94 (1.56); HW: 1.11–1.98 (1.53); SL: 0.59–0.85 (0.76); EL: 0.15–0.22 (0.19); WL: 1.00–1.35 (1.19); PSL: 0.18–0.28 (0.23); MTL: 0.64–0.93 (0.8); PNW: 0.62–0.86 (0.73); PTW: 0.16–0.26 (0.21); PPW: 0.29–0.5 (0.39); CI: 91.8–102.3 (98.1); SI: 42.6–60.6 (50.5); PSLI: 12.4–16.1 (14.7); PPI: 44.4–67.1 (55.7); PNI: 43.3–55.5 (48.3); MTI: 47.1–57.6 (52.9).

**Head.** In full-face view sub-rectangular, not or slightly widening posteriorly, with anterior and posterior margins slightly convex; posterior emargination shallow to relatively deep (Fig. 10B). In lateral view sub-oval. Inner hypostomal tooth not visible. Margins of the head with dense, subdecumbent to suberect pilosity; head dorsum with dense, long, and suberect to erect pilosity. Antennal scrobe indistinct; microrugulate with additional sparse and thick costulae. Frons with thick and sparse costulae; interspaces between costulae mostly smooth or indistinctly microrugulate. Sides posterolateral from eyes entirely smooth or partially microrugulate; occipital lobe smooth, in single specimens partially microrugulate. Gena with sparse and thick costulae; interspaces between costulae distinctly microreticulate. Centre of clypeus smooth and shiny; lateral margins with few costulae; median notch present, wide, and shallow; median longitudinal carina present, sometimes indistinct; lateral longitudinal carinae present. Scape, when laid back, exceeding the midlength of the head by one-fifth of its length, in supermajors not reaching the midlength of the head; pilosity subdecumbent to suberect (Figs. 10B, 10D). Inner hypostomal tooth absent or indistinct; outer hypostomal tooth lobe-like; median tooth absent (Fig. 4C). **Mesosoma.** In lateral view, mesonotal process absent; promesonotum strongly convex, well above the level of propodeum; posterior mesonotum smoothly declining towards propodeum; promesonotal groove absent; metanotal groove indistinct; propodeal spine thin, moderately long, with wide base and acute top; humeral tubercle laterally weakly produced, in supermajors developed and triangular (Fig. 10D). Surface shiny; promesonotal dorsum with spare rugulae and smooth to partially microrugulate interspaces; lateral sides of pronotum with microrugulae, sometimes with additional rugae. Pilosity dense, long, and erect (Figs. 10D, 10F). **Petiole.** Shiny and microrugulate; node in rear view dorsoventrally convex; petiolar peduncle long, with indistinct to small horizontal lobe on its basal part (Figs. 10D, 10F). **Postpetiole.** Shiny and smooth to microrugulate; dorsum with reduced sculpture and sometimes with a smooth notch; in dorsal view oval (Figs. 10D, 10F). **Gaster.** Shiny and smooth, only supermajors with gaster indistinctly shagreened; pilosity dense, long, and erect (Figs. 10D, 10F). **Colour.** Yellow to brown; most often head brighter than mesosoma and gaster; legs and antennae yellow to brownish-yellow (Figs. 10D, 10F).

**Redescription. Minor worker.** Measurements (*n* = 20): HL: 0.55–0.77 (0.69); HW: 0.45–0.69 (0.62); SL: 0.62–0.83 (0.76); EL: 0.11–0.15 (0.13); WL: 0.69–0.93 (0.85); PSL: 0.07–0.15 (0.12); MTL: 0.47–0.68 (0.6); PNW: 0.3–0.45 (0.4); PTW: 0.08–0.13 (0.1); PPW: 0.13–0.21 (0.18); CI: 82.1–92.2 (88.7); SI: 109.1–137.3 (122.8); PSLI: 13.3–19.1 (16.8); PPI: 47.9–63.5 (55.7); PNI: 61.7–67.5 (65.3); MTI: 88.4–108.2 (98.1).

**Head.** Occipital margin slightly straight to slightly concave; occipital carina absent (Fig. 10A). Antennal socket shallow and surrounded by a few indistinct, thin, and curved outward rugae; frontal lobe absent; head in full-face view oval; posterior and anterior of eyes convex; scape, when laid back, exceeding the posterior head margin by one-fifth of its length, pilosity dense, subdecumbent to erect; clypeus smooth and shiny, its anterior margin regularly convex; clypeus with median longitudinal carina absent, two lateral longitudinal carinae absent. Pilosity dense, long, and decumbent to erect. Sculpture shiny, smooth, and sometimes indistinctly shagreened on frons (Figs. 10A, 10C). **Mesosoma.** In lateral view, promesonotum low, arched; promesonotal groove absent; metanotal groove indistinct; humeral tubercle not developed into projection; promesonotum well above the level of propodeum; posterior mesonotum smoothly declining towards propodeum; propodeal spine small and with a wide base (Fig. 10C). Promesonotum smooth; propodeum punctate, sometimes with a few additional rugae. Pilosity dense, long, and erect (Figs. 10C, 10E). **Petiole.** Petiolar peduncle with ventral face slightly convex, node triangular, with few short, erect setae. **Postpetiole.** Convex, with few short, erect setae; in dorsal view widening posteriorly (Figs. 10C, 10E). **Gaster.** Smooth and shiny; with sparse, erect pilosity (Figs. 10C, 10E). **Colour.** Yellow to brown, head and gaster sometimes darker (Figs. 10C, 10E).

**Biology.** The species was collected between 1–2,150 m in elevation. It is known from natural and synanthropic habitats, but the majority of records are from natural sites, such as deciduous dry forests, gallery forests, littoral rainforests, montane rainforests, savannah woodlands and grasslands, and spiny forests (Madagascar); rainforests (Madagascar and Mayotte); *Casuarina* forest (Juan de Nova Island); mangroves (Mayotte); and native wet forests (Seychelles). The species was also recorded from urban or anthropogenic sites such as roadsides and paths (Madagascar, Juan de Nova Island, Comoros), coffee and eucalyptus plantations (Madagascar), and human settlements (Juan de Novo Island and Madagascar). Nests were located in rotten branches, logs and sticks on and above the ground, in rotting tree stumps, under stones, rootmats, moss, tree bark, and in soil. There are also records of nests located in carton nests, rot pockets, and termite mounds.

**Comments.** Five taxa described from Madagascar are associated with the *megacephala* group. Forel (1891) described three varieties of *P. megacephala*: *P. megacephala spinosa*, *P. megacephala picata* and *P. megacephala scabrior*. In the original description, *P. megacephala spinosa* was separated from other subspecies based on majors with yellowish-red body coloration, longer propodeal spines, and rather elongated head with lateral sides only slightly convex. Later, Emery (1915) confirmed its separateness and *P. spinosa* maintained its status as a subspecies of *P. megacephala* until modern times.

*Pheidole megacephala picata* was recognized based on smaller body size, darker body colouration, more convex head capsule, and rather short propodeal spines. Its type specimens were collected at the sampling locality as types of *P. spinosa* but Forel concluded that the observed morphological differences supported the distinctiveness of both subspecies. Later, Forel (1905), based on studies of sexuals, considered this form a subspecies of *P. punctulata*, and finally *P. picata* was elevated to the species level (Emery, 1915). However, recently Fischer & Fisher (2013) recognized this species as a junior synonym of *P. megacephala*.

Finally, *P. megacephala scabrior* was described based on stronger and denser sculpture on posterior head and mesosoma. Later, the species was also recorded from Seychelles (Emery, 1894; Donisthorpe, 1932) and Emery (1915) confirmed its distinctiveness. *Pheidole m. scabrior* maintained its status as a subspecies of *P. megacephala* until 2013, when Fischer & Fisher (2013) recognized it as its junior synonym.

The two remaining names, *i.e., P. punctulata gietleni* and *P. punctulata spinosa bernhardae*, were described in 1905 by Forel (1905)—the latter was later validated as trinominal by Emery (1915). *Pheidole punctulata gietleni* was separated based on relatively small and subrectangular head with very shallow posterior emargination, relatively short propodeal spines, head with smooth posterior half and yellowish-red to yellowish-brown body colouration. This taxon was later considered a subspecies of *P. picata* until the recent work of Fischer & Fisher (2013), who recognized it as a junior synonym of *P. megacephala*. By contrast, the *bernhardae* form was separated based on larger body size, square head shape, long propodeal spines, and almost blackish body colouration. This form was later considered a subspecies of *P. picata* (Emery, 1915) and finally recognized as a junior synonym of *P. megacephala* (Fischer & Fisher, 2013).

The inventory of Madagascan samples of *P. spinosa* revealed that this species manifests very high intraspecific variability. Our preliminary results allowed us to recognize morphotypes matching all taxa of the *megacephala* group described from the island. However, no stable and sound morphological feature separates these forms. We could locate specimens suiting morphologically different names even within a single colony. In general, samples of *P. spinosa* collected in the northern part of Madagascar and in littoral rainforests are usually smaller and their morphology resembles that associated with *P. gietleni* and *P. picata* (Figs. 11D–11F). Samples collected from central highlands and open or dry habitats have brighter body coloration, are larger, and look similar to *P. spinosa sensu lato* and *P. bernhardae* (Figs. 10A–10F). Within large nest samples of *P. spinosa/bernhardae* morphotypes, we were usually able to locate single specimens with morphology matching *P. scabrior* (Figs. 11A–11B). Based on this data we recognize all of these names as junior synonyms of *P. spinosa*. Despite differences in the body colouration and overall body shape, all of these forms have subrectangular head capsule, reduced sculpture on head, more developed humeral peduncles, and smooth gaster. These characters are unique for this species and clearly separate it from *P. megacephala* and *P. megatron*. We decided to give priority to *P. spinosa* over *P. picata* and *P. scabrior* because this name is listed as the first one by Forel (1891) and represents the most common morphotype known from the island.

We also agree with Sarnat et al. (2015) and recognize *P. decepticon* as a junior synonym of *P. spinosa*. Studies on types of *P. decepticon* revealed that there is no stable morphological feature allowing separation of this species from *P. spinosa.* Bright body coloration, subrectangular head capsule, thin propodeal spines, reduced head sculpture, and smooth gaster cluster this species with the morphotype associated with *P. spinosa sensu lato*. Additionally, our conclusion is supported with results obtained from COI data. Thus, it appears that *P. spinosa* is the most common and dominant member of the *megacephala* group in the Malagasy region. We also agree with Fischer & Fisher (2013) and assign literature records of *P. punctulata* (Forel, 1907; Forel, 1897) from Madagascar and Seychelles to *P. spinosa*.

*Pheidole spinosa* is also the only Malagasy *Pheidole* species with a confirmed tendency to produce supermajors. This tendency was previously recorded in some Afrotropical members of the *megacephala* group (Sarnat et al., 2015) and we confirm that the southernmost Madagascan populations of *P. spinosa*, collected mostly from dry or open habitats, express a tendency to develop a third caste of workers. As shown in Fig. 12, some colonies of *P. spinosa* produce supermajors morphologically distinct from typical major workers. Their body is larger, propodeal spines longer and thinner, head more cordate, and gaster is shagreened. Unfortunately, it is unclear which factors stimulate the development of this additional caste in Madagascan populations of *P. spinosa*.

*Pheidole spinosa* is the only member of the *megacephala* group recorded from Juan de Nova Island and its specimens were collected there in natural and anthropogenic sites (*Casuarina* forest, scrub on coastal karst, littoral vegetation, old settlement). *Pheidole spinosa* co-occurs with *P. megacephala* on Anjouan in Comoros, Madagascar, Mayotte, and Silhouette Island in Seychelles, whereas *P. megatron* co-occurs on Anjouan in Comoros and Madagascar. Majors of *P. spinosa* can be easily separated from *P. megacephala* and *P. megatron* based on smooth gaster, thinner and longer propodeal spines, and sub-rectangular head capsule which only indistinctly widens posteriorly if at all. Supermajors of *P. spinosa* can be separated from *P. megacephala* and *P. megatron* based on smooth occipital lobe, thin and acute propodeal spines with narrow base, and well-developed humeral tubercle.

In fact, *P. spinosa* is morphologically closer to some Afrotropical members of the *megacephala* group than to its Malagasy congeners. *Pheidole spinosa* differs from *P. punctulata* and its subspecies in smooth occipital lobes and gaster and never suboval head capsule; and from *P. megacephala melancholica, P. m. atrox, P. m. speculifrons, P. m. impressifrons, P. m. costauriensis* and *P. m. duplex* in smooth occipital lobes and gaster, thinner propodeal spines, and more elongated and less convex head in lateral view. *Pheidole spinosa* is the most similar to *P. m. rotundata* and differs in never suboval head shape in frontal view, entirely smooth occipital lobes, and entirely smooth gaster lacking even indistinct sculpture.

## Discussion

Morphologically, *P. megacephala* and *P. megatron* share a set of characters so far not recorded for other members of the *megacephala* group. Their majors are on average smaller than *P. spinosa* and the Afrotropical members of the *megacephala* group, and they have a less developed humeral area. Intraspecific variation in the head shape and body sculpture within colonies of *P. megacephala* and *P. megatron* appears to be very small. However, the most striking difference between these two species and remaining representatives of the *megacephala* group is the lack of ability to produce supermajors.

Emery (1915), while studying *P. punctulata* and *P. m. rotundata*, noticed that both species can be easily distinguished only when the extreme forms of majors are used for separation. However, when he included specimens of various body sizes, the differences between species faded. He concluded that limited material from the Afrotropics prevented him from verifying the validity of already described species of the *megacephala* group and suggested that they could be just variations of single, morphologically variable species. Sarnat et al. (2015) reached the same conclusions and for the first time introduced the term “supermajors” for the biggest, morphologically distinct majors of the species of the *megacephala* group. Our observations on the Afrotropical samples also confirm that some, if not all, members of the *megacephala* group from this region have a tendency to produce the third, morphologically distinct caste. However, due to limited material, it is unclear if supermajors are present in all colonies and which factors trigger their presence in nests.

The taxonomic history of *P. spinosa* validates almost all conclusions noted by Emery (1915) and Sarnat et al. (2015). Extremely high intraspecific variability of the species resulted in descriptions of its various phenotypes as separate taxa. Most often, these descriptions were based on very limited material that consisted of morphologically distinct forms of major workers. Only the study of vast quantities of material recently collected from across the range of distribution of this species revealed that when more samples are considered, it is impossible to define species limits between these forms. What triggers such high intraspecific variability in *P. spinosa* is still unknown. Maybe, as suggested by Fischer & Fisher (2013), the main reason is an ongoing differentiation between populations of this species.

Based on collected material, *P. spinosa* is also the only Malagasy member of the group with a tendency to produce supermajors. This species appears to be morphologically closer to some Afrotropical species than to its Malagasy congeners. The presence of supermajors could suggest that *P. spinosa* is more closely related to Afrotropical species, but this hypothesis requires further studies for validation. It is also not clear whether the existence of supermajors is limited only to the southernmost Madagascan populations of this species. Further studies on this subject should reveal whether observed limits in the distribution of this caste are the result of sampling bias or environmental factors.

##  Supplemental Information

10.7717/peerj.13263/supp-1 Supplemental Information 1 Distribution dataset Distribution records of specimens of the *Pheidole megacephala* species-group from Malagasy

10.7717/peerj.13263/supp-2 Supplemental Information 2 GenBank accession numbers for sequenced samples

10.7717/peerj.13263/supp-3 Supplemental Information 3 Nucleotide sequences of the 95 sequenced samples

10.7717/peerj.13263/supp-4 Supplemental Information 4 Morphometric dataset Morphometric characters of worker individuals of *Pheidole spinosa*. Data are given in mm

## Additional Information and Declarations

### Field Study Permissions

The following information was supplied relating to field study approvals (i.e., approving body and any reference numbers): Ant samples used in this study comply with the regulations for export and exchange of research samples outlined in the Convention on Biological Diversity and the Convention on International Trade in Endangered Species of Wild Fauna and Flora. For fieldwork conducted in Madagascar, Comoros, Juan de Nova Island, Mauritius, Mayotte, Reunion and Seychelles, permits to research, collect, and export ants were obtained from the Ministry of Environment and Forest as part of an ongoing collaboration between the California Academy of Sciences and the Ministry of Environment and Forest, Madagascar National Parks, and Parc Botanique et Zoologique de Tsimbazaza (Madagascar), Centre National de Documentation et de Recherche Scientifique (Comoros), Terres Australes et Antarctiques Françaises and Direction de la Conservation du Patrimoine Naturel (Juan de Nova Island), Forestry Service and National Parks and Conservation Service (Mauritius), Naturalistes de Mayotte (Mayotte), Insectarium de la Reunion (Reunion), and Seychelles Bureau of Standards and National Park Authority (Seychelles). Authorization for export was provided by the Director of Natural Resources.

### Data Availability

The following information was supplied regarding data availability: The raw data, including GenBank accession numbers, are available in the Supplemental Files and at Genbank: OM704654, OM704664, OM704667, OM704677, OM704652, OM704655, OM704659–OM704658. The material is deposited in the ant collection of the California Academy of Sciences, U.S.A. For AntWeb records that include images, please append the CASENT number available in Table S1 at the end of this URL: https://www.antweb.org/specimen/.
