# Supplementary material for: Taxonomic revision of the Pheidole megacephala species-group (Hymenoptera, Formicidae) from the Malagasy Region
Source: PeerJ. 2022 Apr 26;10:e13263. doi: 10.7717/peerj.13263 (PMC9053301; doi:10.7717/peerj.13263)
Supplement: Supplemental Information 3 [file peerj-10-13263-s003.rtf]

>CASENT0196027_Strumigenys_abderaNNNNNNNNNNNNNNNNNNNNNNNNNNNATCNGGAATAGTAGGTTCTTCTATAAGAATAATTATTCGTTTAGAATTAGGAACATGTAACTCATTAATTAATAATGATCAAATTTACAATTCTTTAATTACTAGTCATGCTCTAATTATAATTTTTTTTATAATTATACCATTTATAATTGGAGGATTTGGAAATTTTTTAGTGCCATTAATACTCGGATCTCCTGATATGGCTTATCCTCGATTAAATAATATAAGTTTTTGATTACTCCCCCCTTCTCTTATACTATTAATAAGTGGTAGATTTATCAGTGAAGGGGTTGGAACAGGTTGAACCATCTACCCACCCTTATCTTCAAATATTTTTCATAATGGTCCTTCAATTGACCTCTCAATTTTCTCACTTCATATTGCAGGAATATCCTCTATTTTAGGAGCAATCAATTTTATCGCAACTATTATAAATATACATAATACTAATTTAACATTAGATAAAATATCATTATTAGTTTGATCTATTAATATTACTGCTATTTTATTACTCCTTTCTCTACCTGTATTAGCTGGTGCAATTACCATATTATTTACAGATCGAAATTTAAATACTTCTTTTTTTGATCCTTCAGGAGGAGGAGATCCAATTTTATTTCAACATTTATTTNNNNNNNNNNNNNNN>CASENT0151993_Pilotrochus_besmerusTATTTTATATTTTATATTAGCTATTTGATCTGGTATAATTGGTTCTTCTATAAGAATAATTATTCGATTAGAATTAGGATCATGTAATTCATTAATTAATAATGATCAAATTTATAATATCTTAATTACAAATCATGCTTCAATTATAATTTTTTTTATAATTATACCTTTCATAATTGGAGGATTTGGAAATTTTTTAATTCCTCTAATATTAGGATCTCCCGATATAGCATTTCCTCGAATAAATTATATAAGATTTTGATTATTACCTCCCTCTATAATTCTTTTATTATTAAGAAATTTTATTAATGAAGGAACAGGAACAGGTTGAACATTATATCCTCCTTTATCTTCAAATATTTTTCACAGAGGAGCTTCAATTGATTTATCTATTTTTTCTTTACACATTGCTGGAATATCATCTATTTTAGGAGCTATTAACTTTATTACAACTATTATAAATATATATAATAAAAATATATCTTTAGACAAAATTCCTTTACTTATTTGATCTATTTTAATCACAACAATTTTACTTCTTCTTTCTTTACCCGTATTAGCTGGTGCTATTACCATATTACTTACAGATCGAAATTTAAATACATCTTTTTTTGATCCCTCCGGAGGAGGTGACCCTATTTTATTTCAACATTTATTTNNNNNNNNNNNNNNN>CASENT0738816_Pheidole_komoriNNNNNNNNNNNNNNNNNNNNNNNNNNNNNNNNNNNNNNNNNNNNNNNNNNNNNNNNNNNNNNNNNNNNNNNNNNNNGGATCATGTAATTCTCTAATTAATAATGATCAAATTTATAATTCTCTAGTAACAAGCCACGCTTTTATTATAATTTTTTTTATGGTTATACCTTTTATGATTGGAGGATTTGGAAATTTTTTAGTACCTCTAATATTAGGATCTCCAGATATAGCTTACCCTCGTATAAATAATATAAGATTTTGACTTCTACCTCCCTCCCTCACCCTCCTCCTTCTAAGAAGTTTCATTAATTCAGGAGTAGGAACTGGCTGAACAATTTACCCCCCTTTAGCCTCTAATATTTTCCATAGAGGAGCCTCAATCGATCTATCTATTTTTTCTTTACATATTGCAGGTGCCTCTTCTATTTTAGGAGCCATCAATTTTATTTCTACAATTATCAATATACATCATAAAAGCTTTTCAATAGATAAAATCCCACTATTAGTATGATCTATTCTAATCACCGCTATCCTTCTCCTTCTTTCTCTGCCTGTTCTTGCCGGAGCCATTACAATACTTCTTACTGACCGTAATCTCAATACTTCCTTTTTTGACCCCGCTGGCGGAGGAGATCCAATTCTTTACCAACATTTATTCTGATTTTTTGGTCAC> CASENT0114369_Pheidole_indicaTTTACTATACTTTATTTTTGCTATTTGAGCCGGGATAATTGGATCTTCTATAAGAATAATCATTCGGCTAGAATTAGGATCTTGCAACTCCTTAATCAATAATGATCAAATTTACAATTCCTTAGTTACAAGACACGCTTTTATTATAATCTTCTTTATAGTTATACCTTTCATAATTGGGGGATTCGGAAATTTTTTAATTCCTCTAATACTTGGATCCCCTGATATAGCTTACCCTCGTATAAATAATATAAGATTCTGACTCTTACCCCCCTCTATCACTCTTCTTATACTTGGAAGATTCATTAATTCTGGAGTTGGAACAGGATGAACAATTTATCCCCCCCTAGCCTCTAACATCTTTCATAGAGGAGCCTCTATCGATCTTTCTATTTTTTCTCTACACATCGCAGGCATATCATCTATCCTTGGAGCTATTAATTTTATTTCTACTATTATTAACATGCATCATAATAATTTTTCTATAGATAAAATTCCTTTATTAGTTTGATCCATTTTAATTACCGCTATTCTACTTCTTCTTTCACTCCCAGTTCTTGCAGGAGCTATTACTATACTCCTTACCGACCGAAATCTAAATACCTCTTTCTTTGACCCCGCTGGTGGAGGAGACCCTATTCTTTACCAACACCTATTTNNNNNNNNNNNNNNN>CASENT0161023_Pheidole_parvaAATTTTATATTTTATTTTCGCAATTTGAGCCGGAATAATTGGTTCTTCTATAAGAATGATTATTCGATTAGAACTGGGGTCTTGTAATGCATTAATTAACAATGATCAAATTTACAACTCTTTAGTTACAAGACATGCTTTTATTATAATTTTTTTTATAGTTATACCTTTTATAATTGGCGGATTTGGAAATTTTTTAGTACCTTTAATATTAGGGTCCCCAGATATAGCCTACCCACGTATAAACAATATAAGATTTTGATTACTACCCCCTTCTATTACACTTCTTCTTTTAGGGAGATTTATTAATTCCGGAGTTGGAACCGGATGAACAATCTATCCTCCTCTAGCCTCTAATATTTTTCATAGAGGAGCCTCAATTGATCTTTCTATTTTTTCTCTTCATATTGCAGGTATATCATCTATTTTAGGAGCTATTAATTTTATCTCTACAATTATTAATATACATCATAAAAATTTTACCATAGATAAAATTCCCCTTTTAGTTTGATCTATTTTAATCACCGCTGTGTTACTTCTCCTTTCTCTCCCAGTTCTTGCCGGGGCTATTACAATATTACTAACAGACCGAAATTTAAATACTTCTTTTTTCGATCCTGCTGGAGGAGGAGACCCTATCCTCTATCAACATCTATTTNNNNNNNNNNNNNNN>CASENT0196901_Pheidole_longispinosaTATATTATATTTTATTTTCGCTATTTGAGCTGGAATAATTGGATCTTCAATAAGAATAATTATTCGATTAGAGCTTGGATCATGTAATTCTTTAATTAATAATGATCAAATTTACAACTCTTTAGTTACAAGTCATGCTTTTATTATAATTTTTTTTATAGTAATGCCTTTTATAATTGGAGGATTTGGAAATTTTTTAGTACCTTTAATATTAGGATCTCCTGATATAGCATACCCTCGTATAAATAACATAAGATTTTGACTTCTACCACCTTCTATCACTCTTCTTCTTTTAGGAAGATTTATTAATTCAGGAGTAGGAACAGGATGAACTGTTTACCCCCCATTAGCTTCTAATATTTTTCATAGAGGAGCTTCAATTGATTTATCTATTTTTTCTTTACATATTGCTGGTATATCATCTATTTTAGGAGCTATTAATTTTATCTCTACAATTATTAATATACATCATAAAAATTTTACTATAGATAAAATTCCTTTATTAGTATGATCTATTATAATTACTGCAGTTTTATTACTTTTATCTTTACCTGTCCTNNNNNNNNNNNNNNNNNNNNNNNNNNNNNNNNNNNNNNNNNNNNNNNNNNNNNNNNNNNNNNNNNNNNNNNNNNNNNNNNNNNNNNNNNNNNNNNNNNNNNNNNNNNNNNNNNNN>CASENT0163051_Pheidole_oswaldiGATATTATATTTTATTTTTGCAATTTGAGCAGGAATAATTGGATCTTCTATAAGAATAATTATTCGTCTTGAACTTGGCTCTTGCAATTCATTAATTAATAATGATCAAATTTATAACTCTCTAGTTACAAGACACGCTTTTATTATAATTTTTTTTATAGTTATACCATTTATAATTGGAGGATTTGGAAATTTTTTAGTTCCTCTAATATTAGGATCTCCTGATATAGCTTACCCTCGAATAAATAATATAAGGTTTTGATTACTACCCCCCTCTATCACCCTCCTCCTTCTAGGAAGATTTATTAACTCAGGAGTCGGAACAGGATGAACTATCTACCCCCCCTTAGCATCTAATATTTTTCATAGAGGTGCCTCTATCGATCTATCAATTTTTTCTTTACATATTGCAGGAATATCATCTATTCTCGGAGCTATCAACTTTATTTCTACTATTATTAACATACATCATAAAAACTTCACCATAGATAAAATTCCACTCTTAGTTTGATCTATTATAATTACTGCTGTGCTATTACTCCTCTCCCTACCTGTACTAGCTGGTGCTATCACTATACTTTTAACCGATCGAAATTTAAATACTTCCTTTTTTGACCCATCTGGAGGAGGAGACCCTATTCTCTACCAACATTTATTTNNNNNNNNNNNNNNN>CASENT0047063_Pheidole_decollataAATATTATATTTTATTTTTGCAATTTGAGCAGGAATAATTGGGTCTTCTATAAGAATAATTATCCGTCTTGAACTTGGCTCTTGTAATTCACTAATTAATAATGATCAAATTTATAATTCCTTAGTTACAAGACATGCTTTTATTATAATTTTTTTTATAGTTATACCATTTATAATTGGAGGATTTGGAAATTTTTTAGTACCATTAATATTAGGATCTCCCGACATAGCCTACCCACGAATAAATAATATAAGATTCTGATTACTTCCACCATCTATCACTCTTCTTCTATTAGGAAGATTCATTAATTCAGGAGTAGGAACAGGATGAACTATTTATCCTCCATTAGCTTCTAATATTTTCCATAGAGGAGCCTCTATTGATCTTTCAATCTTTTCTTTACATATTGCCGGAATATCATCAATTCTAGGAGCTATTAATTTTATTTCTACTATCATTAATATACACCATAAAAATTTTACCATAGATAAAATCCCTCTTTTAGTGTGATCTATCATAATTACAGCTGTACTTTTACTTCTATCTCTACCAGTACTTGCCGGGGCTATCACCATACTTTTAACTGATCGAAATTTAAATACTTCCTTTTTTGACCCGTCTGGAGGGGGAGACCCTATTCTTTATCAACACTTATTTNNNNNNNNNNNNNNN>CASENT0135732_Pheidole_bessoniiNNNNNNNNNNNNNNNNNNNNNNNNNNNNNNNNNNNNNNNNNNNNNNNNNNNNNNNNTAATTATTCGTCTTGAACTTGGCTCATGTAATTCATTAATTAATAATGATCAAATTTATAACTCTTTAGTTACAAGACATGCTTTTATTATAATTTTTTTTATAGTTATACCATTCATAATTGGAGGATTCGGAAATTTTTTAGTTCCCCTAATACTCGGTTCACCTGATATAGCTTACCCTCGAATAAATAATATAAGATTTTGACTTCTTCCACCTTCTATTACCCTTCTTCTTTTAGGAAGTTTTATCAATTCCGGAGTTGGAACAGGTTGAACAGTCTACCCGCCTCTAGCTTCTAATATTTTCCATAGAGGTGCATCTATTGACCTATCTATTTTCTCTCTCCATATTGCTGGAATATCATCTATTTTAGGAGCTATTAATTTTATTTCTACAATCATTAATATACATCATAAAAATTTCACCATAGATAAAATTCCTCTTCTAGTTTGATCTATCATAATTACTGCTGTGTTATTACTTTTATCATTACCAGTTCTTGCTGGGGCTATTACTATACTTTTAACTGATCGAAATCTTAATACTTCATTCTTTGATCCTGCTGGTGGAGGAGATCCTATTCTTTACCAACACTTATTTNNNNNNNNNNNNNNN>CASENT0164251_Pheidole_mamirapiratraNNNNNNNNATTTTATTTTTGCCATTTGAGCAGGAATAATTGGATCATCCATAAGAATAATTATTCGTCTTGAACTTGGATCTTGTAACTCACTAATTAATAATGATCAAATTTATAACTCTTTAGTTACTAGTCATGCTTTTATTATAATTTTTTTTATAGTTATACCATTTATAATTGGGGGATTTGGAAATTTTTTAGTGCCTCTAATGCTTGGATCACCTGATATAGCCTACCCCCGAATAAATAATATAAGATTTTGACTTCTACCACCCTCTATTACTCTCCTTCTACTAGGGAGATTTATTAATTCTGGAGTTGGAACAGGATGAACCGTTTACCCTCCTTTAGCCTCTAATATTTTTCATAGAGGAGCTTCTATCGATTTATCAATTTTTTCCCTTCACATTGCTGGTATATCATCTATTTTAGGAGCTATTAATTTTATTTCAACTATTATTAATATACATCATAAAAATTTTACTATAGATAAAATCCCTCTTCTAGTCTGATCCATTATAATTACTGCCGTATTATTACTTCTTTCATTACCGGTACTAGCAGGAGCTATCACCATACTTTTAACTGATCGTAATCTTAATACATCATTCTTTGATCCTGCCGGAGGTGGAGACCCAATTCTTTACCAACATTTATTTNNNNNNNNNNNNNNN>CASENT0055882_Pheidole_megacephalaNATACTTTACTTTATCTTTGCGATCTGATCTGGGATAATTGGGTCCTCTATGAGATTAATTATTCGATTAGAACTCGGATCCTGTAATTCCTTAATTAATAATGATCAAATTTATAATTCATTAGTTACAAGACACGCTTTTATTATAATCTTCTTTATAGTTATACCTTTTATAATTGGAGGATTTGGTAATTTTTTAGTCCCTTTAATGCTAGGATCTCCAGATATAGCATACCCACGAATAAATAATATAAGGTTCTGGCTACTCCCTCCTTCTATCACTCTCCTTTTATTAGGAAGATTCATTAATTCAGGGGCCGGAACTGGGTGAACAGTCTATCCCCCCCTAGCTTCAAACATTTTTCACAGGGGAGCTTCTATCGATCTCTCAATTTTCTCATTACATATTGCAGGAATATCTTCCATTCTTGGAGCTATTAATTTTATCGCCACAATTATTAATATACATCATAAAAATTTTACTATAGATAAAATTCCCTTATTAGTTTGATCAATTTTAATTACAGCAATCCTTCTTCTTCTCTCCCTACCAGTCCTTGCTGGAGCAATTACTATACTCTTAACTGACCGTAATCTTAACACTTCCTTCTTTGACCCAGCAGGAGGAGNNGACCCCATTCTTTACCAACATCTATTTTNNNNNNNNNNNNNN>CASENT0059592_Pheidole_megacephalaNNNNNNNNNNNNNNNNNNNNNNNNNNNNNNNNNNNNNNNNNNGTCCTCTATGAGANTAATTATTCGATTAGAACTCGGATCCTGTAATTCCTTAATTAATAATGATCAAATTTATAATTCATTAGTTACAAGACACGCTTTTATTATAATCTTCTTTATAGTTATACCTTTTATAATTGGAGGATTTGGTAATTTTTTAGTCCCTTTAATGCTAGGATCTCCAGATATAGCATACCCACGAATAAATAATATAAGGTTCTGGCTACTCCCTCCTTCTATCACTCTCCTTTTATTAGGAAGATTCATTAATTCAGGGGCCGGAACTGGGTGAACAGTCTATCCCCCCCTAGCTTCAAACATTTTTCACAGGGGAGCTTCTATCGATCTCTCAATTTTCTCATTACATATTGCAGGAATATCTTCCATTCTTGGAGCTATTAATTTTATCGCCACAATTATTAATATACATCATAAAAATTTTACTATAGATAAAATTCCCTTATTAGTTTGATCAATTTTAATTACAGCAATCCTTCTTCTTCTCTCCCTACCAGTCCTTGCTGGAGCAATTACTATACTCTTAACTGACCGTAATCTTAACACTTCCTTCTTTGACCCAGCAGGAGGAGGGGANCCCATTCTTTACCAACATCTATTTNNNNNNNNNNNNNNN>CASENT0055945_Pheidole_megacephalaNNNNNNNNNNNNNNNNNNNNNNNNNNNNNNNNNNNNNNNNNGGTCCTCTATGAGANTAATTATTCGATTAGAACTCGGATCCTGTAATTCCTTAATTAATAATGATCAAATTTATAATTCATTAGTTACAAGACACGCTTTTATTATAATCTTCTTTATAGTTATACCTTTTATAATTGGAGGATTTGGTAATTTTTTAGTCCCTTTAATGCTAGGATCTCCAGATATAGCATACCCACGAATAAATAATATAAGGTTCTGGCTACTCCCTCCTTCTATCACTCTCCTTTTATTAGGAAGATTCATTAATTCAGGGGCCGGAACTGGGTGAACAGTCTATCCCCCCCTAGCTTCAAACATTTTTCACAGGGGAGCTTCTATCGATCTCTCAATTTTCTCATTACATATTGCAGGAATATCTTCCATTCTTGGAGCTATTAATTTTATCGCCACAATTATTAATATACATCATAAAAATTTTACTATAGATAAAATTCCCTTATTAGTTTGATCAATTTTAATTACAGCAATCCTTCTTCTTCTCTCCCTACCAGTCCTTGCTGGAGCAATTACTATACTCTTAACTGACCGTAATCTTAACACTTCCTTCTTTGACCCAGCAGGAGGAGGGGACCCCATTCTTTACCAACATCTATTTNNNNNNNNNNNNNNN>CASENT0060294_Pheidole_megacephalaNNNNNNNNNNNNNNNNNNNNNNNNNNNNNNNGGGATAATTGGGTCCTCTATGAGATTAATTATTCGACTAGAACTCGGTTCCTGTAATTCCTTAATTAATAATGATCAAATTTATAACTCATTAGTTACAAGACATGCTTTTATTATAATCTTCTTTATAGTAATACCTTTTATAATTGGAGGATTTGGTAATTTTTTAGTCCCTTTAATGCTGGGATCCCCAGATATAGCATACCCACGAATAAATAATATAAGATTCTGGCTACTCCCCCCTTCTATCACTCTCCTTTTATTAGGAAGATTCATTAATTCAGGAGCCGGAACTGGGTGAACAGTCTATCCCCCCCTGGCCTCAAACATTTTTCACAGGGGAGCTTCTATCGATCTCTCAATTTTCTCATTACATATTGCAGGAATATCTTCCATTCTTGGAGCTATTAATTTTATCTCCACAATTATTAATATACATCATAAAAATTTTACTATAGATAAAATTCCTTTATTAGTTTGATCAATTTTAATTACAGCAATCCTTCTTCTTCTCTCCCTNCCAGTCCTTGCTGGAGCAATTACTATACTCTTAACTGACCGTAATCTTAACACTTCCTTCTTTGACCCAGCTGGAGGGGGAGACCCCATTCTTTACCAACATCTANNNNNNNNNNNNNNNNNN>CASENT0121775_Pheidole_megacephalaTATACTTTACTTTATCTTTGCGATCTGATCTGGGATAATTGGGTCCTCTATGAGATTAATTATTCGATTAGAACTCGGATCCTGTAATTCCTTAATTAATAATGATCAAATTTATAATTCATTAGTTACAAGACATGCTTTTATTATAATCTTCTTTATAGTTATACCTTTTATAATTGGAGGATTTGGTAATTTTTTAGTCCCTTTAATGCTAGGATCTCCAGATATAGCATACCCACGNATAAATAATATAAGNTTCTGGCTACTCCCCCCTTCTATCACTCTCCTTTTATTAGGAAGATTCATTAATTCAGGAGCCGGAACTGGGTGAACAGTCTATCCCCCCCTAGCTTCAAACATTTTTCACAGGGGAGCTTCTATCGATCTCTCAATTTTCTCATTACATATTGCAGGNATATCTTCCATTCTTGGAGCTATTAATTTTATCTCCACAATTATTAATATACATCATAAAAATTTTACTATAGATAAAATTCCCTTATTAGTTTGATCAATTTTAATTACAGCAATCCTTCTTCTTCTCTCCCTACCAGTCCTTGCTGGAGCAATTACTATACTCTTAACTGACCGTAATCTTAACACTTCCTTCTTTGACCCAGCAGGAGGAGGAGACCCCATTCTTTACCAACATCTATTTNNNNNNNNNNNNNNN>CASENT0128129_Pheidole_megacephalaNATACTTTACTTTATCTTTGCGATCTGATCTGGGATAATTGGGTCCTCTATGAGATTAATTATTCGATTAGAACTCGGATCCTGTAATTCCTTAATTAATAATGATCAAATTTATAATTCATTAGTTACAAGACATGCTTTTATTATAATCTTCTTTATAGTTATACCTTTTATAATTGGAGGATTTGGTAATTTTTTAGTCCCTTTAATGCTAGGATCTCCAGATATAGCATACCCACGNATAAATAATATAAGATTCTGGCTACTCCCCCCTTCTATCACTCTCCTTTTATTAGGAAGATTCATTAATTCAGGAGCCGGAACTGGGTGAACAGTCTATCCCCCCCTAGCTTCAAACATTTTTCACAGGGGAGCTTCTATCGATCTCTCAATTTTCTCATTACATATTGCAGGGATATCTTCCATTCTTGGAGCTATTAATTTTATCTCCACAATTATTAATATACATCATAAAAATTTTACTATAGATAAAATTCCCTTATTAGTTTGATCAATTTTAATTACAGCAATCCTTCTTCTTCTCTCCCTACCAGTCCTTGCTGGAGCAATTACTATACTCTTAACTGACCGTAATCTTAACACTTCCTTCTTTGACCCAGCAGGAGGAGGAGACCCCATTCTTTACCAACATCTANNNNNNNNNNNNNNNNNN>CASENT0120476_Pheidole_megacephalaNATACTTTACTTTATCTTTGCGATCTGATCTGGAATAATTGGGTCCTCTATGAGATTAATTATTCGATTAGAACTCGGATCCTGTAATTCCTTAATTAATAATGATCAAATTTATAATTCATTAGTTACAAGACATGCTTTTATTATAATCTTCTTTATAGTTATACCTTTTATAATTGGAGGATTTGGTAATTTTTTAGTCCCTTTAATGTTAGGATCCCCAGATATAGCATACCCACGAATAAATAATATAAGATTCTGGCTACTNCCCCCTTCTATCACTCTCCTTTTATTAGGAAGATTCATTAATTCAGGAGCCGGAACTGGGTGAACAGTCTATCCCCCCCTAGCCTCAAACATTTTTCACAGAGGAGCTTCTATCGATCTCTCAATTTTCTCATTACATATTGCAGGAATATCTTCCATTCTTGGAGCTATTAATTTTATCTCNACAATTATTAATATACATCATAAAAATTTTACTATAGATAAAATTCCTTTATTAGTTTGATCAATTTTAATTACAGCAATCCTTCTTCTNCTCTCCCTACCAGTCCTTGCTGGAGCAATTACTATACTCTTAACTGACCGTAATCTTAACACTTCCTTCTTTGACCCAGCAGGAGGAGGAGACCCCATTCTTTACCAACATCTANNNNNNNNNNNNNNNNNN>CASENT0125351_Pheidole_megacephalaNATACTTTACTTTATCTTTGCGATCTGATCTGGAATAATTGGGTCCTCTATGAGATTAATTATTCGATTAGAACTCGGATCCTGTAATTCCTTAATTAATAATGATCAAATCTATAATTCATTAGTTACAAGACATGCTTTTATTATAATCTTCTTTATAGTTATACCTTTTATAATTGGAGGATTTGGTAATTTTTTAGTCCCTTTAATGNTAGGATCCCCAGATATAGCATACCCACGAATAAATAATATAAGATTCTGGCTACTNCCCCCTTCTATCACTCTCCTTTTATTAGGAAGATTCATTAATTCAGGAGCCGGAACTGGGTGAACAGTCTATCCCCCCCTAGCCTCAAACATTTTTCATAGAGGAGCTTCTATCGATCTCTCAATTTTCTCATTACATATTGCAGGAATATCTTCCATTCTTGGAGCTATTAATTTTATCTCCACAATTATTAATATACATCATAAAAATTTTACTATAGATAAAATTCCTTTATTAGTTTGATCAATTTTAATTACAGCAATCCTTCTTCTTCTCTCCCTNCCAGTCCTTGCTGGAGCAATTACTATACTCTTAACTGACCGTAATCTTAACACTTCCTTCTTTGACCCAGCAGGAGGGGGAGACCCCATTCTTTACCAACATCTATTTNNNNNNNNNNNNNNN>CASENT0122224_Pheidole_megacephalaNNNNNNNNNNNNNNNNNNNNNNNNNNNNNNNNNNNNNNNNNNNNNNNNNNNNNNNNNNNNNNNNNNNNNNNNNNNNNNNNNNNNNNNNNNNNNNNNNNNNNNNNNNNNNNNNTATAATTCATTAGTTACAAGACATGCTTTTATTATAATCTTCTTTATAGTNATACCTTTTATAATTGGAGGATTTGGTAATTTTTTAGTCCCTTTAATGTTAGGATCCCCAGATATAGCATACCCACGAATAAATAATATAAGATTCTGGCTACTTCCCCCTTCTATCACTCTCCTTTTATTAGGAAGATTCATTAATTCAGGAGCCGGAACTGGGTGAACAGTCTATCCCCCCCTAGCCTCAAACATTTTTCATAGAGGAGCTTCTATCGATCTCTCAATTTTCTCATTACATATTGCAGGAATATCTTCCATTCTTGGAGCTATTAATTTTATCTCTACAATTATTAATATACATCATAAAAATTTTACTATAGATAAAATTCCTTTATTAGTTTGATCAATTTTAATTACAGCAATCCTTCTTCTTCTCTCCCTACCAGTCCTTGCTGGAGCAATTACTATACTCTTAACTGACCGTAATCTTAACACTTCCTTCTTTGACCCANNNNNNNNNNNNNNNNNNNNNNNNNNNNNNNNNNNNNNNNNNNNNNNNNNNNNN>CASENT0125212_Pheidole_megacephalaNATACTTTACTTTATCTTTGCGATCTGATCTGGAATAATTGGGTCCTCTATGAGATTAATTATTCGATTAGAACTCGGATCCTGTAATTCCTTAATTAATAATGATCAAATCTATAATTCATTAGTTACAAGACATGCTTTTATTATAATCTTCTTTATAGTTATACCTTTTATAATTGGAGGATTTGGTAATTTTTTAGTCCCTTTAATGTTAGGATCCCCAGATATAGCATACCCACGAATAAATAATATAAGATTCTGGCTACTTCCCCCTTCTATCACTCTCCTTTTATTAGGAAGATTCATTAATTCAGGAGCCGGAACTGGGTGAACAGTCTATCCCCCCCTAGCCTCAAACATTTTTCATAGAGGAGCTTCTATCGATCTCTCAATTTTCTCATTACATATTGCAGGAATATCTTCCATTCTTGGAGCTATTAATTTTATCTCTACAATTATTAATATACATCATAAAAATTTTACTATAGATAAAATTCCTTTATTAGTTTGATCAATTTTAATTACAGCAATCCTTCTTCTCCTCTCCCTACCAGTCCTTGCTGGAGCAATTACTATACTCTTAACTGACCGTAATCTTAACACTTCCTTCTTTGACCCAGCGGGAGGGGGAGACCCCATTCTTTACCAACATCTATTTNNNNNNNNNNNNNNN>CASENT0116105_Pheidole_megacephalaTATACTTTACTTTATCTTTGCGATCTGATCTGGAATAATTGGGTCCTCTATGAGATTAATTATTCGATTAGAACTCGGATCCTGTAATTCCTTAATTAATAATGATCAAATCTATAATTCATTAGTTACAAGACATGCTTTTATTATAATCTTCTTTATAGTTATACCTTTTATAATTGGAGGATTTGGTAATTTTTTAGTCCCTTTAATGTTAGGATCCCCAGATATAGCATACCCACGAATAAATAATATAAGATTCTGGCTACTTCCCCCTTCTATCACTCTCCTTTTATTAGGAAGATTCATTAATTCAGGAGCCGGAACTGGGTGAACAGTCTATCCACCCCTAGCCTCAAACATTTTTCATAGAGGAGCTTCTATCGATCTCTCAATTTTCTCATTACATATTGCAGGAATATCTTCCATTCTTGGAGCTATTAATTTTATCTCTACAATTATTAATATACATCATAAAAATTTTACTATAGATAAAATTCCTTTATTAGTTTGATCAATTTTAATTACAGCAATCCTTCTTCTCCTCTCCCTACCAGTCCTTGCTGGAGCAATTACTATACTCTTAACTGACCGTAATCTTAACACTTCCTTCTTTGACCCAGCAGGAGGGGGAGACCCCATTCTTTACCAACATCTATTTNNNNNNNNNNNNNNN>CASENT0158882_Pheidole_megacephalaTATACTTTACTTTATCTTTGCGATCTGATCTGGAATAATTGGGTCCTCTATGAGATTAATTATTCGATTAGAACTCGGATCCTGTAATTCCTTAATTAATAATGATCAAATCTATAATTCATTAGTTACAAGACATGCTTTTATTATAATCTTCTTTATAGTTATACCTTTTATAATTGGAGGATTTGGTAATTTTTTAGTCCCTTTAATGNTAGGATCCCCAGATATAGCATACCCACGAATAAATAATATAAGATTCTGGCTACTTCCCCCTTCTATCACTCTCCTTTTATTAGGAAGATTCATTAATTCAGGAGCCGGAACTGGGTGAACAGTCTATCCACCCCTAGCCTCAAACATTTTTCATAGAGGAGCTTCTATCGATCTCTCAATTTTCTCATTACATATTGCAGGAATATCTTCCATTCTTGGAGCTATTAATTTTATCTCTACAATTATTAATATACATCATAAAAATTTTACTATAGATAAAATTCCTTTATTAGTTTGATCAATTTTAATTACAGCAATCCTTCTTCTCCTCTCCCTACCAGTCCTTGCTGGAGCAATTACTATACTCTTAACTGACCGTAATCTTAACACTTCCTTCTTTGACCCAGCAGGAGGGGGAGACCCCATTCTTTACCAACATCTATTTNNNNNNNNNNNNNNN>CASENT0107681_Pheidole_megatronNATACTTTATTTTATCTTTGCAATCTGATCTGGAATAATTGGATCCTCTATGAGTATAATTATCCGATTAGAACTCGGATCCTGTAATTCCTTAATCAATAATGATCAAATTTATAACTCATTAGTTACAAGACATGCTTTTATTATAATCTTCTTTATAGTAATACCTTTTATAATTGGAGGATTTGGTAATTTTCTAGTCCCCCTAATACTTGGATCCCCAGATATAGCCTATCCCCGAATAAATAATATAAGATTTTGATTACTCCCGCCTTCTATTACTCTCCTCTTACTAGGAAGATTCATTAATTCAGGAGTCGGAACTGGATGAACAGTTTATCCCCCTTTAGCCTCTAATGTTTTCCACAGAGGAGCCTCTATTGACCTCTCAATTTTCTCACTACATATTGCAGGAATATCTTCTATTCTGGGTGCTATTAATTTTATTGCTACAATTATTAATATACATCATAAAAATTTTACTATAGATAAAATTCCTTTATTAGTTTGATCAATTTTAATCACAGCAATTCTTCTACTTCTCTCCCTCCCAGTTCTCGCCGGAGCAATTACTATACTTTTAACTGACCGTAATCTTAACACTTCATTTTTTGACCCCGCAGGAGAGGGGGACCCCATTCTTTACCAACACNTCTTTNNNNNNNNNNNNNNN>CASENT0122962_Pheidole_megatronAATACTTTATTTTATCTTTGCAATCTGATCTGGAATAATTGGATCCTCTATGAGTATAATTATCCGATTAGAACTCGGATCCTGTAATTCCTTAATCAATAATGATCAAATTTATAACTCATTAGTTACAAGACATGCTTTTATTATAATCTTCTTTATAGTAATACCTTTTATAATTGGAGGATTTGGTAATTTTCTAGTCCCCCTAATACTTGGATCCCCAGATATAGCCTATCCCCGAATAAATAATATAAGATTTTGATTACTCCCGCCTTCTATTACTCTCCTCTTACTAGGAAGATTCATTAATTCAGGAGTCGGAACTGGATGAACAGTTTACCCCCCTTTAGCCTCTAATGTTTTCCACAGAGGAGCCTCTATTGACCTCTCAATTTTCTCACTACATATTGCAGGAATATCTTCTATTCTGGGTGCTATTAATTTTATTGCTACAATTATTAATATACATCATAAAAATTTTACTATAGATAAAATTCCTTTATTAGTTTGATCAATTTTAATCACAGCAATTCTTCTACTTCTCTCCCTCCCAGTTCTCGCCGGAGCAATTACTATACTTTTAACTGACCGTAATCTTAACACTTCATTTTNNNNNNNNNNNNNNNNNNNNNNNNNNNNNNNNNNNNNNNNNNNNNNNNNNNNNNNNNNNNNN>CASENT0137370_Pheidole_megatronAATACTTTATTTTATCTTTGCAATCTGATCTGGAATAATTGGATCCTCTATGAGTATAATTATCCGATTAGAACTCGGATCCTGTAATTCCTTAATCAATAATGATCAAATTTATAACTCATTAGTTACAAGACATGCTTTTATTATAATCTTCTTTATAGTAATACCTTTTATAATTGGAGGATTTGGTAATTTTCTAGTCCCCCTAATACTTGGATCCCCAGATATAGCCTATCCCCGAATAAATAATATAAGATTTTGATTACTCCCGCCTTCTATTACTCTCCTCTTACTAGGAAGATTCATTAATTCAGGAGTCGGAACTGGATGAACAGTTTACCCCCCTTTAGCCTCTAATGTTTTCCACAGAGGAGCCTCTATTGACCTCTCAATTTTCTCACTACATATTGCAGGAATATCTTCTATTCTTGGTGCTATTAATTTTATTGCTACAATTATTAATATACATCATAAAAATTTTACTATAGATAAAATTCCTTTATTAGTTTGATCAATTTTAATCACAGCAATTCTTCTACTTCTCTCCCTCCCAGTTCTCGCCGGAGCAATTACTATACTTTTAACTGACCGTAATCTTAACACTTCATTTTTTGACCCCGCAGGAGGAGGGGACCCCATTCTTTACCAACACCTCTTTNNNNNNNNNNNNNNN>CASENT0147141_Pheidole_megatronAATACTTTACTTTATCTTTGCAATCTGATCTGGAATAATTGGGTCCTCTATGAGAATAATTATCCGATTAGAACTCGGATCCTGTAATTCCTTAATCAATAATGACCAAATTTATAACTCATTAGTTACAAGACATGCTTTTATTATAATCTTCTTTATAGTAATACCCTTTATAATTGGGGGATTTGGTAACTTTCTAGTCCCCCTAATACTTGGGTCTCCAGATATAGCATACCCCCGAATAAATAATATAAGATTTTGATTACTTCCCCCTTCTATTACTCTCCTCTTACTAGGAAGATTCATTAATTCAGGGGTCGGAACTGGATGAACAGTTTACCCCCCTCTAGCCTCTAATGTTTTTCATAGAGGAGCCTCTATTGATCTCTCAATTTTCTCGCTACATATTGCAGGAATATCTTCCATTCTTGGAGCTATTAATTTTATTGCTACAATTATTAATATACATCATAAAAATTTTACTATAGATAAAATTCCTTTGTTAGTTTGATCAATTTTAATCACAGCAATCCTTCTACTTCTTTCCCTTCCAGTTCTCGCCGGAGCAATTACCATACTTTTAACTGACCGTAATCTTAACACTTCATTTTTTGACCCCGCAGGAGGAGGAGACCCCATTCTTTACCAACACCTCTTTNNNNNNNNNNNNNNN>CASENT0136865_Pheidole_megatronAATACTTTACTTTATCTTTGCAATCTGATCTGGAATAATTGGNTCCTCTATGAGAATAATTATCCGATTAGAACTCGGATCCTGTAATTCCTTAATCAATAATGACCAAATTTATAACTCATTAGTTACAAGACATGCTTTTATTATAATCTTCTTTATAGTAATACCCTTTATAATTGGGGGATTTGGTAACTTTCTAGTCCCCCTAATACTTGGGTCTCCAGATATAGCATACCCCCGAATAAATAATATAAGATTTTGATTACTTCCCCCTTCTATTACTCTCCTCTTACTAGGAAGATTCATTAATTCAGGGGTCGGAACTGGATGAACAGTTTACCCCCCTCTAGCCTCTAATGTTTTTCATAGAGGAGCCTCTATTGATCTCTCAATTTTCTCGCTACATATTGCAGGAATATCTTCCATTCTTGGAGCTATTAATTTTATTGCTACAATTATTAATATACATCATAAAAATTTTACTATAGATAAAATTCCTTTGTTAGTTTGATCAATTTTAATCACAGCAATCCTTCTACTTCTTTCCCTTCCAGTTCTCGCCGGAGCAATTACCATACTTTTAACTGACCGTAATCTTAACACTTCATTTTTTGACCCCGCAGGAGGAGGAGACCCCATTCTTTACCAACACCTCTTTNNNNNNNNNNNNNNN>CASENT0136919_Pheidole_megatronAATACTTTACTTTATCTTTGCAATCTGATCTGGAATAATTGGATCCTCTATGAGGATAATTATCCGATTAGAACTCGGATCCTGTAATTCCTTAATTAATAATGACCAAATTTATAACTCATTAGTTACAAGACATGCTTTTATTATAATCTTCTTTATAGTAATACCATTTATAATTGGGGGATTTGGTAATTTTCTAGTCCCCCTGATACTTGGGTCTCCAGATATAGCCTACCCCCGAATAAATAATATAAGATTTTGATTACTCCTTCCTTCTATTACTCTCCTCTTACTAGGAAGATTCATTAATTCAGGAGTCGGAACTGGATGAACAGTTTACCCCCCTCTAGCCTCTAATGTTTTTCACAGAGGAGCCTCTATTGACCTCTCAATTTTCTCACTACATATTGCAGGAATATCTTCCATTCTTGGCGCTATTAATTTTATTGCTACAATTATTAATATACATCATAAAAATTTTACTATAGATAAAATTCCTTTATTAGTTTGATCAATTTTAATCACAGCAATTCTTCTACTTCTTTCCCTCCCAGTTCTCGCCGGAGCAATTACCATACTTTTAACTGACCGTAATCTTAACACTTCATTTTTTGACCCCGCAGGAGGAGGAGACCCCATTCTTTACCAACACCTCTTTNNNNNNNNNNNNNNN>CASENT0054356_Pheidole_spinosaTGTACTTTACTTTATCTTTGCAATCTGATCTGGAATAATTGGATCCTCTATGAGATTAATTATTCGATTAGAACTCGGATCCTGTAACTCCTTAATTAATAATGATCAAATTTATAACTCATTAGTTACAAGACACGCTTTCATTATAATCTTCTTTATAGTAATACCTTTCATAATTGGAGGATTTGGTAACTTCCTAGTCCCTTTAATACTTGGCTCCCCAGATATAGCCTACCCACGAATAAATAATATAAGATTCTGACTCCTCCCACCTTCTATCACTCTTCTCTTATTAGGAAGATTCATTAATTCAGGAGCCGGAACTGGATGAACAGTTTATCCCCCTCTAGCCTCTAATATTTTTCATAGAGGAGCCTCTATTGACCTCTCAATTTTCTCCTTACATATTGCGGGAATGTCTTCCATTCTTGGAGCTATTAATTTTATTTCTACAATTATTAATATACATCATAAGAATTTTACTATAGATAAAATTCCTTTATTAGTTTGATCAATTTTGATTACAGCAATTCTTCTTCTTCTCTCCCTCCCAGTCCTCGCTGGAGCAATTACCATACTTTTAACTGACCGTAACCTTAACACATCCTTCTTTGACCCCGCAGGGGGAGGAGACNNNNNNNNNNNNNNNNNNNNNNNNNNNNNNNNNNNNNNN>CASENT0070840_Pheidole_spinosaTATACTTTACTTTATCTTTGCAATCTGATCTGGGTTAATTGGATCCTCTATGAGATTAATTATTCGATTAGAACTCGGATCCTGTAACTCCTTAATTAATAACGATCAAATTTATAACTCATTAGTTACAAGACATGCTTTCATTATAATCTTCTTTATAGTAATACCTTTCATAATTGGGGGATTTGGAAATTTCCTAGTCCCTTTAATACTTGGTTCCCCAGATATGGCCTACCCACGAATAAATAATATAAGATTTTGACTCCTCCCCCCTTCTATTACTCTTCTCTTATTAGGAAGATTCATTAATTCAGGAGCCGGAACTGGATGAACTGTTTACCCTCCTCTGGCCTCTAATATTTTCCATAGAGGAGCCTCTATTGACCTATCAATTTTCTCTTTACATATTGCAGGGATGTCTTCTATTCTCGGAGCAATCAATTTTATTTCTACAATTATTAATATACATCATAAAAATTTTACGATAGATAAGATTCCTTTATTAGTTTGATCAATTTTAATTACAGCTATTCTGCTTCTTCTCTCACTCCCAGTACTTGCTGGAGCAATTACCATACTTTTAACTGACCGTAACCTTAACACATCCTTCTTTGACCCCGCAGGAGGAGGAGATCCCATTCTTTACCAACATCTCTTTNNNNNNNNNNNNNNN>CASENT0060288_Pheidole_spinosaNATACTTTACTTTATCTTTGCAATCTGATCTGGGTTAATTGGATCCTCTATGAGATTAATTATTCGATTAGAACTCGGATCCTGTAACTCCTTAATTAATAACGATCAAATTTATAACTCATTAGTTACAAGACATGCTTTCATTATAATCTTCTTTATAGTAATACCTTTCATAATTGGGGGATTTGGAAATTTCCTAGTCCCTTTAATACTTGGTTCCCCAGATATGGCCTACCCACGAATAAATAATATAAGATTTTGACTCCTCCCCCCTTCTATTACTCTTCTCTTATTAGGAAGATTCATTAATTCAGGAGCCGGAACTGGATGAACTGTTTACCCTCCTCTGGCCTCTAATATTTTCCATAGAGGAGCCTCTATTGACCTATCAATTTTCTCCTTACATATTGCAGGGATGTCTTCTATTCTCGGAGCAATCAATTTTATTTCTACAATTATTAATATACATCATAAAAATTTTACGATAGATAAGATTCCTTTATTAGTTTGATCAATTTTAATTACAGCTATTCTGCTTCTTCTCTCACTCCCAGTACTTGCTGGAGCAATTACCATACTTTTAACTGACCGTAACCTTAACACATCCTTCTTTGACCCCGCAGGAGGAGGAGACCCCATTCTTTACCAACATCTCNNNNNNNNNNNNNNNNNN>CASENT0198048_Pheidole_spinosaNNNNNNNNNNNNNNNNNNNNNNNTTTGATCTGGATTAATTGGATCCTCTATGAGATTAATTATTCGATTAGAACTCGGATCCTGTAACTCCTTAATTAATAATGATCAAATTTATAACTCATTAGTTACAAGACATGCTTTCATTATAATCTTCTTTATAGTAATACCCTTCATAATTGGAGGATTTGGTAATTTCCTAGTCCCTTTAATACTTGGCTCTCCAGATATAGCCTACCCACGAATAAATAATATAAGATTTTGACTCCTCCCCCCATCTATTACTCTTCTCTTATTAGGAAGATTCATTAATTCAGGAGCCGGAACTGGGTGAACAGTTTACCCCCCTCTGGCCTCTAATATTTTTCATAGAGGAGCCTCTATTGACCTCTCAATTTTCTCCTTACATATTGCAGGAATGTCTTCTATTCTTGGGGCTATCAATTTTATTTCTACAATTATTAATATACATCATAAAAATTTTACAATAGATAAAATTCCTTTATTAGTTTGATCAATTTTAATTACAGCAATCCTTCTTCTTCTCTCACTTCCAGTACTTGCTGGAGCAATTACCATACTTTTAACTGACCGTAACCTTAACACATCCTTCTTTGACCCCGCAGGTGGAGGAGACCCCATTCTTTACCAGCATCTATTTNNNNNNNNNNNNNNN>CASENT0051399_Pheidole_spinosaNNNNNNNNNNNNNNNNNNNNNNNNNNNNNNNNNNNNNNNNNNNNNNNNNNNNNNNNNNNNNNNNNNNNNNNNNNNNNNNNNNNNTAACTCCTTAATTAATAATGATCAAATTTATAACTCATTAGTTACAAGACATGCTTTCATTATAATCTTCTTTATAGTAATACCTTTCATAATTGGAGGATTTGGTAATTTTCTAGTCCCTTTAATACTTGGTTCCCCAGATATAGCCTACCCACGAATAAATAATATAAGATTTTGACTCCTCCCCCCTTCTATTACTCTTCTCTTATTAGGAAGATTTATTAATTCAGGAGCCGGAACTGGATGAACAGTTTACCCTCCTCTAGCCTCTAATATTTTTCATAGAGGAGCCTCTATCGATCTATCAATTTTCTCCTTACATATTGCAGGAATATCTTCTATTCTTGGCGCAATCAATTTTATTTCTACAATTATTAATATACATCATAAAAATTTTACTATAGATAAAATTCCTTTATTAGTTTGATCAATTTTAATTACAGCAATTCTTCTTCTTCTCTCACTCCCAGTACTTGCTGGAGCAATTACCATACTTTTAACTGACCGTAACCTTANNNNNNNNNNNNNNNNNNNNNNNNNNNNNNNNNNNNNNNNNNNNNNNNNNNNNNNNNNNNNNNNNNNNNNNNNN>CASENT0489569_Pheidole_spinosaTATACTTTACTTTATCTTTGCAATCTGATCTGGATTAATTGGATCCTCTATAAGATTAATTATTCGATTAGAACTCGGATCCTGTAACTCCTTAATTAATAATGATCAAATTTATAACTCATTAGTCACAAGACATGCTTTCATTATAATCTTCTTTATAGTAATACCTTTCATAATTGGAGGATTTGGTAATTTCCTAGTCCCTTTAATACTTGGTTCCCCCGATATAGCCTACCCACGAATAAATAATATAAGATTTTGACTCCTCCCCCCTTCTATTACTCTTCTCTTATTAGGAAGATTTATTAATTCAGGAGCCGGAACTGGATGAACAGTTTACCCTCCTCTAGCCTCTAATATTTTTCATAGAGGAGCCTCTATCGACCTATCAATTTTCTCCTTACATATTGCAGGAATGTCCTCTATTCTTGGTGCAATCAATTTTATTTCTACAATTATTAATATACATCATAAAAATTTTACAATAGATAAAATTCCTTTATTAGTTTGATCAATTTTAATTACAGCAATTCTTCTTCTTCTCTCACTCCCAGTACTTGCTGGAGCAATTACCATACTTTTAACTGACCGTAACCTTAACACATCCTTCTTTGACCCCGCAGGAGGAGGAGACCCTATTCTTTACCAACATCTCTTTNNNNNNNNNNNNNNN>CASENT0122074_Pheidole_spinosaNNNNNNNNNNNNNNNNNNNNNNNNNNNNNNNGGAATAATTGGATCCTCTATGAGATTAATTATTCGATTAGAACTCGGGTCCTGTAACTCCCTAATTAATAATGATCAAATTTATAATTCATTAGTTACAAGGCACGCTTTCATTATAATCTTTTTTATAGTAATGCCTTTTATAATTGGAGGATTTGGTAATTTCCTAGTCCCTTTAATACTTGGCGCTCCAGATATAGCATACCCACGAATAAATAATATAAGATTCTGACTCCTTCCACCTTCTATCACTCTTCTCTTACTAAGAAGATTCATTAATTCAGGGACTGGAACAGGATGAACAGTTTACCCCCCTCTAGCCTCTAATATTTTTCATAGAGGAGCCTCTATTGACCTCTCAATTTTCTCCTTACATATTGCAGGAATATCTTCCATTCTTGGTGCTATTAATTTTATCTCTACAATTATCAATATACATCACAAAAATTTTACTATAGATAAAATTCCTTTATTAGTTTGATCAATTTTAATTACAGCAATTCTCCTTCTTCTCTCTCTCCCAGTCCTCGCAGGAGCAATTACCATACTTTTAACTGACCGTAACCTTAATACATCCTTCTTTGACCCCGCAGGAGGAGGTGNNNNNNNNNNNNNNNNNNNNNNNNNNNNNNNNNNNNNNNNN>CASENT0060316_Pheidole_spinosaNGTACTTTACTTTATCTTTGCAATCTGATCTGGAATAATTGGATCCTCTATAAGATTAATTATTCGATTAGAACTCGGGTCCTGTAACTCCCTAATTAATAATGATCAAATTTATAATTCATTAGTTACAAGGCACGCTTTCATTATAATCTTTTTTATAGTAATACCTTTTATAATTGGAGGATTTGGTAATTTCCTAGTCCCTTTAATACTTGGCTCTCCAGATATAGCATACCCACGAATAAATAATATAAGATTCTGACTCCTTCCGCCTTCTATCACTCTTCTCTTACTAAGAAGATTCATTAATTCAGGGACTGGAACAGGATGAACAGTTTACCCCCCTCTAGCCTCTAATATTTTTCATAGAGGAGCCTCTATTGACCTCTCAATTTTCTCCTTACATATTGCAGGAATATCTTCCATTCTTGGAGCTATTAATTTTATCTCTACAATTATCAATATACATCATAAAAATTTTACTATAGATAAAATTCCTTTATTAGTTTGATCAATTTTAATTACAGCAATTCTCCTTCTTCTCTCTCTCCCAGTCCTCGCAGGAGCAATTACCATACTTTTAACTGACCGTAACCTTAATACATCCTTCTTTGACCCGGCAGGAGGAGGAGACCCCATTCTTTACCAACATCTCNNNNNNNNNNNNNNNNNN>CASENT0121810_Pheidole_spinosaNNNNNNNNNNNNNNNNNNNNNNNNNNNNNNNNNNNNNATTGGATCCTCTATAAGATTAATTATTCGATTAGAACTCGGGTCCTGTAACTCCCTAATTAATAATGATCAAATTTATAATTCATTAGTTACAAGGCACGCTTTCATTATAATCTTTTTTATAGTAATGCCTTTTATAATTGGAGGATTTGGTAATTTCCTAGTCCCTTTAATACTTGGCTCTCCAGATATAGCATACCCACGAATAAATAATATAAGATTCTGACTCCTTCCACCTTCTATCACTCTTCTCTTACTAAGAAGATTCATTAATTCAGGGACTGGAACNGGATGAACTNNNNNNNNNNNNNNNNNNNNNNNNNNNNNNNNNNNNNNNNNNNNNNNNNNNNNNNNNNNNNNNNNNNNNNNNNNNNNNNNNNNNNNNNNNNNNNNNNNNNNNNNNNNNNNNNNNNNNNNNNNNNNNNNNNNNNNNNNNNNNNNNNNNNNNNNNNNNNNNNNNNNNNNNNNNNNNNNNNNNNNNNNNNNNNNNNNNNNNNNNNNNNNNNNNNNNNNNNNNNNNNNNNNNNNNNNNNNNNNNNNNNNNNNNNNNNNNNNNNNNNNNNNNNNNNNNNNNNNNNNNNNNNNNNNNNNNNNNNNNNNNNNNNNNNNNNNNNNNNNNNNNNNNNNNNNNNNNNNN>CASENT0107190_Pheidole_spinosaNGTACTTTACTTTATCTTTGCAATCTGATCTGGAATAATTGGATCATCTATGAGATTAATTATTCGACTAGAACTCGGATCCTGCAACTCCTTAATTAATAATGATCAAATTTATAATTCATTAGTCACAAGACACGCTTTTATTATAATCTTTTTTATGGTAATACCTTTCATAATTGGGGGATTTGGTAATTTCCTAGTCCCTTTAATGCTCGGCTCCCCAGATATAGCCTACCCACGAATAAATAATATAAGTTTCTGACTCCTCCCCCCTTCTATCACTCTTCTTTTACTAGGAAGATTCATTAATTCAGGAGCCGGAACAGGATGAACAGTTTACCCCCCTCTAGCCTCTAATATCTTTCACAGGGGAGCCTCTATTGACCTCTCAATTTTCTCTTTACATATTGCAGGAATATCTTCCATTCTTGGAGCTATCAATTTTATCTCTACAATTATTAATATACATCATAAAAATTTTACTATAGACAAAATTCCTTTATTAGTCTGATCAATCTTAATTACAGCAATTCTTCTTCTTCTATCCCTCCCCGTCCTCGCAGGAGCAATTACTATACTATTAACTGACCGTAATCTTAATACATCCTTCTTTGACCCCGCGGGAGGGGGAGACCCCATTCTTTACCAACATCTCTTTNNNNNNNNNNNNNNN>CASENT0198046|Pheidole_spinosaTGTACTTTACTTTATCTTTGCAATCTGATCTGGAATAATTGGATCATCTATGAGATTAATTATTCGGCTAGAACTCGGATCCTGCAACTCCTTAATTAATAATGATCAAATTTATAATTCATTAGTTACAAGACACGCTTTTATTATAATCTTTTTTATAGTAATACCTTTCATAATTGGAGGATTTGGTAATTTCCTAGTCCCTTTAATGCTTGGCTCTCCAGATATGGCCTACCCACGAATAAATAATATAAGTTTCTGACTCCTCCCCCCTTCTATCACTCTTCTTTTACTAGGAAGATTCATTAATTCAGGAGCCGGAACAGGATGAACAGTTTATCCCCCTCTAGCCTCTAATATCTTTCACAGGGGAGCCTCTATTGATCTCTCAATTTTCTCTTTACATATTGCAGGAATATCTTCCATTCTTGGAGCTATCAATTTTATCTCTACAATTATTAATATACATCATAAAAATTTTACTATAGACAAAATTCCTTTATTAGTCTGATCAATCTTAATTACAGCAATTCTCCTTCTTCTATCCCTCCCCGTCCTCGCAGGAGCAATTACTATACTATTAACTGACCGTAATCTTAATACATCCTTCTTTGACCCCGCAGGAGGGGGAGACCCCATTCTTTACCAACATCTCTTTNNNNNNNNNNNNNNN>CASENT0196399_Pheidole_decepticonNNNNNNNNNNNNNNNNNNNNNNNNNNNNNNNNNNNNAATTGGTTCCTCTATGAGATTAATTATTCGATTGGAACTCGGATCCTGTAACTCCTTAATTAATAATGATCAAATTTATAATTCATTAGTTACAAGACACGCTTTCATTATAATCTTTTTTATAGTAATACCTTTCATAATTGGGGGATTTGGTAATTTCTTAGTCCCTTTAATACTTGGTTCTCCAGATATAGCCTACCCACGAATAAATAATATAAGGTTTTGACTCCTCCCCCCTTCTATCACTCTTCTCTTACTAGGAAGATTCATTAATTCAGGAGCCGGAACAGGATGAACAGTTTACCCCCCTCTGGCCTCTAATATTTTTCACAGAGGAGCTTCTATTGACCTCTCAATTTTTTCCTTACATATTGCAGGAATATCTTCTATTCTTGGAGCTATCAATTTTATCTCTACAATTATTAATATACATCATAAAAATTTTACTATAGATAAAATTCCTTTATTAGTCTGATCAATTTTAATTACAGCAGTTCTCCTTCTTCTCTCCCTTCCCGTTCTAGCAGGAGCAATTACTATACTTTTAACTGACCGTAACCTTAATACATCCTTCTTTGACCCCGCAGGAGGAGGAGACCCCATTCTTTATCAACATCTCTTTNNNNNNNNNNNNNNN>CASENT0128369_Pheidole_spinosaTGTACTTTACTTTATCTTTGCAATCTGATCTGGAATAATTGGATCCTCTATGAGATTAATTATTCGATTAGAACTCGGATCATGTAACTCCTTAATTAATAATGATCAAATTTATAATTCATTAGTTACAAGGCACGCTTTCATTATAATCTTTTTTATAGTAATACCTTTTATAATTGGGGGATTTGGTAATTTTCTAGTCCCTTTAATACTTGGTTCCCCAGATATAGCCTACCCACGAATAAATAATATAAGATTCTGACTCCTCCCACCTTCTATTACTCTTCTATTATTAGGAAGATTCATTAATTCAGGGGCAGGAACTGGATGAACAGTCTACCCTCCTCTAGCCTCTAATATTTTTCATAGGGGGGCCTCTATTGACCTCTCAATTTTCTCCTTACATATTGCAGGAATATCTTCCATTCTTGGAGCTATTAATTTTATCTCTACAATTATTAATATACATCATAAAAATTTTACTATAGATAAAATTCCTCTATTAGTTTGATCAATTTTAATTACAGCTATTCTCCTTCTTTTATCCCTCCCAGTCCTCGCAGGAGCAATTACAATACTTTTAACTGACCGTAATCTTAATACATCCTTCTTTGACCCCGCAGGAGGAGGAGATCCCATTCTGTACCAACATCTCTTTNNNNNNNNNNNNNNN>CASENT0118991_Pheidole_spinosaTGTACTTTACTTTATCTTTGCAATCTGATCTGGAATAATTGGATCCTCTATGAGATTAATTATTCGATTAGAACTCGGATCATGTAACTCCTTAATTAATAATGATCAAATTTATAATTCATTAGTTACAAGACACGCTTTCATTATAATCTTTTTTATAGTAATACCTTTTATAATTGGGGGATTTGGTAATTTTCTAGTCCCTTTAATACTTGGTTCCCCAGATATAGCCTACCCACGAATAAATAATATAAGATTCTGACTCCTCCCACCTTCTATTACTCTTCTATTATTAGGAAGATTCATTAATTCAGGAGCCGGAACTGGATGAACAGTCTATCCTCCTCTAGCCTCTAATATTTTTCATAGAGGGGCCTCTATTGACCTCTCAATTTTCTCCTTACATATTGCAGGAATATCTTCCATTCTTGGAGCTATTAATTTTATCTCTACAATTATTAATATACATCATAAAAATTTTACTATAGATAAAATTCCTCTATTAGTTTGATCAATTTTAATTACAGCTATTCTCCTTCTTTTATCCCTCCCAGTCCTCGCAGGAGCAATTACAATACTTTTAACTGACCGTAATCTTAATACATCCTTCTTTGACCCCGCAGGAGGAGGAGATCCCATTCTTTACCAACATCTCTTTNNNNNNNNNNNNNNN>CASENT0165863_Pheidole_spinosaTGTACTTTACTTTATCTTTGCAATCTGATCTGGAATAATTGGATCCTCTATGAGATTAATTATTCGATTAGAACTCGGATCATGTAACTCCTTAATTAATAATGATCAAATTTATAATTCATTAGTTACAAGACACGCTTTCATTATAATCTTTTTTATAGTAATACCTTTTATAATTGGAGGATTTGGTAATTTTCTAGTCCCTTTAATACTTGGTTCCCCAGATATAGCCTACCCACGAATAAATAATATAAGATTCTGACTCCTCCCACCTTCTATTACTCTTCTATTATTAGGAAGATTCATTAATTCAGGAGCCGGAACTGGATGAACAGTCTACCCTCCTCTAGCCTCTAATATTTTTCATAGAGGGGCCTCTATTGACCTCTCAATTTTCTCCTTACATATTGCAGGAATATCTTCCATTCTTGGAGCTATTAATTTTATCTCTACAATTATTAATATACATCATAAAAATTTTACTATAGATAAAATTCCTCTATTAGTTTGATCAATTTTAATTACAGCTATTCTCCTACTTTTATCCCTCCCAGTCCTCGCAGGAGCAATTACAATACTTTTAACTGACCGTAATCTTAATACATCCTTCTTTGACCCTGCAGGAGGAGGAGATCCCATTCTTTACCAACATCTCTTTNNNNNNNNNNNNNNN>CASENT0214679_Pheidole_spinosaTGTACTTTACTTTATCTTTGCAATCTGATCTGGAATAATTGGATCCTCTATGAGATTAATTATTCGATTAGAACTCGGATCATGTAACTCCTTAATTAATAATGATCAAATTTATAATTCATTAGTTACAAGACACGCTTTCATTATAATCTTTTTTATAGTAATACCTTTTATAATTGGGGGATTTGGTAATTTTCTAGTCCCTTTAATACTTGGTTCCCCAGATATAGCCTACCCACGAATAAATAATATAAGATTCTGACTCCTCCCACCTTCTATTACTCTTCTATTATTAGGAAGATTCATTAATTCAGGAGCCGGAACTGGATGAACAGTCTACCCTCCTCTAGCCTCTAATATTTTTCATAGAGGGGCCTCTATTGACATCTCAATTTTCTCCTTACATATTGCAGGAATATCTTCCATTCTTGGAGCTATTAATTTTATCTCTACAATTATTAATATACATCATAAAAATTTTACTATAGATAAAATTCCTCTATTAGTTTGATCAATTTTAATTACAGCTATTCTCCTACTTTTATCCCTCCCAGTCCTCGCAGGAGCAATTACAATACTTTTAACTGACCGTAATCTTAATACATCCTTCTTTGACCCCGCAGGAGGAGGAGATCCCATTCTTTACCAACATCTCTTTNNNNNNNNNNNNNNN>CASENT0212407_Pheidole_spinosaNGTACTTTACTTTATCTTTGCAATCTGATCTGGAATAATTGGGTCCTCTATGAGATTAATTATTCGATTAGAACTCGGATCCTGTAACTCCTTAATTAATAATGATCAAATTTATAATTCATTAGTTACAAGACACGCTTTCATTATAATCTTTTTTATAGTAATACCTTTCATAATTGGGGGATTTGGTAATTTCCTAGTCCCTTTAATACTTGGCTCACCTGATATAGCCTACCCACGAATAAATAATATAAGTTTCTGACTCCTCCCCCCTTCTATCACTCTTCTCTTGCTAGGGAGATTCATTAATTCAGGGGCCGGAACAGGATGAACAGTTTACCCTCCTTTAGCCTCTAATGTCTTTCATAGGGGAGCCTCTATTGACCTATCAATTTTCTCCTTACATATTGCAGGAATATCTTCCATTCTTGGAGCTATTAATTTTATCTCTACAATTATTAATATACATCATAAAAATTTTACTATAGATAAAATTCCTTTATTAGTTTGATCAATTTTAATTACAGCTATTCTCCTTCTTCTCTCCCTCCCCGTCCTCGCAGGAGCGATTACTATACTTTTAACTGACCGTAACCTTAATACATCCTTCTTTGACCCCGCAGGAGGAGGAGACCCCATTCTTTACCAACATCTCTTNNNNNNNNNNNNNNNN>CASENT0245096_Pheidole_spinosaNNTGCTTTACTTTATCTTTGCAATCTGATCTGGTATAATTGGGTCCTCTATAAGACTAATTATTCGATTAGAACTCGGGTCCTGTAATTCCTTAATTAATAATGATCAAATTTATAATTCATTAGTTACAAGACACGCTTTCATTATAATCTTTTTTATAGTAATACCCTTCATAATTGGAGGATTTGGTAATTTCCTAGTCCCTTTAATACTTGGTTCTCCAGATATAGCCTACCCACGAATAAATAATATAAGATTCTGACTCCTCCCACCTTCTATTACTCTTCTCTTACTAGGAAGATTCATTAATTCAGGAGCCGGAACAGGATGAACAGTTTACCCCCCTCTAGCCTCTAATATTTTTCATAGGGGGGCCTCTATTGACCTCTCAATTTTCTCATTACATATTGCAGGAATGTCTTCCATTCTTGGAGCTATTAATTTTATTTCTACAATTATTAATATACATCATAAAAATTTTACTATAGATAAAATTCCCTTATTAGTTTGATCAATTTTAATTACAGCAATTCTCCTTCTTCTCTCCCTCCCAGTCCTCGCAGGAGCAATTACCATACTTTTAACTGACCGTAACCTTAATACATCCTTCTTTGACCCCGCAGGAGGAGGAGACCCCATTCTTTACCAACATCTCTTTNNNNNNNNNNNNNNN>CASENT0245122_Pheidole_spinosaNNNGCTTTACTTTATCTTTGCAATCTGATCTGGTATAATTGGGTCCTCTATGAGATTAATTATTCGATTAGAACTCGGGTCCTGTAATTCCTTAATTAATAATGATCAAATTTATAATTCATTAGTTACAAGACACGCTTTCATTATAATCTTTTTTATAGTAATACCCTTCATAATTGGAGGATTTGGTAATTTCCTAGTCCCTTTAATACTTGGCTCTCCAGATATAGCCTACCCACGAATAAATAATATAAGATTCTGACTCCTCCCACCTTCTATTACTCTTCTCTTACTAGGAAGATTCATTAATTCAGGAGCCGGAACAGGATGAACAGTTTACCCCCCTCTAGCCTCTAATATTTTTCATAGAGGAGCCTCTATTGACCTCTCAATTTTCTCATTACATATTGCAGGAATGTCTTCCATTCTTGGAGCTATTAATTTTATTTCTACAATTATTAATATACATCATAAAAATTTTACTATAGATAAAATTCCCTTATTAGTTTGATCAATTTTAATTACAGCAATTCTCCTTCTTCTCTCCCTCCCAGTCCTAGCAGGAGCAATTACCATACTTTTAACTGACCGTAACCTTAATACATCCTTCTTTGACCCCGCAGGAGGAGGAGACCCCATTCTTTACCAACATCTCTTNNNNNNNNNNNNNNNN>CASENT0245180_Pheidole_spinosaNNNCCTTTACTTTATCTTTGCAATCTGATCTGGTATAATTGGGTCCTCTATGAGATTAATTATTCGATTAGAACTCGGGTCCTGTAATTCCTTAATTAATAATGATCAAATTTATAATTCATTAGTTACAAGACACGCTTTCATTATAATCTTTTTTATAGTAATACCCTTCATAATTGGAGGATTTGGTAATTTCCTAGTCCCTTTAATACTTGGCTCTCCAGATATAGCCTACCCACGAATAAATAATATAAGATTCTGACTCCTCCCACCTTCTATTACTCTTCTCTTACTAGGAAGATTCATTAATTCAGGAGCCGGAACAGGATGAACAGTTTACCCCCCTCTAGCCTCTAATATTTTTCATAGAGGAGCCTCTATTGACCTCTCAATTTTCTCATTACATATTGCAGGAATGTCTTCCATTCTTGGAGCTATTAATTTTATTTCTACAATTATTAATATACATCATAAAAATTTTACTATAGATAAAATTCCCTTATTAGTTTGATCAATTTTAATTACAGCAATTCTCCTTCTTCTCTCCCTCCCAGTCCTAGCAGGAGCAATTACCATACTTTTAACTGACCGTAACCTTAATACATCCTTCTTTGACCCCGCAGGAGGAGGAGACCCCATTCTTTACCAACATCTCTTTTNNNNNNNNNNNNNN>CASENT0043338_Pheidole_spinosaGGTACTTTACTTTATCTTTGCAATCTGATCTGGAATAATTGGGTCCTCTATGAGATTAATTATTCGATTAGAACTCGGATCCTGTAACTCCTTAATTAATAATGATCAAATTTATAATTCATTAGTTACAAGACACGCTTTCATTATAATTTTTTTTATAGTAATACCTTTCATAATTGGAGGATTTGGTAATTTCCTGGTCCCTTTAATACTTGGCTCTCCAGATATAGCCTACCCACGAATAAATAATATAAGTTTCTGACTCCTCCCACCTTCCATCACTCTTCTTTTACTAGGAAGATTCATTAATTCAGGGGCCGGAACAGGATGAACAGTTTACCCCCCTCTAGCCTCTAATATTTTTCATAGAGGAGCCTCTATTGACCTCTCAATTTTTTCCTTACATATTGCAGGAATATCTTCCATTCTTGGAGCTATTAATTTTATCTCTACAATTATTAATATACATCATAAAAATTTTACTATAGATAAAATTCCTTTATTAGTTTGATCAATTTTAATTACAGCAATTCTCCTTCTTCTCTCCCTCCCCGTCCTCGCAGGAGCAATTACTATACTTTTAACTGACCGTAACCTTAATACATCCTTCTTTGACCCCGCAGGAGGAGGAGACCCCATTCTTTACCAACATCTCTTTNNNNNNNNNNNNNNN>CASENT0232626_Pheidole_spinosaTGTACTTTACTTTATCTTTGCAATCTGATCTGGAATAATTGGGTCCTCTATGAGATTAATTATTCGATTAGAACTCGGATCCTGTAACTCCTTAATTAATAATGATCAAATTTATAATTCATTAGTTACAAGACACGCTTTCATTATAATCTTTTTTATAGTAATACCTTTCATAATTGGAGGATTTGGTAATTTCCTAGTCCCTTTAATACTTGGCTCTCCAGATATAGCCTACCCACGAATAAATAATATAAGATTCTGACTCCTCCCACCTTCTATTACTCTTCTCTTATTAGGNAGATTCATTAATTCAGGGGCCGGAACTGGATGAACAGTTTACCCCCCTCTAGCCTCTAATATTTTTCATAGAGGAGCCTCTATTGACCTCTCAATTTTCTCCTTACATATTGCAGGAATGTCTTCCATCCTTGGGGCTATTAATTTTATCTCTACAATTATTAATATACATCATAAAAATTTTACTATAGATAAAATTCCTTTATTAGTTTGATCAATTTTAATTACAGCAATTCTCCTTCTTCTCTCCCTCCCAGTCCTCGCGGGAGCAATTACAATACTTTTAACTGACCGTAACCTTAATACATCCTTCTTTGACCCCGCAGGAGGAGGAGACCCCATTCTTTACCAACATCTCTTTNNNNNNNNNNNNNNN>CASENT0198045_Pheidole_spinosaTGTACTTTACTTTATCTTTGCAATCTGATCTGGAATAATTGGGTCCTCTATGAGATTAATTATTCGATTAGAACTCGGATCCTGTAACTCCTTAATTAATAATGATCAAATTTATAATTCATTAGTTACAAGACACGCTTTCATTATAATCTTTTTTATAGTAATACCTTTTATAATTGGAGGATTTGGTAATTTCCTAGTCCCCTTAATACTTGGTTCTCCAGATATAGCCTACCCACGAATAAATAATATAAGATTCTGACTCCTCCCACCTTCTATCACTCTTCTATTACTAGGAAGATTCATTAATTCAGGGGCTGGAACAGGGTGAACAGTTTATCCCCCTCTAGCCTCTAATATTTTTCATAGAGGGGCCTCTATCGACCTCTCAATTTTCTCCTTACATATTGCAGGAATGTCTTCCATTCTTGGAGCTATTAATTTTATCTCTACAATTATTAATATACATCATAAAAATTTTACTATAGATAAAATTCCTTTATTAGTTTGATCAATTTTAATTACAGCAATTCTCCTTCTTCTCTCCCTCCCAGTCCTCGCAGGAGCAATTACCATACTTTTAACTGACCGTAACCTTAATACATCCTTCTTTGACCCAGCAGGAGGAGGAGACCCCATTCTTTACCAACATCTCTTTNNNNNNNNNNNNNNN>CASENT0110610_Pheidole_spinosaGGTACTTTACTTTATCTTTGCAATCTGATCTGGAATAATTGGGTCCTCTATGAGATTAATTATTCGATTAGAACTCGGATCCTGTAACTCCTTAATTAATAATGATCAAATTTATAATTCATTAGTTACAAGACACGCTTTCATTATAATCTTTTTTATAGTAATACCTTTCATAATTGGAGGATTTGGTAATTTCCTAGTCCCCTTAATACTTGGTTCTCCAGATATAGCCTACCCACGAATAAATAATATAAGATTCTGACTCCTCCCACCTTCTATCACTCTTCTATTACTAGGAAGATTCATTAATTCAGGGGCTGGAACAGGGTGAACAGTTTATCCCCCTCTAGCCTCTAATATTTTTCATAGAGGGGCCTCTATCGACCTCTCAATTTTCTCCTTACATATTGCAGGAATGTCTTCCATTCTTGGAGCTATTAATTTTATCTCTACAATTATTAATATACATCATAAAAATTTTACTATAGATAAAATTCCTTTATTAGTTTGATCAATTTTAATTACAGCAATTCTCCTTCTTCTCTCCCTCCCAGTCCTCGCAGGAGCAATTACCATACTTTTAACTGACCGTAACCTTAATACATCCTTCTTTGACCCAGCAGGGGGAGGAGACCCCATTCTTTACCAACATCTCTTTNNNNNNNNNNNNNNN>CASENT0048398_Pheidole_spinosaAGTACTTTACTTTATCTTTGCAATCTGATCTGGAATAATTGGGTCCTCTATGAGATTAATTATTCGATTAGAACTCGGATCCTGTAACTCCTTAATTAATAATGATCAAATTTATAATTCATTAGTTACAAGACACGCTTTCATTATAATCTTTTTTATAGTAATACCTTTCATAATTGGAGGATTTGGTAATTTCCTAGTCCCCTTAATACTTGGTTCTCCAGATATAGCCTACCCACGAATAAATAATATAAGATTCTGACTCCTCCCACCTTCTATCACTCTTCTATTACTAGGAAGATTCATTAATTCAGGGGCTGGAACAGGGTGAACAGTTTATCCCCCTCTAGCCTCTAATATTTTTCATAGAGGGGCCTCTATCGACCTCTCAATTTTCTCCTTACATATTGCAGGAATGTCTTCCATTCTTGGAGCTATTAATTTTATCTCTACAATTATTAATATACATCATAAAAATTTTACTATAGATAAAATTCCTTTATTAGTTTGATCAATTTTAATTACAGCAATTCTCCTTCTTCTCTCCCTCCCAGTCCTCGCAGGAGCAATTACCATACTTTTAACTGACCGTAACCTTAATACATCCTTCTTTGACCCAGCAGGAGGAGGAGACCCCATTCTTTACCAACATCTCTTTNNNNNNNNNNNNNNN>CASENT0147025_Pheidole_spinosaTGTACTTTACTTTATCTTTGCAATCTGATCTGGAATAATTGGGTCCTCTATGAGATTAATTATTCGATTAGAACTCGGATCCTGTAATTCCTTAATTAATAATGATCAAATTTATAATTCATTAGTTACAAGACACGCTTTCATTATAATCTTTTTTATAGTAATACCTTTCATAATTGGAGGATTTGGTAATTTCCTAGTCCCCTTAATACTTGGTTCCCCAGATATAGCCTACCCACGAATAAATAATATAAGATTCTGACTCCTCCCACCTTCTATCACTCTTCTATTACTAGGAAGATTCATTAATTCAGGAGCTGGAACAGGGTGAACAGTTTACCCCCCTCTAGCCTCTAATATTTTTCATAGAGGAGCCTCTATCGACCTCTCAATTTTCTCCTTACATATTGCAGGAATGTCTTCCATTCTTGGAGCTATTAATTTTATCTCTACAATTATTAATATACATCATAAAAATTTTACTATAGATAAAATTCCTTTATTAGTTTGATCAATTTTAATTACAGCAATTCTCCTTCTTCTCTCCCTCCCAGTCCTCGCTGGAGCAATTACCATACTTTTAACTGACCGTAACCTTAATACATCCTTCTTTGACCCAGCAGGAGGAGGCGACCCCATTCTTTACCAACATCTCTTTNNNNNNNNNNNNNNN>CASENT0148841_Pheidole_spinosaNNNNNNNNNNNNNNNNNNNNNNNNNNNNNNNNNNNNNNNNNNNNNNNNNNNNNNNNNNNNNNNNNNNNNNNAACTCGGATCCTGTAACTCCTTAATTAATAATGATCAAATTTATAATTCATTAGTTACAAGACACGCTTTCATTATAATCTTTTTTATAGTAATACCTTTCATAATTGGAGGATTTGGTAATTTCCTAGTCCCCTTAATACTTGGTTCTCCAGATATAGCTTACCCACGAATAAATAATATAAGATTCTGACTCCTCCCACCTTCTATCACTCTTCTATTACTAGGAAGATTCATTAATTCAGGAGCTGGAACAGGGTGAACAGTTTATCCCCCTCTAGCCTCTAATATTTTTCATAGAGGAGCCTCTATCGACCTCTCAATTTTCTCCTTACATATTGCAGGAATGTCTTCCATTCTTGGAGCTATTAATTTTATCTCTACAATTATTAATATACATCATAAAAATTTTACTATAGATAAAATTCCTTTATTAGTTTGATCAATTTTAATTACAGCAATTCTCCTTCTTCTCTCCCTCCCAGTCCTCGCTGGAGCAATTACCATACTTTTAACTGACCGTAACCTTNNNNNNNNNNNNNNNNNNNNNNNNNNNNNNNNNNNNNNNNNNNNNNNNNNNNNNNNNNNNNNNNNNNNNNNNNNN>CASENT0236203_Pheidole_spinosaNNNNCTTTACTTTATCTTTGCAATCTGATCTGGAATAATTGGATCCTCTATGAGATTAATTATTCGTTTAGAACTCGGATCCTGTAACTCCTTAATTAATAATGATCAAATTTATAATTCATTAGTTACAAGACACGCTTTCATTATAATCTTTTTTATAGTAATACCTTTCATAATTGGAGGATTTGGAAATTTCCTAGTCCCTTTAATACTTGGCTCTCCAGACATAGCCTACCCACGAATAAATAACATAAGATTCTGACTCCTCCCTCCTTCTATTACTCTTCTATTGCTAGGAAGATTCATTAATTCAGGAGCTGGAACAGGATGAACAGTTTACCCACCTCTAGCCTCTAATATTTTTCATAGAGGAGCCTCTATTGACCTCTCAATTTTCTCCTTACATATTGCAGGAATGTCTTCCATTCTTGGAGCTATTAATTTTATCTCTACAATTATTAATATACATCATAAAAATTTTACTATAGATAAAATTCCTTTATTAGTTTGATCAATTTTAATTACAGCAATTCTACTTCTTCTCTCCCTCCCAGTCCTCGCAGGAGCAATTACCATACTTTTAACTGACCGTAACCTTAATACATCCTTCTTTGACCCAGCAGGAGGAGGAGACCCCATTCTTTACCAACATCTCTTNNNNNNNNNNNNNNNN>CASENT0490606_Pheidole_spinosaNNNNNNNNNNNNNNNNNNNNNNNNNNNNNNNGGAATAATTGGATCCTCCATGAGATTAATTATTCGGTTAGAACTCGGATCCTGTAACTCCTTAATTAATAATGATCAAATTTATAATTCATTAGTTACAAGACACGCTTTCATTATAATCTTTTTTATAGTAATACCTTTCATAATTGGAGGATTTGGTAATTTCCTGGTCCCTTTAATACTCGGCTCTCCAGATATAGCCTACCCACGAATAAATAACATAAGATTCTGACTCCTTCCTCCTTCTATCACTCTTCTATTACTAGGAAGATTCATTAATTCAGGAGCTGGAACTGGATGAACTNNNNNNNNNNNNNNNNNNNNNNNNNNNNNNNNNNNNNNNNNNNNNNNNNNNNNNNNNNNNNNNNNNNNNNNNNNNNNNNNNNNNNNNNNNNNNNNNNNNNNNNNNNNNNNNNNNNNNNNNNNNNNNNNNNNNNNNNNNNNNNNNNNNNNNNNNNNNNNNNNNNNNNNNNNNNNNNNNNNNNNNNNNNNNNNNNNNNNNNNNNNNNNNNNNNNNNNNNNNNNNNNNNNNNNNNNNNNNNNNNNNNNNNNNNNNNNNNNNNNNNNNNNNNNNNNNNNNNNNNNNNNNNNNNNNNNNNNNNNNNNNNNNNNNNNNNNNNNNNNNNNNNNNNNNNNNNNNNNN>CASENT0125300_Pheidole_spinosaTATACTTTACTTTATCTTTGCAATCTGATCTGGAATAATTGGATCCTCCATGAGATTAATTATTCGGTTAGAACTCGGATCCTGTAACTCCTTAATTAATAATGATCAAATTTATAATTCATTAGTTACAAGACACGCTTTCATTATAATCTTCTTTATAGTAATACCTTTCATGATTGGAGGATTTGGTAATTTCCTAGTCCCTTTAATACTTGGCTCTCCAGATATAGCCTACCCACGAATAAATAACATAAGATTCTGACTCCTTCCTCCTTCTATCACTCTTCTATTACTAGGAAGATTCATTAATTCAGGGGCTGGAACAGGATGAACAGTTTACCCCCCTCTAGCCTCTAATATTTTTCATAGAGGAGCCTCTATTGACCTCTCAATTTTCTCCTTACATATTGCAGGAATGTCTTCCATTCTTGGAGCTATTAATTTTATCTCTACAATTATTAATATACATCATAAAAATTTTACTATAGATAAAATTCCTTTATTAGTTTGATCAATTTTAATTACAGCAATTCTACTTCTTCTCTCCCTACCAGTTCTCGCAGGAGCAATTACCATACTTTTAACTGACCGTAACCTTAATACATCCTTCTTTGATCCGGCAGGAGGAGGAGACCCCATTCTTTACCAACATCTCTTTNNNNNNNNNNNNNNN>CASENT0484817_Pheidole_spinosaTATACTTTACTTTATCTTTGCAATCTGATCTGGAATAATTGGATCCTCCATGAGATTAATTATTCGGTTAGAACTCGGATCCTGTAACTCCTTAATTAATAATGATCAAATTTATAATTCCTTAGTTACAAGACACGCTTTCATTATAATCTTTTTTATAGTAATACCTTTCATAATTGGAGGATTTGGTAATTTCCTAGTCCCTTTAATACTTGGCTCTCCAGATATAGCCTACCCACGAATAAATAACATAAGATTCTGACTCCTTCCTCCTTCTATCACTCTTCTATTACTAGGAAGATTCATTAATTCAGGAGCTGGAACAGGATGAACAGTTTACCCCCCTCTAGCCTCTAATATTTTTCATAGAGGAGCCTCTATTGACCTCTCAATTTTCTCCTTACATATTGCAGGAATGTCTTCCATTCTTGGAGCTATTAATTTTATCTCTACAATTATTAATATACATCATAAAAATTTTACTATAGATAAAATTCCTTTATTAGTATGATCAATTTTAATTACAGCAATTCTACTTCTTCTCTCCCTACCAGTTCTCGCAGGAGCAATTACCATACTTTTAACTGACCGTAACCTTAATACATCCTTCTTTGACCCAGCAGGAGGAGGAGACCCCATTCTTTACCAACATCTCTTTNNNNNNNNNNNNNNN>CASENT0484833_Pheidole_spinosaTATACTTTACTTTATCTTTGCAATCTGATCTGGAATAATTGGATCCTCCATGAGATTAATTATTCGGTTAGAACTCGGATCCTGTAACTCCTTAATTAATAATGATCAAATTTATAATTCATTAGTTACAAGACACGCTTTCATTATAATCTTTTTTATAGTAATACCTTTCATAATTGGAGGATTTGGTAATTTCCTAGTCCCTTTAATACTTGGCTCTCCAGATATAGCCTACCCACGAATAAATAACATAAGATTCTGACTCCTTCCTCCTTCTATCACTCTTCTATTACTAGGAAGATTCATTAATTCAGGAGCTGGAACAGGATGAACAGTTTACCCCCCTCTAGCCTCTAATATTTTTCATAGAGGAGCCTCTATTGACCTCTCAATTTTCTCCTTACATATTGCAGGAATGTCTTCCATTCTTGGAGCTATTAATTTTATCTCTACAATTATTAATATACATCATAAAAATTTTACTATAGATAAAATTCCTTTATTAGTATGATCAATTTTAATTACAGCAATTCTACTTCTTCTCTCCCTACCAGTTCTCGCAGGAGCAATTACCATACTTTTAACTGACCGTAACCTTAATACATCCTTCTTTGATCCAGCAGGAGGAGGAGACCCCATTCTTTACCAACATCTCTTTNNNNNNNNNNNNNNN>CASENT0488836_Pheidole_spinosaTATACTTTACTTTATCTTTGCAATCTGATCTGGAATAATTGGATCCTCTATGAGATTAATTATTCGTTTAGAACTCGGATCCTGTAACTCCTTAATTAATAATGATCAAATTTATAACTCATTAGTGACAAGACACGCTTTCATTATAATCTTTTTTATAGTAATACCTTTCATAATTGGAGGATTTGGTAATTTCCTAGTCCCTTTAATACTCGGCTCTCCAGATATAGCCTACCCCCGAATAAATAACATAAGATTCTGACTCCTTCCTCCTTCTATCACTCTTCTATTGCTAGGAAGATTCATTAATTCAGGAGCTGGAACAGGATGAACAGTTTACCCACCTCTAGCCTCTAATATTTTTCATAGAGGAGCCTCTATTGACCTCTCAATTTTCTCCTTACATATTGCAGGAATGTCATCCATTCTTGGAGCTATTAATTTTATTTCTACAATTATTAATATACATCATAAAAATTTTACTATAGATAAAATTCCATTATTAGTTTGATCAATTTTAATTACAGCAATTCTACTTCTTCTCTCCCTTCCAGTCCTTGCAGGAGCAATTACCATACTTTTAACTGACCGTAACCTTAATACATCCTTCTTTGACCCAGCAGGAGGGGGAGACCCCATTCTTTACCAACATCTCTTTNNNNNNNNNNNNNNN>CASENT0060284_Pheidole_spinosaNATACTTTACTTTATCTTTGCAATCTGATCTGGAATAATTGGATCCTCTATAAGATTAATTATTCGATTAGAACTCGGATCCTGTAACTCCTTAATTAATAATGATCAAATTTATAATTCATTAGTTACAAGACACGCTTTCATTATAATCTTTTTTATAGTAATACCTTTCATAATTGGAGGATTTGGTAATTTCCTAGTACCTTTAATACTCGGCTCTCCAGATATAGCCTACCCACGAATAAATAACATAAGATTCTGACTCCTTCCCCCTTCTATCACTCTTCTATTACTAGGAAGATTCATTAATTCAGGAGCTGGAACAGGATGAACAGTTTACCCCCCGCTAGCCTCTAATATTTTTCATAGAGGAGCCTCTATTGATCTCTCAATTTTCTCCTTACATATTGCAGGAATGTCTTCCATTCTTGGAGCTATTAATTTTATTTCTACAATTATTAATATACATCATAAAAATTTTACTATAGATAAAATTCCTTTATTAGTTTGATCAATTTTAATTACAGCAATTCTACTTCTTCTCTCCCTACCAGTCCTTGCAGGAGCAATTACCATACTTTTAACTGACCGTAACCTTAATACATCCTTCTTTGACCCAGCAGGAGGGGGAGACCCCATTCTTTACCAACATCTCNNNNNNNNNNNNNNNNNN>CASENT0065558_Pheidole_spinosaNNNNCTTTACTTTATCTTTGCAATCTGATCTGGAATAATTGGATCCTCTATGAGATTAATTATTCGATTAGAACTCGGATCCTGTAACTCCTTAATTAATAATGATCAAATTTATAATTCATTAGTTACAAGACACGCTTTCATTATAATCTTTTTTATAGTTATACCTTTCATAATTGGAGGATTTGGTAATTTCCTAGTCCCTTTAATACTAGGCTCTCCAGATATAGCCTACCCACGAATAAATAACATAAGATTCTGACTCCTTCCTCCTTCTCTCACTCTTCTTTTACTAGGAAGATTCATTAATTCAGGAGCTGGAACAGGATGAACAGTTTACCCCCCTCTAGCCTCTAATATTTTTCATAGAGGAGCCTCTATTGATCTCTCAATTTTCTCCTTACATATTGCAGGAATGTCTTCCATTCTTGGAGCTATTAATTTTATCTCTACAATTATTAATATACATCATAAAAATTTTACTATAGATAAAATTCCTTTATTAGTTTGATCAATTTTAATTACAGCAATTCTACTTCTTCTCTCCCTCCCAGTTCTAGCAGGAGCAATTACCATACTTTTAACTGACCGTAACCTTAACACATCCTTCTTTGACCCAGCAGGAGGAGGAGATCCCATTCTTTACCAACATCTCNNNNNNNNNNNNNNNNNN>CASENT0149829_Pheidole_spinosaTATACTTTACTTTATCTTTGCAATCTGATCTGGAATAATTGGATCCTCTATGAGATTAATTATTCGATTAGAACTCGGATCCTGTAACTCCTTAATTAATAATGATCAAATTTATAATTCATTAGTTACAAGACACGCTTTCATTATAATCTTTTTTATAGTTATACCTTTCATAATTGGAGGATTTGGTAATTTCCTAGTCCCTTTAATACTCGGCTCTCCAGATATAGCCTACCCACGAATAAATAACATAAGATTCTGACTCCTTCCTCCTTCTATCACTCTTCTTTTACTAGGAAGATTCATTAATTCAGGAGCTGGAACAGGATGAACAGTTTACCCCCCTCTAGCCTCTAATATTTTTCATAGAGGAGCCTCTATTGATCTCTCAATTTTTTCCTTACATATTGCAGGAATGTCTTCCATTCTTGGAGCTATTAATTTTATCTCTACAATTATTAATATACATCATAAAAATTTTACTATAGATAAAATTCCTTTATTAGTTTGATCAATTTTAATTACAGCAATTCTACTTCTTCTCTCCCTCCCAGTTCTCGCAGGAGCAATTACCATACTTTTAACTGACCGTAACCTTAATACATCCTTCTTTGACCCAGCAGGAGGAGGAGACCCCATTCTTTACCAACATCTCTTTNNNNNNNNNNNNNNN>CASENT0418283_Pheidole_spinosaTATACTTTACTTTATCTTTGCAATCTGATCTGGTATAATTGGATCCTCTATGAGATTAATTATTCGATTAGAACTCGGATCCTGTAACTCCTTAATTAATAATGATCAAATTTATAATTCATTAGTTACAAGACACGCTTTCATTATAATCTTTTTTATAGTTATACCTTTCATAATTGGAGGATTTGGTAATTTCCTAGTCCCTTTAATACTCGGCTCTCCAGATATAGCCTACCCACGAATAAATAACATAAGATTCTGACTCCTTCCTCCTTCTATCACTCTTCTTTTACTAGGAAGATTCATTAATTCAGGAGCTGGAACAGGATGAACAGTTTACCCCCCTCTAGCCTCTAATATTTTTCATAGAGGAGCCTCTATTGATCTCTCAATTTTCTCCTTACATATTGCAGGAATGTCTTCCATTCTTGGAGCTATTAATTTTATCTCTACAATTATTAATATACATCATAAAAATTTTACTATAGATAAAATTCCTTTATTAGTTTGATCAATTTTAATTACAGCAATTCTACTTCTTCTCTCCCTCCCAGTTCTCGCAGGAGCAATCACCATACTTTTAACTGACCGTAACCTTAATACATCCTTCTTTGACCCAGCAGGAGGAGGAGACCCCATTCTTTACNNNNNNNNNNNNNNNNNNNNNNNNNNN>CASENT0206153_Pheidole_spinosaCGTACTTTACTTTATCTTTGCAATCTGATCTGGAATAATTGGATCTTCTATGAGATTAATTATTCGATTAGAACTAGGATCCTGTAACTCATTAATTAATAATGATCAAATTTATAATTCCTTAGTTACAAGACACGCTTTCATTATAATTTTCTTTATAGTAATGCCTTTCATAATTGGAGGATTTGGTAATTTCCTGGTCCCTTTAATACTTGGCTCCCCCGATATAGCTTACCCACGAATAAATAATATAAGATTCTGACTCCTCCCCCCTTCCATCACTCTCCTCTTATTAGGGAGATTCATTAATTCTGGGGCCGGAACAGGATGAACAGTTTACCCCCCTCTAGCCTCTAATATTTTTCATAGGGGGGCCTCTATCGACCTCTCAATTTTCTCCCTACATATTGCAGGGATATCTTCCATTCTTGGAGCTATTAATTTTATTTCTACAATCATTAATATACATCATAAAAATTTTACTATAGATAAAATTCCGTTATTAGTATGATCAATTTTAATTACAGCAATTCTCCTCCTTCTTTCCCTCCCAGTTCTTGCCGGAGCAATTACCATACTTTTAACCGACCGTAACCTTAATACATCCTTCTTTGACCCCGCAGGGGGAGGGGACCCCATCCTTTACCAACATCTCTTNNNNNNNNNNNNNNNN>CASENT0120056_Pheidole_spinosaNGTACTTTACTTTATCTTTGCAATCTGATCTGGAATAATTGGATCTTCTATGAGATTAATTATTCGATTAGAACTAGGATCCTGTAACTCATTAATTAATAATGATCAAATTTATAATTCCTTAGTTACAAGACACGCTTTCATTATAATTTTCTTTATAGTAATGCCTTTCATAATTGGGGGATTTGGTAATTTCCTGGTCCCTTTAATGCTTGGTTCCCCCGATATAGCTTACCCCCGAATAAATAATATAAGATTCTGACTCCTCCCCCCTTCCATCACTCTCCTCTTATTAGGGAGATTCATTAATTCTGGGGCCGGAACAGGATGAACAGTTTACCCCCCTCTAGCCTCTAATATTTTTCATAGGGGGGCCTCTATCGACCTCTCAATTTTCTCCCTACATATTGCAGGGATATCTTCCATTCTTGGGGCTATTAATTTTATCTCTACAATCATTAATATACATCATAAAAATTTTACTATAGATAAAATTCCGTTATTAGTATGATCAATTTTAATTACAGCAATTCTCCTCCTTCTTTCCCTCCCAGTTCTTGCCGGAGCAATTACCATACTTTTAACCGACCGTAATCTTAATACATCCTTCTTTGACCCCGCAGGAGGAGGGGACCCCATCCTTTACCAACATCTCNNNNNNNNNNNNNNNNNN>CASENT0213992_Pheidole_spinosaCGTACTTTACTTTATCTTTGCAATCTGATCTGGAATAATTGGATCTTCTATGAGATTAATTATTCGATTAGAACTAGGATCCTGTAACTCATTAATTAATAATGATCAAATTTATAATTCCTTAGTTACAAGACACGCTTTCATTATAATTTTCTTTATAGTAATGCCTTTCATAATTGGGGGATTTGGTAATTTCCTGGTCCCTTTAATGCTTGGTTCCCCCGATATAGCTTACCCCCGAATAAATAATATAAGATTCTGACTCCTCCCCCCTTCCATCACTCTCCTCTTATTAGGGAGATTCATTAATTCTGGGGCCGGAACAGGATGAACAGTTTACCCCCCTCTAGCCTCTAATATTTTTCATAGGGGGGCCTCTATCGACCTCTCAATTTTCTCCCTACATATTGCAGGAATATCTTCCATTCTTGGAGCTATTAATTTTATCTCTACAATCATTAATATACATCATAAAAATTTTACTATAGATAAAATTCCGTTATTAGTATGATCAATTTTAATTACAGCAATTCTCCTCCTTCTTTCCCTCCCAGTTCTTGCCGGAGCAATTACCATACTTTTAACCGACCGTAACCTTAATACATCCTTCTTTGACCCCGCAGGGGGAGGGGACCCCATCCTTTACCAACATCTCTTTNNNNNNNNNNNNNNN>CASENT0133216_Pheidole_decepticonNNNNNNNNNNNNNNNNNNNNNNNNNNNNNNNNNNNNAATTGGGTCTTCTATGAGATTAATTATTCGATTAGAACTAGGATCCTGCAATTCATTAATTAATAATGATCAAATTTATAACTCCTTAGTTACAAGACACGCTTTCATTATAATTTTCTTTATAGTAATACCTTTCATAATTGGGGGATTTGGTAATTTCCTGGTCCCTTTAATACTTGGCTCCCCAGATATAGCCTACCCACGAATAAATAATATAAGATTCTGACTCCTTCCCCCTTCCATTACTCTCCTCTTGTTAGGAAGATTCATTAATTCTGGCGCCGGAACNNNNNNNNNNNNNNNNNNNNNNNNNNNNNNNNNNNNNNNNNNNNNNNNNNNNNNNNNNNNNNNNNNNNNNNNNNNNNNNNNNNNNNNNNNNNNNNNNNNNNNNNNNNNNNNNNNNNNNNNNNNNNNNNNNNNNNNNNNNNNNNNNNNNNNNNNNNNNNNNNNNNNNNNNNNNNNNNNNNNNNNNNNNNNNNNNNNNNNNNNNNNNNNNNNNNNNNNNNNNNNNNNNNNNNNNNNNNNNNNNNNNNNNNNNNNNNNNNNNNNNNNNNNNNNNNNNNNNNNNNNNNNNNNNNNNNNNNNNNNNNNNNNNNNNNNNNNNNNNNNNNNNNNNNNNNNNNNNNNNNNNNNNNNN>CASENT0132558_Pheidole_decepticonNATACTTTACTTTATCTTTGCAATCTGATCTGGGATAATTGGGTCTTCTATGAGATTAATTATTCGATTAGAACTAGGATCCTGCAATTCATTAATTAATAATGATCAAATTTATAACTCCTTAGTTACAAGACACGCTTTCATTATAATTTTCTTTATAGTAATACCTTTCATAATTGGGGGATTTGGTAATTTCCTGGTCCCTTTAATACTTGGCTCCCCAGATATAGCCTACCCACGAATAAATAATATAAGATTCTGACTCCTTCCCCCTTCCATTACTCTCCTCTTGTTAGGAAGATTCATTAATTCTGGCGCCGGAACAGGATGAACAGTTTACCCCCCTCTAGCCTCTAATATTTTCCACAGAGGGGCCTCTATCGACCTCTCAATTTTCTCTCTACATATTGCAGGAATATCTTCCATTCTTGGGGCTATTAATTTTATTTCTACAATTATTAATATACATCATAAAAATTTTACTATAGATAAAATTCCTTTATTAGTGTGATCAATTTTAATCACAGCAATTCTCCTCCTCCTCTCCCTCCCAGTTCTTGCCGGAGCAATTACCATACTTTTAACCGACCGTAACCTTAATACATCCTTCTTTGACCCCGCAGGAGGGGGAGACCCCATCCTCTACCAACATCTCNNNNNNNNNNNNNNNNNN>CASENT0071552_Pheidole_spinosaCATACTTTACTTTATCTTTGCAATCTGATCTGGAATAATTGGATCTTCTATGAGATTAATTATTCGATTAGAACTAGGATCCTGTAACTCATTAATTAATAATGATCAAATTTATAACTCCTTAGTTACAAGACACGCTTTCATTATAATTTTCTTTATAGTAATGCCTTTCATAATTGGGGGATTTGGTAATTTCCTAGTCCCTTTAATACTTGGCTCCCCCGATATAGCCTACCCACGAATAAATAATATAAGATTCTGACTCCTCCCCCCTTCTATTACTCTCCTCTTGTTAGGAAGATTCATTAATTCTGGGGCCGGAACAGGGTGAACAGTTTACCCCCCTCTAGCCTCTAATATTTTTCATAGGGGGGCTTCTATCGACCTCTCAATTTTCTCCCTACATATTGCAGGAATATCTTCTATTCTTGGAGCTATTAATTTTATTTCTACAATCATTAATATACATCATAAAAATTTTACTATAGATAAAATTCCTCTATTAGTATGATCAATTTTAATTACAGCAATTCTCCTTCTTCTTTCCCTCCCAGTTCTTGCCGGAGCAATTACCATACTTTTAACCGACCGTAACCTTAATACATCCTTCTTTGACCCCGCAGGAGGAGGGGACCCAATTCTTTACCAACATCTCTTTNNNNNNNNNNNNNNN>CASENT0122716_Pheidole_spinosaNNNNNNNNACTTTATCTTTGCAATCTGATCTGGGATAATTGGATCTTCTATGAGATTAATTATTCGGTTAGAACTAGGATCCTGTAACTCATTAATTAATAATGATCAAATTTATAACTCCTTAGTTACAAGACACGCTTTCATTATAATTTTCTTTATAGTAATGCCTTTCATAATTGGGGGATTTGGTAATTTCCTAGTCCCTTTAATACTTGGCTCCCCCGATATAGCCTACCCACGAATAAATAATATAAGATTCTGACTCCTCCCCCCTTCCATTACTCTCCTCTTGTTAGGAAGATTCATTAATTCTGGGGCCGGGACAGGGTGAACAGTTTACCCCCCTCTAGCCTCTAATATTTTTCATAGGGGGGCTTCTATCGACCTCTCAATTTTCTCCCTACATATTGCAGGAATATCTTCCATTCTTGGGGCTATTAATTTTATTTCTACAATCATTAATATACATCATAAAAATTTTACTATAGATAAAATTCCTCTATTAGTATGATCAATTTTAATTACAGCAATTCTCCTTCTTCTCTCCCTCCCAGTTCTTGCCGGAGCAATTACCATACTTTTAACCGACCGTAATCTTAATACNNNNNNNNNNNNNNNNNNNNNNNNNNNNNNNNNNNNNNNNNNNNNNNNNNNNNNNNNNNNNNNNNNNNNN>CASENT0477194_Pheidole_spinosaNATACTTTACTTTATCTTTGCAATCTGATCTGGGATAATTGGATCTTCTATGAGATTAATTATTCGATTAGAACTAGGATCCTGTAACTCATTAATTAATAATGATCAAATTTATAACTCCTTAGTTACAAGACACGCTTTCATTATAATTTTCTTTATAGTAATGCCTTTCATAATTGGGGGATTTGGTAATTTCCTAGTCCCTTTAATACTTGGCTCCCCCGATATAGCCTACCCACGAATAAATAATATAAGATTCTGACTCCTCCCCCCTTCCATTACTCTCCTCTTGTTAGGAAGATTCATTAATTCTGGGGCCGGGACAGGGTGAACAGTTTACCCCCCTCTAGCCTCTAATATTTTTCATAGGGGGGCTTCTATCGACCTCTCAATTTTCTCCCTACATATTGCAGGAATATCTTCCATTCTTGGGGCTATTAATTTTATTTCTACAATCATTAATATACATCATAAAAATTTTACTATAGATAAAATTCCTCTATTAGTATGATCAATTTTAATTACAGCAATTCTCCTTCTTCTCTCCCTCCCAGTTCTTGCCGGAGCAATTACCATACTTTTAACCGACCGTAACCTTAATACATCCTTCTTTGACCCCGCAGGAGGAGGAGACCCAATTCTTTACCAACATCTCTTTNNNNNNNNNNNNNNN>CASENT0050816_Pheidole_spinosaNNNNNNNNNNNNNNNNNNNNNNNNNNNNNNNNNNNNNNNNNNNNNNNNNNNNNNNNTAATTATTCGATTAGAACTAGGATCCTGTAACTCATTAATTAATAATGATCAAATTTATAACTCCTTAGTTACAAGACACGCTTTCATTATAATTTTCTTTATAGTAATGCCTTTCATAATTGGGGGATTTGGTAATTTCCTAGTCCCTTTAATACTTGGCTCCCCCGATATAGCCTACCCACGAATAAATAATATAAGATTCTGACTCCTCCCCCCTTCCATTACTCTCCTCTTGTTAGGAAGATTCATTAATTCTGGGGCCGGGACAGGATGAACAGTTTACCCCCCTCTAGCCTCTAATATTTTTCATAGAGGGGCTTCTATCGACCTCTCAATTTTCTCCCTACATATTGCAGGAGTATCTTCCATTCTTGGGGCTATTAATTTTATTTCTACAATCATTAATATACATCATAAAAATTTTACTATAGATAAAATTCCTCTATTAGTATGATCAATTTTAATTACAGCAATTCTCCTTCTTCTCTCCCTCCCAGTTCTTGCCGGAGCAATTACCATACTTTTAACCGACCGTAACCTTAATACATCCTTCTNNNNNNNNNNNNNNNNNNNNNNNNNNNNNNNNNNNNNNNNNNNNNNNNNNNNNNNNNNNNNN>CASENT0051814_Pheidole_spinosaCATACTTTACTTTATCTTTGCAATCTGATCTGGGATAATTGGATCTTCTATGAGATTAATTATTCGATTAGAACTAGGATCCTGTAACTCATTAATTAATAATGATCAAATTTATAACTCCTTAGTTACAAGACACGCTTTCATTATAATTTTCTTTATAGTAATGCCTTTCATAATTGGGGGATTTGGTAATTTCCTAGTCCCTTTAATACTTGGCTCCCCCGATATAGCCTACCCACGAATAAATAATATAAGATTCTGACTCCTCCCCCCTTCCATTACTCTCCTCTTGTTAGGAGGATTCATTAATTCTGGGGCCGGGACAGGATGAACAGTTTACCCCCCTCTAGCCTCTAATATTTTTCATAGAGGGGCTTCTATCGACCTCTCAATTTTCTCCCTACATATTGCAGGAATATCTTCCATTCTTGGGGCTATTAATTTTATTTCTACAATCATTAATATACATCATAAAAATTTTACTATAGATAAAATTCCTCTATTAGTATGATCAATTTTAATTACAGCAATTCTCCTTCTTCTCTCCCTCCCAGTTCTTGCCGGAGCAATTACCATACTTTTAACCGACCGTAACCTTAATACATCCTTCTTTGACCCCGCAGGAGGAGGGGACCCAATTCTTTACCAACATCTCTTTNNNNNNNNNNNNNNN>CASENT0107891_Pheidole_spinosaTATACTATACTTTATCTTTGCAATCTGATCTGGAATAATTGGATCCTCTATGAGATTAATTATTCGATTAGAACTTGGATCCTGTAACTCCTTAATTAATAATGATCAAATTTATAACTCTTTAGTTACAAGGCATGCTTTCATTATAATTTTCTTTATAGTAATACCTTTCATAATTGGAGGATTTGGTAATTTCCTGGTCCCTTTAATACTTGGTTCCCCAGATATAGCCTACCCACGAATAAATAATATGAGATTCTGACTACTCCCTCCTTCCATTACTCTCCTCTTATTAGGGAGGTTTATTAATTCAGGAGCCGGAACTGGATGGACAGTTTACCCCCCCCTAGCCTCTAATATTTTCCATAGGGGAGCCTCTATTGACCTCTCAATTTTCTCCCTACATATTGCAGGAATGTCTTCCATTCTTGGGGCTATCAATTTTATTGCTACAATTATTAATATACATCATAAAAATTTTACTATAGATAAAATTCCCTTATTAGTTTGATCAATTTTAATTACGGCAATTCTGCTTCTTCTCTCCCTACCTGTCCTCGCTGGAGCAATCACCATACTTTTAACCGACCGTAACCTTAATACATCCTTCTTTGACCCAGCAGGAGGGGGGGACCCCATTCTTTACCAACACCTCTTTNNNNNNNNNNNNNNN>CASENT0041445_Pheidole_spinosaTATACTATACTTTATCTTTGCAATCTGATCTGGAATAATTGGATCCTCTATGAGATTAATTATTCGATTAGAACTTGGATCCTGTAACTCCTTAATTAATAATGATCAAATTTATAACTCTTTAGTTACAAGACATGCTTTCATTATAATTTTCTTTATAGTAATACCTTTCATAATTGGGGGATTTGGTAATTTCCTAGTCCCTTTAATACTTGGTTCCCCAGATATAGCCTACCCACGAATAAATAATATGAGATTCTGACTACTCCCTCCTTCCATTACTCTCCTCTTATTAGGGAGGTTTATTAATTCAGGAGCCGGAACTGGATGGACAGTTTACCCCCCCCTAGCCTCTAATATTTTCCATAGGGGGGCCTCTATTGACCTCTCAATTTTCTCCCTACATATTGCAGGGATGTCTTCCATTCTTGGAGCTATCAATTTTATTGCTACAATTATTAATATACATCATAAAAATTTTACTATAGATAAAATTCCCTTATTAGTTTGATCAATTTTAATTACAGCAATTCTGCTTCTTCTCTCCCTCCCTGTCCTCGCCGGAGCAATCACCATACTTTTAACCGACCGTAACCTTAATACATCCTTCTTTGACCCAGCAGGAGGGGGGGACCCCATTCTTTACCAACACCTCTTTNNNNNNNNNNNNNNN>CASENT0044509_Pheidole_spinosaNNNNNNNNNNNNNNNNNNNNNNNNNNNNNNNNNNNNNNNTGGATCCTCTATGAGATTAATTATTCGATTAGAACTTGGATCCTGTAACTCCTTAATTAATAATGATCAAATTTATAACTCTTTAGTTACAAGACATGCTTTCATTATAATTTTCTTTATAGTAATACCTTTCATAATTGGGGGATTTGGTAATTTCCTAGTCCCTTTAATACTTGGTTCTCCAGATATAGCCTACCCACGAATAAATAATATGAGATTCTGACTACTCCCTCCTTCCATTACTCTCCTCTTATTAGGTAGATTTATTAATTCAGGAGCCGGAACTGGATGGACAGTTTACCCCCCCCTAGCCTCTAATATTTTCCATAGGGGGGCCTCTATTGACCTCTCAATTTTCTCCCTACATATTGCAGGTATGTCTTCCATTCTTGGAGCTATCAATTTTATTGCTACAATTATTAATATACATCATAAAAATTTTACTATAGATAAAATTCCCTTATTAGTTTGATCAATTTTAATTACAGCAATTCTGCTTCTTCTCTCCCTCCCTGTCCTCGCCGGAGCAATCACCATACTTTTAACCGACCGTAACCTTAATACATCCTTCTTTGACCCAGCAGGAGGGGGGGATCCCATTCTTTACCAACACCTCTTTNNNNNNNNNNNNNNN>CASENT0071457_Pheidole_spinosaTATACTCTACTTTATCTTTGCAATCTGATCTGGAATAATTGGATCCTCTATGAGATTAATTATTCGATTAGAACTTGGATCCTGCAACTCCTTAATTAATAATGATCAAATTTATAACTCTTTAGTTACAAGACACGCTTTCATTATAATTTTCTTTATAGTAATACCTTTCATAATTGGGGGATTTGGTAATTTCCTAGTCCCTTTAATACTTGGTTCCCCAGATATAGCCTACCCACGAATAAATAATATGAGATTCTGACTACTCCCCCCCTCCATTACTCTCCTCTTATTAGGAAGATTAATTAATTCAGGTGCCGGAACTGGATGAACAGTTTATCCCCCCTTAGCCTCTAATATTTTCCATAGGGGGGCCTCTATTGACCTCTCAATTTTCTCCCTACATATTGCAGGAATATCTTCAATCCTTGGGGCTATTAATTTTATTGCCACAATTATTAATATACATCATAAAAATTTTACTATAGATAAAATTCCTTTACTAGTTTGATCAATCTTAATTACAGCAATTCTCCTTCTTCTCTCTCTCCCAGTTCTCGCGGGAGCAATTACCATACTTTTAACTGACCGTAACCTTAATACATCCTTCTTTGACCCCGCAGGAGGAGGAGACCCCATTCTTTACCAACATCTCTTTNNNNNNNNNNNNNNN>CASENT0031136_Pheidole_spinosaNNNNNNNNNNNNNNNNNNNNNNNNNNNNNNNNNNNNNNNNNNNNNNNCTATGAGATTAATTATTCGATTAGAACTCGGATCCTGCAACTCCTTAATTAATAATGATCAAATTTATAACTCTTTAGTTACAAGACACGCTTTCATTATAATTTTCTTTATAGTAATACCTTTCATAATTGGGGGATTTGGTAATTTCCTAGTCCCTTTAATACTTGGTTCCCCAGATATAGCCTACCCACGAATAAATAATATGAGATTCTGACTACTTCCCCCCTCCATTACTCTCCTCTTATTAGGAAGATTAATTAATTCAGGTGCCGGAACTGGATGAACAGTTTATCCCCCCTTAGCCTCTAATGTTTTCCATAGGGGGGCCTCTATTGACCTCTCAATTTTCTCCCTACATATTGCAGGAATATCTTCAATCCTTGGGGCTATTAATTTTATTGCCACAATTATTAATATACATCATAAAAATTTTACTATAGATAAAATTCCTTTACTAGTTTGATCAATTTTAATTACAGCAATTCTCCTTCTTCTCTCCCTCCCAGTTCTCGCTGGAGCAATTACCATACTTTTAACTGACCGTAACCTTAATACATCCTTCTTTGACCCCGCAGGAGGAGGAGACCCCATTCTTTACCAACATCTCTTTNNNNNNNNNNNNNNN>CASENT0492583_Pheidole_spinosaTATACTCTACTTTATCTTTGCAATCTGATCTGGAATAATTGGATCCTCTATGAGATTAATTATTCGATTAGAACTCGGATCCTGCAGCTCCTTAATTAATAATGATCAAATTTATAACTCTTTAGTTACAAGACACGCTTTCATTATAATTTTCTTTATAGTAATACCTTTCATAATTGGGGGATTTGGTAATTTCCTAGTCCCTTTAATACTTGGTTCCCCAGATATAGCCTACCCACGAATAAATAATATGAGATTCTGACTACTTCCCCCCTCCATTACTCTCCTCTTATTAGGAAGATTAATTAATTCAGGTGCCGGAACTGGATGAACAGTTTATCCCCCCTTAGCCTCTAATGTTTTCCATAGGGGGGCCTCTATTGACCTCTCAATTTTCTCCCTACATATTGCAGGAATATCTTCAATCCTTGGGGCTATTAATTTTATTGCCACAATTATTAATATACATCATAAAAATTTTACTATAGATAAAATTCCTTTACTAGTTTGATCAATTTTAATTACAGCAATTCTCCTTCTTCTCTCCCTCCCAGTTCTCGCTGGAGCAATTACCATACTTTTAACTGACCGTAACCTTAATACATCCTTCTTTGACCCCGCAGGAGGAGGAGACCCCATTCTTTACCAACATCTCTTTNNNNNNNNNNNNNNN>CASENT0022664_Pheidole_spinosaTATACTCTACTTTATCTTTGCAATCTGATCTGGAATAATTGGATCCTCTATGAGATTAATTATTCGATTAGAACTCGGATCCTGCAACTCCTTAATTAATAATGATCAAATTTATAACTCTTTAGTTACAAGACACGCTTTCATTATAATTTTCTTTATAGTAATACCTTTCATAATTGGAGGATTTGGTAATTTCCTAGTCCCTTTAATACTTGGTTCCCCAGATATAGCCTACCCACGAATAAATAATATGAGATTCTGACTACTTCCCCCCTCCATTACTCTCCTCTTATTAGGAAGATTAATTAATTCAGGTGCCGGAACTGGATGAACAGTTTACCCCCCCTTAGCCTCTAATATTTTCCATAGGGGAGCCTCTATTGACCTTTCAATTTTCTCCCTACATATTGCAGGAATATCTTCCATCCTTGGGGCTATTAATTTTATTGCCACGATTATTAATATACATCATAAAAATTTCACTATAGATAAAATTCCTTTATTAGTTTGATCAATTTTAATTACAGCAATTCTCCTTCTTCTCTCCCTCCCAGTTCTCGCTGGAGCAATTACCATACTTTTGACTGACCGTAACCTTAATACATCCTTCTTTGACCCCGCGGGAGGAGGAGACCCCATTCTTTACCAACATCTCTTTNNNNNNNNNNNNNNN>CASENT0120389_Pheidole_spinosaNNNNNNNNNNNNNNNNNNNNNNNNNNNNNNNNNNNTAATTGGATCCTCTATGAGATTAATTATTCGTTTAGAACTCGGATCCTGCAACTCCTTAATTAATAATGATCAAATTTATAACTCTTTAGTTACAAGACACGCTTTCATTATAATTTTCTTTATAGTAATACCTTTCATAATTGGAGGATTTGGTAATTTCCTAGTCCCTTTAATACTTGGTTCCCCAGATATAGCCTACCCACGAATAAATAATATGAGATTCTGACTACTCCCCCCCTCCATTACTCTCCTCTTATTAGGAAGATTCATTAATTCAGGTGCCGGAACNNNNNNNNNNNNNNNNNNNNNNNNNNNNNNNNNNNNNNNNNNNNNNNNNNNNNNNNNNNNNNNNNNNNNNNNNNNNNNNNNNNNNNNNNNNNNNNNNNNNNNNNNNNNNNNNNNNNNNNNNNNNNNNNNNNNNNNNNNNNNNNNNNNNNNNNNNNNNNNNNNNNNNNNNNNNNNNNNNNNNNNNNNNNNNNNNNNNNNNNNNNNNNNNNNNNNNNNNNNNNNNNNNNNNNNNNNNNNNNNNNNNNNNNNNNNNNNNNNNNNNNNNNNNNNNNNNNNNNNNNNNNNNNNNNNNNNNNNNNNNNNNNNNNNNNNNNNNNNNNNNNNNNNNNNNNNNNNNNNNNNNNNNNNN>CASENT0077933_Pheidole_spinosaNNNNNNNNNNNNNNNNNNNNNNNNNNNNNNNGGAAATATTGGATCCTCTATGAGATTAATTATTCGATTAGAACTCGGATCCTGCAACTCCTTAATTAATAATGATCAAATTTATAACTCTTTAGTTACAAGGCACGCTTTCATTATAATTTTCTTTATAGTAATACCTTTCATAATTGGAGGATTTGGTAATTTCCTAGTCCCTTTAATACTTGGTTCCCCAGATATAGCCTACCCACGAATAAATAATATGAGATTCTGACTACTTCCTCCCTCCATTACTCTCCTCTTATTAGGGAGATTCATTAATTCANNNNNNNNNNNNNNNNNNNNNNNNNNNNNNNNNNNNNNNNNNNNNNNNNNNNNNNNNNNNNNNNNNNNNNNNNNNNNNNNNNNNNNNNNNNNNNNNNNNNNNNNNNNNNNNNNNNNNNNNNNNNNNNNNNNNNNNNNNNNNNNNNNNNNNNNNNNNNNNNNNNNNNNNNNNNNNNNNNNNNNNNNNNNNNNNNNNNNNNNNNNNNNNNNNNNNNNNNNNNNNNNNNNNNNNNNNNNNNNNNNNNNNNNNNNNNNNNNNNNNNNNNNNNNNNNNNNNNNNNNNNNNNNNNNNNNNNNNNNNNNNNNNNNNNNNNNNNNNNNNNNNNNNNNNNNNNNNNNNNNNNNNNNNNNNNNNNNNNNN>CASENT0457127_Pheidole_spinosaTATACTCTACTTTATCTTTGCAATCTGATCTGGAATAATTGGATCCTCTATGAGATTAATTATTCGATTAGAACTCGGATCCTGCAACTCCTTAATTAATAATGATCAAATTTATAACTCTTTAGTTACAAGACACGCTTTCATTATAATTTTCTTTATAGTAATACCTTTCATAATTGGAGGATTTGGTAATTTCCTAGTCCCTTTAATACTTGGTTCCCCAGATATAGCCTATCCACGAATAAATAATATGAGATTCTGACTACTCCCCCCCTCCATTACTCTCCTCTTATTAGGAAGATTAATTAATTCAGGGGCCGGAACTGGATGAACAGTTTATCCCCCCTTAGCCTCTAATATTTTCCATAGAGGGGCCTCTATTGACCTCTCAATTTTCTCCCTACATATTGCAGGAATATCTTCCATCCTTGGGGCTATTAATTTTATTGCCACAATTATTAATATACATCATAAAAATTTTACTATAGATAAAATTCCTTTACTAGTTTGATCAATTTTAATTACAGCAATTCTCCTTCTTCTCTCCCTCCCAGTTCTCGCTGGAGCAATTACCATACTTTTAACTGACCGTAACCTTAATACATCCTTCTTTGACCCCGCGGGAGGAGGGGACCCCATTCTTTACNNNNNNNNNNNNNNNNNNNNNNNNNNN>CASENT0155938_Pheidole_spinosaTATACTCTACTTTATCTTTGCAATCTGATCTGGAATAATTGGATCCTCTATGAGATTAATTATTCGATTAGAACTCGGATCCTGCAACTCCTTAATTAATAATGATCAAATTTATAACTCTTTAGTTACAAGACACGCTTTCATTATAATTTTCTTTATAGTAATACCTTTCATAATTGGAGGATTTGGTAATTTCCTAGTCCCTTTAATACTTGGCTCCCCAGATATAGCCTACCCACGAATAAATAATATGAGATTCTGACTACTTCCTCCCTCCATTACTCTCCTCTTATTAGGAAGATTCATTAATTCAGGGGCCGGAACTGGATGAACAGTTTACCCCCCCTTAGCCTCTAATATTTTCCATAGGGGGGCCTCTATTGACCTCTCAATTTTCTCCCTACATATTGCAGGAATATCTTCCATCCTTGGAGCTATTAATTTTATTGCCACAATTATTAATATACATCATAAAAATTTTACTATAGATAAAATTCCTTTATTAGTTTGATCAATTTTAATTACAGCAATTCTCCTTCTTCTCTCCCTCCCAGTTCTCGCTGGAGCAATTACCATACTTTTAACCGACCGTAACCTTAATACATCCTTCTTTGACCCCGCGGGAGGAGGAGACCCCATTCTTTACCAACATCTCTTTNNNNNNNNNNNNNNN>CASENT0156543_Pheidole_spinosaTATACTCTACTTTATCTTTGCAATCTGATCTGGAATAATTGGATCCTCTATGAGATTAATTATTCGATTAGAACTCGGATCCTGCAACTCCTTAATTAATAATGATCAAATTTATAACTCTTTAGTTACAAGACACGCTTTCATTATAATTTTCTTTATAGTAATACCTTTCATAATTGGAGGATTTGGTAATTTCCTAGTCCCTTTAATACTTGGCTCCCCAGATATAGCCTACCCACGGATAAATAATATGAGATTCTGACTACTTCCTCCCTCCATTACTCTCCTCTTATTAGGAAGATTCATTAATTCAGGGGCCGGAACTGGATGAACAGTTTATCCCCCCTTAGCCTCTAATATTTTCCATAGAGGGGCCTCTATTGACCTCTCAATTTTCTCCCTACATATTGCAGGAATATCTTCCATCCTTGGAGCTATTAATTTTATTGCCACAATTATTAATATACATCATAAAAATTTTACTATAGATAAAATTCCTTTATTAGTTTGATCAATTTTAATTACAGCAATTCTCCTTCTTCTCTCCCTCCCAGTTCTCGCTGGAGCAATTACCATACTTTTAACCGACCGTAACCTTAATACATCTTTCTTTGACCCCGCGGGAGGAGGAGACCCCATTCTTTACCAACATCTCTTTNNNNNNNNNNNNNNN>CASENT0204394_Pheidole_spinosaNNTACTCTACTTTATCTTTGCAATCTGATCTGGAATAATTGGATCCTCTATGAGATTAATTATTCGATTAGAACTCGGATCCTGCAACTCCTTAATTAATAATGATCAAATTTATAACTCTTTAGTTACAAGACACGCTTTCATTATAATTTTCTTTATAGTAATACCTTTCATAATTGGAGGATTTGGTAATTTCCTAGTCCCTTTAATACTTGGCTCCCCAGATATAGCCTACCCACGGATAAATAATATGAGATTCTGACTACTTCCTCCCTCCATTACTCTCCTCTTATTAGGAAGATTCATTAATTCAGGGGCCGGAACTGGATGAACAGTTTATCCCCCCTTAGCCTCTAATATTTTCCATAGGGGGGCCTCTATTGACCTCTCAATTTTCTCCCTACATATTGCAGGAATATCTTCCATCCTTGGAGCTATTAATTTTATTGCCACAATTATTAATATACATCATAAAAATTTTACTATAGATAAAATTCCTTTATTAGTTTGATCAATTTTAATTACAGCAATTCTCCTTCTTCTCTCCCTCCCAGTTCTCGCTGGAGCAATTACCATACTTTTAACCGACCGTAACCTTAATACATCCTTCTTTGACCCCGCGGGAGGAGGAGACCCCATTCTTTACCAACATCTCTTTNNNNNNNNNNNNNNN>CASENT0455749_Pheidole_spinosaNNNNNNNNNNNNNNNNNNNNNNNNNNNNNNNNNNNNAATTGGATCCTCTATGAGATTAATTATTCGATTAGAACTCGGATCCTGCAACTCCTTAATTAATAATGATCAAATTTATAACTCTTTAGTTACAAGGCACGCTTTCATTATAATTTTCTTTATAGTAATACCTTTCATAATTGGAGGATTTGGTAATTTCCTAGTCCCTTTAATACTTGGTTCCCCAGATATAGCCTACCCACGAATAAATAATATGAGATTCTGACTACTTCCTCCCTCCATTACTCTCCTCTTATTAGGAAGATTCATTAATTCAGGCGCCGGAACTGGATGAACTNNNNNNNNNNNNNNNNNNNNNNNNNNNNNNNNNNNNNNNNNNNNNNNNNNNNNNNNNNNNNNNNNNNNNNNNNNNNNNNNNNNNNNNNNNNNNNNNNNNNNNNNNNNNNNNNNNNNNNNNNNNNNNNNNNNNNNNNNNNNNNNNNNNNNNNNNNNNNNNNNNNNNNNNNNNNNNNNNNNNNNNNNNNNNNNNNNNNNNNNNNNNNNNNNNNNNNNNNNNNNNNNNNNNNNNNNNNNNNNNNNNNNNNNNNNNNNNNNNNNNNNNNNNNNNNNNNNNNNNNNNNNNNNNNNNNNNNNNNNNNNNNNNNNNNNNNNNNNNNNNNNNNNNNNNNNNNNNNNN>CASENT0120350_Pheidole_spinosaTATACTCTACTTTATCTTTGCAATCTGATCTGGAATAATTGGATCCTCTATGAGATTAATTATTCGATTAGAACTCGGATCCTGCAACTCCTTAATTAATAATGATCAAATTTATAACTCTTTAGTTACAAGGCACGCTTTCATTATAATTTTCTTTATAGTAATACCTTTCATAATTGGAGGATTTGGTAATTTCCTAGTCCCTTTAATACTTGGTTCCCCAGATATAGCCTACCCACGAATAAATAATATGAGATTCTGACTACTTCCCCCCTCCATTACTCTCCTCTTATTAGGAAGATTCATTAATTCAGGCGCCGGAACTGGATGAACAGTTTATCCCCCCTTAGCCTCTAATATTTTCCATAGGGGGGCCTCTATTGACCTCTCAATTTTCTCCCTACATATTGCAGGAATATCTTCCATCCTTGGGGCTATTAATTTTATTGCCACAATTATTAATATACATCATAAAAATTTTACTATAGATAAAATTCCTTTATTAGTTTGATCAATTTTAATTACAGCAATTCTCCTTCTTCTCTCCCTCCCAGTTCTCGCTGGAGCAATTACCATACTTTTAACCGACCGTAACCTTAATACATCCTTCTTTGACCCCGCGGGAGGNNNNNNNNNNNNNNNNNNNNNNNNNNNNNNNNNNNNNNNNNNNNNN>CASENT0056724_Pheidole_spinosaNNNNNNNNNNNNNNNNNNNNNNNNNNNNNNNNNNNNNNTTGGGTCCTCTATGAGATTAATTATTCGATTAGAACTCGGATCCTGTAACTCCTTAATTAATAATGATCAAATTTATAACTCTTTAGTTACAAGGCACGCTTTCATTATAATTTTCTTTATAGTAATACCTTTCATAATTGGAGGATTTGGTAATTTCCTAGTACCTTTAATACTTGGTTCCCCAGATATAGCCTACCCACGAATAAATAATATAAGATTCTGACTACTTCCTCCTTCCATTACTCTCCTCTTATTAGGAAGATTCATCAATTCCGGGGCTGGAACTGGGTGAACAGTTTACCCCCCCTTAGCCTCTAATATTTTCCATAGGGGGGCCTCTATTGACCTCTCAATTTTCTCCCTACATATTGCAGGTATATCTTCCATTCTTGGAGCTATTAATTTTATTGCCACAATTATTAATATGCATCATAAAAATTTTACTATAGATAAAATTCCTTTATTAGTTTGATCAATTTTAATTACAGCAATTCTACTTCTTCTCTCCCTGCCAGTTCTCGCTGGAGCAATTACCATACTTTTAACCGACCGTAATCTTAATACATCCTTCTTCGACCCCGCAGGAGNNNNNNNNNNNNNNNNNNNNNNNNNNNNNNNNNNNNNNNNNNNNNNN>CASENT0156014_Pheidole_spinosaTATACTCTACTTTATCTTTGCAATCTGATCTGGAATAATTGGATCCTCTATGAGATTAATTATTCGATTAGAACTCGGATCCTGTAACTCCTTAATTAATAATGATCAAATTTATAACTCTTTAGTTACAAGACACGCTTTCATTATAATTTTCTTTATAGTAATACCTTTCATAATTGGAGGATTTGGTAATTTCCTAGTCCCTTTAATACTTGGTTCCCCAGATATAGCCTACCCACGAATAAATAATATAAGATTCTGACTACTCCCTCCTTCCATTACTCTCCTCTTATTAGGAAGATTCATTAATTCAGGAGCCGGAACTGGATGAACAGTTTACCCACCCTTAGCCTCTAATATTTTCCATAGGGGAGCCTCTATTGACCTCTCAATTTTCTCCCTACATATTGCAGGTATATCTTCCATTCTTGGAGCTATTAATTTTATTGCCACAATTATTAATATGCATCATAAAAATTTTACTATAGATAAAATTCCTTTATTAGTTTGATCAATTTTAATTACAGCAATTCTCCTTCTTCTCTCCCTGCCAGTTCTCGCTGGAGCAATTACCATACTTTTAACCGACCGCAACCTTAATACATCCTTCTTTGACCCCGCAGGAGGGGGAGACCCCATTCTTTACCAACATCTCTTTNNNNNNNNNNNNNNN>CASENT0125611_Pheidole_spinosaNNNNNNNNNNNNNNNNNNNNNNNNNNNNNNNNNNNNAATTGGGTCCTCTATGAGATTAATTATTCGATTAGAGCTCGGATCCTGTAATTCCTTAATTAATAATGATCAAATTTATAACTCTTTAGTTACAAGACACGCTTTCATTATAATTTTCTTTATAGTAATACCTTTCATAATTGGAGGATTTGGTAATTTTCTAGTCCCTTTAATACTTGGTTCCCCAGATATAGCCTACCCACGAATGAATAATATAAGATTCTGACTACTTCCTCCTTCCATTACTCTCCTCTTATTAGGTAGATTCATTAATTCAGGGGCCGGAACTGGGTGAACAGTTTACCCTCCCTTAGCTTCTAATATTTTCCATAGAGGGGCCTCTATTGACCTCTCAATTTTCTCCCTACATATTGCAGGAATATCTTCCATTCTTGGGGCTATTAATTTTATTGCCACAATTATTAATATGCATCATAAAAATTTTACTATAGATAAAATTCCTTTATTAGTTTGATCAATTTTAATTACAGCAATTCTCCTTCTTCTCTCCCTACCAGTTCTAGCTGGAGCAATTACCATACTTTTAACCGACCGTAACCTTAATACATCCTTCTTTGNNNNNNNNNNNNNNNNNNNNNNNNNNNNNNNNNNNNNNNNNNNNNNNNNNNNNNNNNNN>CASENT0137887_Pheidole_spinosaNNNNNNNNNNNNNNNNNNNNNNNNNNNNTCTGGAATAATTGGGTCCTCTATGAGATTAATTATTCGATTAGAACTCGGATCCTGTAACTCCTTAATTAATAATGATCAAATTTATAACTCTTTAGTTACAAGACACGCTTTCATTATAATTTTCTTTATAGTAATACCTTTCATAATTGGAGGATTTGGTAATTTTCTAGTCCCTTTAATACTTGGTTCCCCAGATATAGCCTACCCACGAATGAATAATATAAGATTCTGACTACTGCCTCCTTCCATTACTCTCCTCTTATTAGGAAGATTCATTAATTCAGGGGCCGGAACTNNNNNNNNNNNNNNNNNNNNNNNNNNNNNNNNNNNNNNNNNNNNNNNNNNNNNNNNNNNNNNNNNNNNNNNNNNNNNNNNNNNNNNNNNNNNNNNNNNNNNNNNNNNNNNNNNNNNNNNNNNNNNNNNNNNNNNNNNNNNNNNNNNNNNNNNNNNNNNNNNNNNNNNNNNNNNNNNNNNNNNNNNNNNNNNNNNNNNNNNNNNNNNNNNNNNNNNNNNNNNNNNNNNNNNNNNNNNNNNNNNNNNNNNNNNNNNNNNNNNNNNNNNNNNNNNNNNNNNNNNNNNNNNNNNNNNNNNNNNNNNNNNNNNNNNNNNNNNNNNNNNNNNNNNNNNNNNNNNNNNNNNNNNN>CASENT0107826_Pheidole_spinosaNATACTCTACTTTATCTTTGCAATCTGATCTGGAATAATTGGGTCCTCTATGAGATTAATTATTCGATTAGAACTCGGATCCTGTAATTCCTTAATTAATAATGATCAAATTTATAACTCTTTAGTTACAAGACACGCTTTCATTATAATTTTCTTTATAGTAATACCTTTCATAATTGGAGGATTTGGTAATTTTCTAGTCCCTTTAATACTTGGTTCCCCAGATATAGCCTACCCACGAATGAATAATATAAGATTCTGACTACTTCCTCCTTCCATTACTCTCCTCTTATTAGGAAGATTCATTAATTCAGGGGCCGGAACTGGGTGAACAGTTTACCCTCCCTTAGCCTCTAATATTTTCCATAGAGGAGCCTCTATTGACCTCTCAATTTTCTCCCTACATATTGCAGGAATATCTTCCATTCTTGGGGCTATTAATTTTATTGCCACAATTATTAATATGCATCATAAAAATTTTACTATAGATAAAATTCCTTTATTAGTTTGATCAATTTTAATTACAGCAATTCTCCTTCTTCTCTCTCTACCAGTTCTAGCTGGAGCAATTACTATACTTTTAACCGACCGTAACCTTAATACATCCTTCTTTGACCCCGCAGGAGGGGGAGACCCCATTCTTTACCAGCATCTCNNNNNNNNNNNNNNNNNN
